# Extract of *Phyllanthus emblica* L. fruit stimulates basal glucose uptake and ameliorates palmitate-induced insulin resistance through AMPK activation in C2C12 myotubes

**DOI:** 10.1186/s12906-024-04592-1

**Published:** 2024-08-02

**Authors:** Hai-yan Li, Chun-fei Li, Chun-hui Liu, Sun-ce Chen, Yi-fan Liu, Quan-he Lv, Wen Zhang

**Affiliations:** 1 School of Life Sciences, East China Normal University, 500 Dongchuan Road, Shanghai, 200241 China; 2 China National Institute of Standardization, 4 Zhichun Road, Beijing, 100191 China

**Keywords:** *Phyllanthus emblica* L., Glucose uptake, AMP-activated protein kinase, Insulin sensitivity, C2C12 myotubes, Palmitate

## Abstract

**Background:**

The fruit of *Phyllanthus emblica* L., a traditional medicine in China and India, is used to treat diabetes mellitus. Its water extract (WEPE) has demonstrated hypoglycemic effects in diabetic rats, but its mechanisms on glucose utilization and insulin resistance in skeletal muscle remain unclear. Therefore, this study aims to investigate the effects and underlying mechanisms of WEPE on glucose utilization and insulin resistance using C2C12 myotubes.

**Methods:**

Effects of WEPE on glucose uptake, GLUT4 translocation, and AMPK and AKT phosphorylation were investigated in C2C12 myotubes and palmitate-treated myotubes. An AMPK inhibitor and siRNA were used to explore the mechanisms of WEPE. Glucose uptake was determined using a 2-(N-(7-nitrobenz-2-oxa-1,3-diazol-4-yl) amino)-2-deoxyglucose (2-NBDG) uptake assay, and protein expression and GLUT4 translocation were assessed via western blotting.

**Results:**

In normal myotubes, WEPE significantly stimulated glucose uptake and GLUT4 translocation to the plasma membrane at concentrations of 125 and 250 µg/mL. This was accompanied by an increase in the phosphorylation of AMPK and its downstream targets. However, both compound C and AMPK siRNA blocked the WEPE-induced GLUT4 translocation and glucose uptake. Moreover, pretreatment with STO-609, a calcium/calmodulin-dependent protein kinase kinase β (CaMKKβ) inhibitor, inhibited WEPE-induced AMPK phosphorylation and attenuated the WEPE-stimulated glucose uptake and GLUT4 translocation. In myotubes treated with palmitate, WEPE prevented palmitate-induced insulin resistance by enhancing insulin-mediated glucose uptake and AKT phosphorylation. It also restored the insulin-mediated translocation of GLUT4 from cytoplasm to membrane. However, these effects of WEPE on glucose uptake and GLUT4 translocation were blocked by pretreatment with compound C.

**Conclusions:**

WEPE significantly stimulated basal glucose uptake though CaMKKβ/AMPK pathway and markedly ameliorated palmitate-induced insulin resistance by activating the AMPK pathway in C2C12 myotubes.

**Supplementary Information:**

The online version contains supplementary material available at 10.1186/s12906-024-04592-1.

## Background

*Phyllanthus emblica* L., commonly called ‘Amla’ or ‘Indian gooseberry’, is a popular fruit tree in the Euphorbiaceae family [1]. The fruit of *P. emblica* is rich in nutrients, such as calcium, vitamin-C, minerals, lysine, methionine, tryptophane, phosphorus and riboflavin, and widely consumed in subtropical areas [1]. Moreover, this fruit has traditionally been used to treat coughs, asthma, bronchitis, the cerebral and intestinal ailments, diabetes mellitus, coronary heart diseases as well as cancers in Chinese and Indian medicine [1–3]. It has been reported to have biological activities like anti-oxidant, anti-inflammatory, anti-cancer, anti-hyperlipidemic, anti-atherogenic, anti-diabetic and hepatoprotective properties [3–6]. *P. emblica* fruit and its extracts exhibit hypoglycemic effect in normal and in alloxan or STZ induced diabetic rats [7, 8]. Nevertheless, the underlying mechanisms of its hypoglycemic effect remain unclear.

Precise regulation of circulating glucose is vital for maintaining human health [9, 10]. Skeletal muscle plays a pivotal role in maintaining glucose homeostasis through glucose uptake via insulin-dependent and -independent pathways [11], which is vital for human health. It is the primary site for glucose uptake, especially in the postprandial state, and is responsible for about 80% of glucose disposal under euglycemic and hyperinsulinemic conditions [12–14]. This process, regulated by insulin, is key to the precise regulation of circulating glucose levels. Thus, skeletal muscle’s role in glucose uptake and its regulation by insulin are of significant importance in maintaining glucose homeostasis [14–17]. In insulin resistance states, the ability of skeletal muscle to take up glucose in response to insulin is markedly reduced. This leads to a persistent high level of blood glucose and can ultimately result in metabolic disorders such as type 2 diabetes [16, 18]. Since skeletal muscle is the principal site of insulin-stimulated glucose uptake, it is also considered to be the main cause of whole-body insulin resistance [13, 19]. Insulin resistance in skeletal muscles can occur decades before the development of β-cell failure and the appearance of type 2 diabetes symptoms [13, 19]. When the primary defect resides in skeletal muscle, remediating insulin resistance solely in the muscle is adequate to restore whole-body glucose homeostasis [11, 19]. Consequently, enhancing glucose uptake in skeletal muscles and augmenting insulin sensitivity within these muscles emerge as significant strategies for the prevention or mitigation of insulin resistance, hyperglycemia, and type 2 diabetes. Glucose uptake, the rate limiting step in glucose metabolism, is mediated by GLUT4 and can be activated in skeletal muscle by two separate and distinct signaling pathways; one stimulated by insulin through insulin receptor substrate 1 (IRS1)/phosphoinositide 3 kinase (PI3K) [20], and the other is activated by muscle contractions and exercise through activation of AMPK [21]. AMPK acts as a monitor for cellular energy levels and affects various metabolic processes including fatty acid synthesis, glucose uptake, and fatty acid oxidation [19, 22]. When the ratio of ADP to ATP increases, AMPK is activated through the phosphorylation of its α-subunit at Thr172 [19, 23]. This phosphorylation is regulated by upstream kinases, like calmodulin-dependent protein kinase and liver kinase B1 [19]. Exercise-stimulated glucose uptake is regulated by AMPK through its phosphorylation of AKT substrate of 160 kDa (AS160) and TBC1D1, which are two downstream targets [19]. AS160 increases the translocation of GLUT4 from vesicles to the plasma membrane [24]. Furthermore, AMPK promotes glucose uptake in skeletal muscle cells by activating p38 mitogen-activated protein kinase (MAPK) through phosphorylation [25]. This activation leads to the phosphorylation of various transcription factors and coactivators involved in carbohydrate metabolism by p38 MAPK [25, 26]. In addition to regulating glucose uptake, AMPK also enhances insulin sensitivity in skeletal muscle [19].

The water extract of *P. emblica* fruit (WEPE) exhibits anti-hyperglycemic effects in diabetic animals, but the underlying mechanism is still unclear. It is uncertain whether it decreases hyperglycemia by stimulating glucose uptake in skeletal muscle or by ameliorating insulin resistance within the same tissue. Therefore, this study, using mouse skeletal muscle cells (C2C12 myotubes), was designed to investigate the effects and underlying mechanisms of WEPE on basal glucose uptake in normal C2C12 myotubes and on insulin resistance in palmitate-induced C2C12 myotubes.

## Methods

### Chemicals and reagents

FA-free bovine serum albumin (BSA), insulin (bovine), bicinchoninic acid (BCA), protein assay kit, and secondary antibodies were purchased from Yeasen Biotechnology (Shanghai, China). 3-(4,5-dimethylthiazol-2-yl)-2,5-diphenyltetrazolium bromide (MTT) and dimethyl sulfoxide (DMSO) were purchased from Sigma-Aldrich Chemical Co. (St. Louis, MO, USA). Compound C, 5-aminoimidazole-4-carboxamide ribonucleotide (AICAR) and STO-609 were purchased from Sellbeck chemicals (Houston, TX, USA). Mem-PER Plus Membrane Protein Extraction Kit and 2-NBDG were purchased from Thermo Fisher Scientific (Sunnyvale, CA, USA). Fetal bovine serum (FBS) was purchased from Bovogen Biologicals (East Keilor, VIC, Australia), and other cell culture materials, including Dulbecco’s modified eagle’s medium (DMEM), horse serum, antibiotic, antimycotic and trypsin solutions were obtained from Gibco (Gaithersburg, MD, USA). Antibodies against AMPKα, phospho-AMPKα (Thr172), acetyl-CoA carboxylase (ACC), phosphorACC (Ser79), phospho-AS160 (Ser588), phospho-p38 MAPK (Thr180/Tyr182), phosphoAKT (Ser473) and phospho-AKT (Thr308), and protein kinase C-θ (PKCθ) were purchased from Cell Signaling Technology (Danvers, MA, USA). Antibody against phospho-IRS1 (Tyr632) was obtained from Abcam (Cambridge, UK); and antibodies against AKT, GLUT4, Na^+^-K^+−^ATPase, p38 MAPK and β-actin were obtained from Proteintech (Wuhan, China).

### Plant material and preparation of the water extract of *P. emblica* fruits

*P. emblica* fruits were supplied by the Yimin Agricultural Products Development Co., Ltd (Chuxiong, Yunnan, China), and authenticated by Professor Hong-qing Li, School of Life Science, East China Normal University. A voucher specimen (No.: FPE. Yunnan. 2020) was deposited in the herbarium of East China Normal University (Shanghai, China). The dry powder of *P. emblica* fruit was extracted with distilled water (solid-liquid ratio 1:10) on a rotary shaker for 24 h under 28–29 ℃, then, was filtered through Whatman qualitative filter paper no. 1 (Sigma-Aldrich). After filtration, the filtrate was concentrated in a vacuum rotary evaporator and freeze-dried to gain the extract (WEPE). The extraction process resulted in a yield of 33.8%, and the extract was stored at drying oven before use.

The stock solution of WEPE (100 mg/mL) was prepared by dissolving the extract in DMSO and filtering it through a sterile syringe filter with a pore diameter of 0.22 μm. Prior to treatment, the stock solution was further diluted to obtain various testing concentrations.

### Metabolite profiling and quantitative analysis of phenolic compounds

The metabolic profiling of the WEPE was analyzed using an ACQUITY UHPLC system (Waters Corporation Milford, USA) coupled with an AB SCIEX Triple TOF 5600 System (AB SCIEX, Framingham, MA). Both ESI positive and ESI negative ion modes were employed, with separation achieved on an ACQUITY UPLC BEH C 18 column (100 mm×2.1 mm, 1.7 μm). The binary gradient elution system consisted of (A) water (containing 0.1% formic acid, v/v), and (B) acetonitrile (containing 0.1% formic acid, v/v) and separation was achieved using the following gradient: 0 min, 95% A + 5% B; 2 min, 80% A + 20% B; 4 min, 75% A + 25% B; 9 min, 40% A + 60% B; 14 min, 100% B; 16 min, 100% B; 16.1 min, 95% A + 5% B; 18.1 min, 95% A + 5% B. The flow rate was 0.4 mL/min and column temperature was 45 °C. The injection volume was 5 µL.

Data acquisition was performed in full scan mode (m/z range from 70 to 1000) combined with IDA mode. Parameters of mass spectrometry were as follows: Ion source temperature, 550 °C (+) and 550 °C (−); ion spray voltage, 5500 V (+) and 4500 V (−); curtain gas of 35 PSI; declustering potential, 80 V (+) and − 80 V (−); collision energy, 10 eV (+) and − 10 eV (−); and interface heater temperature, 550 °C (+) and 550 °C (−). For IDA analysis, range of m/z was set as 50-1000, the collision energy was 30 eV. The raw data collected using UNIFI 1.8.1 software underwent preprocessing tasks such as baseline filtering, peak identification, and normalization using Progenesis QI v2.3. Post-processing, compounds were identified using their accurate mass number, secondary fragments, and isotope distribution with databases like HMDB, Lipidmaps, and METLIN for qualitative analysis.

In addition to the LC-MS analysis, phenolic compounds in the extract were quantified using UPLC-ESI-MS/MS. The data gathered from UPLC-ESI-MS/MS was processed using SCIEX OS-MQ Solutions software (Sciex, USA). The regression equations for the quantification are as follows:

$$\eqalign{& {\rm{Ellagicacid:}}\,{\rm{y}}\,{\rm{ = }}\,{\rm{1349}}{\rm{.46x}}\,{\rm{ + }}\,{\rm{3}}{\rm{.15}}\,{\rm{ \times }}\,{\rm{1}}{{\rm{0}}^{\rm{4}}} \cr & {\rm{(r}}\,{\rm{ = }}\,{\rm{0}}{\rm{.99158,}}\,{{\rm{r}}^{\rm{2}}}\,{\rm{ = }}\,{\rm{0}}{\rm{.98323)}} \cr}$$

$$\eqalign{& {\rm{Gallicacid:}}\,{\rm{y}}\,{\rm{ = }}\,{\rm{3}}{\rm{.98}}\,{\rm{ \times }}\,{\rm{1}}{{\rm{0}}^{\rm{4}}}{\rm{x}}\,{\rm{ - }}\,{\rm{9553}}{\rm{.97}} \cr & {\rm{(r}}\,{\rm{ = }}\,{\rm{0}}{\rm{.98979,}}\,{{\rm{r}}^{\rm{2}}}\,{\rm{ = }}\,{\rm{0}}{\rm{.97969)}} \cr}$$

$$\eqalign{& {\rm{trans}}\, - \,{\rm{Cinnamic acid: }}\,{\rm{y}}\,{\rm{ = }}\,{\rm{23995}}{\rm{.83x}}\,{\rm{ + }}\,{\rm{11803}}{\rm{.43}} \cr & \left( {{\rm{r}}\,{\rm{ = }}\,{\rm{0}}{\rm{.99261,}}\,{{\rm{r}}^{\rm{2}}}\,{\rm{ = }}\,{\rm{0}}{\rm{.98527}}} \right) \cr}$$

$$\eqalign{& {\rm{Quercitrin:}}\,{\rm{y}}\,{\rm{ = }}\,{\rm{6}}{\rm{.36}}\,{\rm{ \times }}\,{\rm{1}}{{\rm{0}}^{\rm{4}}}{\rm{x}}\,{\rm{ - }}\,{\rm{13707}}{\rm{.47}} \cr & {\rm{(r}}\,{\rm{ = }}\,{\rm{0}}{\rm{.99250,}}\,{{\rm{r}}^{\rm{2}}}\,{\rm{ = }}\,{\rm{0}}{\rm{.98506)}} \cr}$$

All LC-MS, UPLC-ESI-MS/MS analyses and data processing were performed by Shanghai Luming Biotechnology Co., Ltd.

### Cell culture and differentiation

The C2C12 mouse myoblasts were obtained from The National Center for Drug Screening (Shanghai, China). C2C12 mouse myoblasts were cultured in DMEM, supplemented with 10% (v/v) FBS, 100 U/mL streptomycin, and 100 U/mL penicillin, and maintained at 37 °C with 5% CO_2_. The cells were seeded into cell culture plates at a density of 5 × 10^4^ cells/mL. After reaching approximately 70% confluence (about 24 h), the medium was replaced to DMEM supplemented with 2% (v/v) horse serum and was refreshed after 2, 4 and 6 days of culture. After 6–7 days, the differentiation of C2C12 mouse myoblasts into myotubes was complete, at which point the experiments were initiated.

### Palmitate solution preparation

Palmitate was dissolved in 100% ethanol before being diluted in DMEM containing 2% fatty acid-free BSA. The control group was treated with an equivalent volume of ethanol added to the BSA-DMEM solution. All solutions were filtered, aliquoted, and stored at 4 °C for future use.

### MTT assay

We utilized an MTT assay to evaluate the effect of WEPE on cell viability. C2C12 myoblasts were cultured in 96-well plates and differentiated into myotubes. These myotubes were incubated in DMEM with 0.2% BSA for 6 h, followed by treatment with varying WEPE concentrations. After incubation, 20 µL of 3 mg/mL MTT was added to each well and treated for 2.5 h at 37 °C. The MTT formazan crystals were dissolved by adding 200 µL of dimethyl sulfoxide to each well and shaking until dissolution. Then, the absorbance of each well was measured at 490 nm using a microplate spectrophotometer and cell viability was calculated using the following formula:

$$\:Cell\:Viability=\frac{{OD}_{Sample}}{{OD}_{Control}}\times\:100\%$$

### Glucose consumption assay

Glucose consumption was determined using a glucose oxidase assay kit. Briefly, C2C12 myotubes were incubated in DMEM containing 0.2% BSA for 6 h, followed by treatment with different doses of WEPE for 12, 24, and 48 h. Glucose concentrations in the medium were measured post-treatment using a glucose oxidase assay kit according to the manufacturer’s protocol. Glucose consumption was then calculated by subtracting the post-treatment glucose concentration from the initial concentration in the culture medium.

### 2-NBDG uptake assay

Cell glucose uptake was assessed by measuring the uptake of 2-NBDG. Myotubes were cultured in black 96-well plates and subjected to various concentrations of WEPE for specified durations. An hour prior to cell harvest, the myotubes were rinsed with warm sterile PBS (37 °C) and incubated in glucose-free DMEM supplemented with 0.2% BSA. After an hour, the myotubes were washed again with warm sterile PBS and exposed to a medium containing 80 µM 2-NBDG for 30 min. Following another wash with warm sterile PBS, the fluorescence intensity of each well was measured at an excitation wavelength of 485 nm and an emission wavelength of 520 nm. The formula for calculating cell glucose uptake is as follows:

$$\:\text{G}\text{l}\text{u}\text{c}\text{o}\text{s}\text{e}\:\text{U}\text{p}\text{t}\text{a}\text{k}\text{e}=\frac{{\text{F}\text{I}}_{\text{S}\text{a}\text{m}\text{p}\text{l}\text{e}}-{\text{F}\text{I}}_{\text{B}\text{l}\text{a}\text{n}\text{k}}}{{\text{F}\text{I}}_{\text{C}\text{o}\text{n}\text{t}\text{r}\text{o}\text{l}}-{\text{F}\text{I}}_{\text{B}\text{l}\text{a}\text{n}\text{k}}}$$

### Transfection with small-interfering RNA (siRNA)

C2C12 myotubes were transfected with AMPKα1 siRNA and Negative Control siRNA (60 nM; Shanghai GenePharma) using LipofectamineTM 3000 Reagent (Invitrogen) in DMEM medium, following the manufacturer’s protocol. After 48 h of transfection, the efficiency was evaluated by performing western blotting against the AMPK antibody.

AMPKα1 siRNA:

sense (5’-3’)UUUGAAAGACCAAAGUCGGCU.

antisense (5’-3’)CCGACUUUGGUCUUUCAAACA.

Negative Control siRNA:

sense (5’-3’)UUCUCCGAACGUGUCACGUTT.

antisense (5’-3’)ACGUGACACGUUCGGAGAATT.

### Western blotting

Following treatment, cells were rinsed with ice-cold PBS and collected in a lysis buffer (containing 150 mM sodium chloride, 1.0% Triton X-100, 0.5% sodium deoxycholate, 0.1% sodium dodecyl sulfate (SDS), and 50 mmol/L Tris; pH 8.0) supplemented with protease and phosphatase inhibitors. Protein concentrations were quantified using a BCA Protein Assay Kit. Proteins were then separated on 10% SDS-polyacrylamide gel electrophoresis (SDS-PAGE) gels and transferred onto nitrocellulose membranes. The membranes were blocked with a 5% non-fat dry milk solution for 1 h at room temperature, followed by overnight incubation at 4 °C with primary antibodies. After washing three times with Tris-buffered saline containing 0.1% Tween 20, the membranes were incubated with secondary antibodies for an hour at room temperature. Finally, the blots were washed and visualized using an Odyssey CLx Imaging System, and the results were analyzed with Image-Pro Plus Software.

### Statistical analysis

Results are presented as the mean ± standard deviation (SD). Statistically significant differences among experimental groups were determined by one-way analysis of variance followed by Dunnett’s multiple-comparisons tests using SPSS software (IBM, Armonk, New York, USA). *P* value < 0.05 was considered statistically significant, while *P* value < 0.01 was deemed extremely significant between groups.

## Results

### Metabolite profiling of WEPE and LC-MS/MS quantification of its phenolic compounds

The non-targeted LC-MS analysis of the WEPE detected 5965 peaks. Total ion chromatogram (TIC) curves in the positive and negative ion modes were shown in supplementary Fig. S1. After eliminating ion peaks with inaccurate qualitative results, a total of 2895 metabolites were identified (Supplementary Table S1). These metabolites were detected in both positive (2044 metabolites) and negative modes (851 metabolites), and classified into 237 categories at the Sub Class level (Supplementary Table S2).

The major categories included flavonoids (26.45%, 728 metabolites), diradylglycerols (13.19%, 21 metabolites), and hydrolyzable tannins (7.66%, 74 metabolites). Other significant categories comprised fatty acids and conjugates, carbohydrates and carbohydrate conjugates, glycerophosphocholines, benzoic acids and derivatives, quinones and hydroquinones, and flavonoid glycosides.

Among these categories, a significant proportion belonged to phenolic compounds, including flavonoids, hydrolyzable tannins, benzoic acids and derivatives, quinones and hydroquinones, flavonoid glycosides and many others.

Given the high proportion of phenolic compounds (41.01%) in the WEPE, a further quantitative analysis of these phenolic components was conducted using LC-MS/MS. The concentration of the phenolic compounds was calculated according to the peak area of detected components and standards. Ellagic acid was the predominant constituent in the WEPE extract, with a concentration of 6688.87 µg/g extract. Other notable components included gallic acid (579.92 µg/g extract), trans-Cinnamic acid (356.73 µg/g extract), and quercitrin (220.41 µg/g extrac), among others.

### WEPE enhanced glucose uptake and GLUT4 translocation in C2C12 myotubes

The potential cytotoxicity of WEPE on C2C12 myotubes was evaluated by MTT assay. As shown in Supplementary Figure S2, treatment of cells with 100, 125, 150, 200, 250 or 300 µg/mL WEPE for 48 h did not obviously affect viability. Consequently, WEPE concentrations less than 300 µg/mL were employed in the subsequent experiments.

**Fig. 1:**
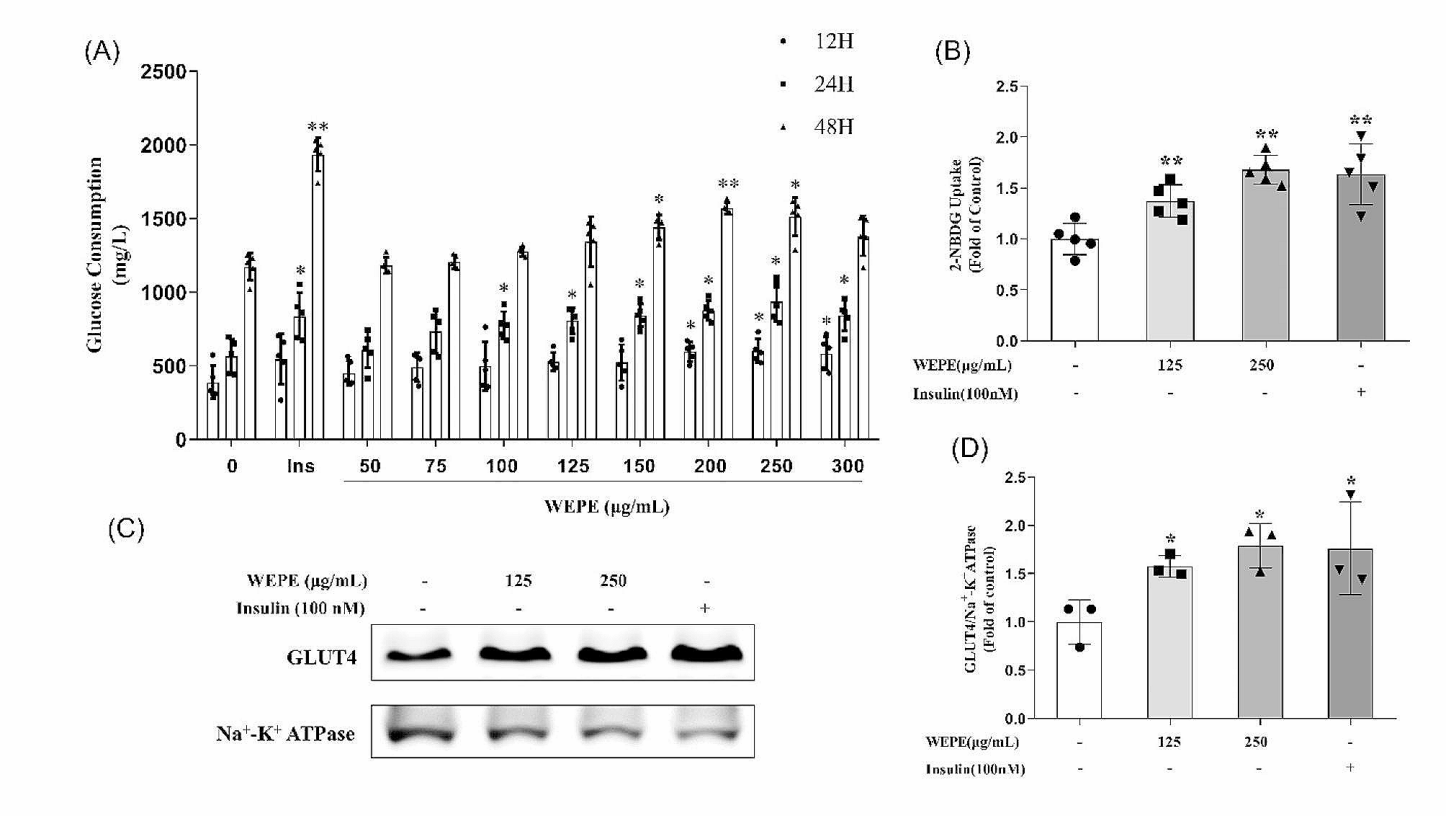
WEPE enhances glucose consumption, glucose uptake and GLUT4 translocation in C2C12 myotubes. **(A)** Myotubes were treated with various doses of WEPE for 12, 24, or 48 h, and glucose consumption was assayed by a commercial glucose oxidase assay kit (*n* = 5). **(B)** After incubation myotubes with WEPE (125 and 250 µg/mL) for 3 h or insulin (100 nmol/L) for 30 min, glucose uptake was measured using the 2-NBDG method (*n* = 5). In addition, western blotting **(C)** and quantification **(D)** of GLUT4 in cell membrane protein fractions was performed (*n* = 3). Values are expressed as mean ± SD. *P < 0.05, **P < 0.01, vs. control group

The effects of WEPE on glucose consumption and uptake in C2C12 myotubes were evaluated firstly. As results shown in Fig. 1A, WEPE significantly enhanced the glucose consumption of myotubes. Myotubes treated with various doses of WEPE for 12, 24, and 48 h exhibited a significant increase in glucose consumption compared to controls (*P* < 0.05 or 0.01). Then, a 2-NBDG uptake assay was employed to determine the effect of WEPE on glucose uptake. The results showed that WEPE at concentrations of 125 and 250 µg/mL significantly enhanced glucose uptake in C2C12 myotubes by 37.4 and 68%, respectively, compared to controls (*P* < 0.01, Fig. 1B). In addition, the effect of WEPE on GLUT4 translocation to the plasma membrane was evaluated. Consistent with the results of glucose uptake, WEPE markedly increased GLUT4 level in the plasma membrane of myotubes by 57.5 and 78.7%, respectively, compared to controls (*P* < 0.05, Fig. 1C and D).

### WEPE enhanced glucose uptake and GLUT4 translocation through AMPK activation

The insulin and AMPK signaling pathways play an important role in glucose uptake and GLUT4 translocation. To characterize the signaling pathways involved in WEPE-induced glucose uptake and GLUT4 translocation in C2C12 myotubes, the phosphorylation levels of proteins involved in the insulin and AMPK pathways were examined using western blotting analysis (Supplementary Figure S3). The results showed that treatment myotubes with WEPE (125–250 µg/mL) for 4 h did not activate IRS1 (Tyr632) or AKT (Ser473) (Supplementary Figure S3C). However, WEPE markedly increased the phosphorylated levels of AMPK (Thr172) and its downstream protein ACC (Ser79) in a time-dependent manner (Supplementary Figure S3B).

**Fig. 2:**
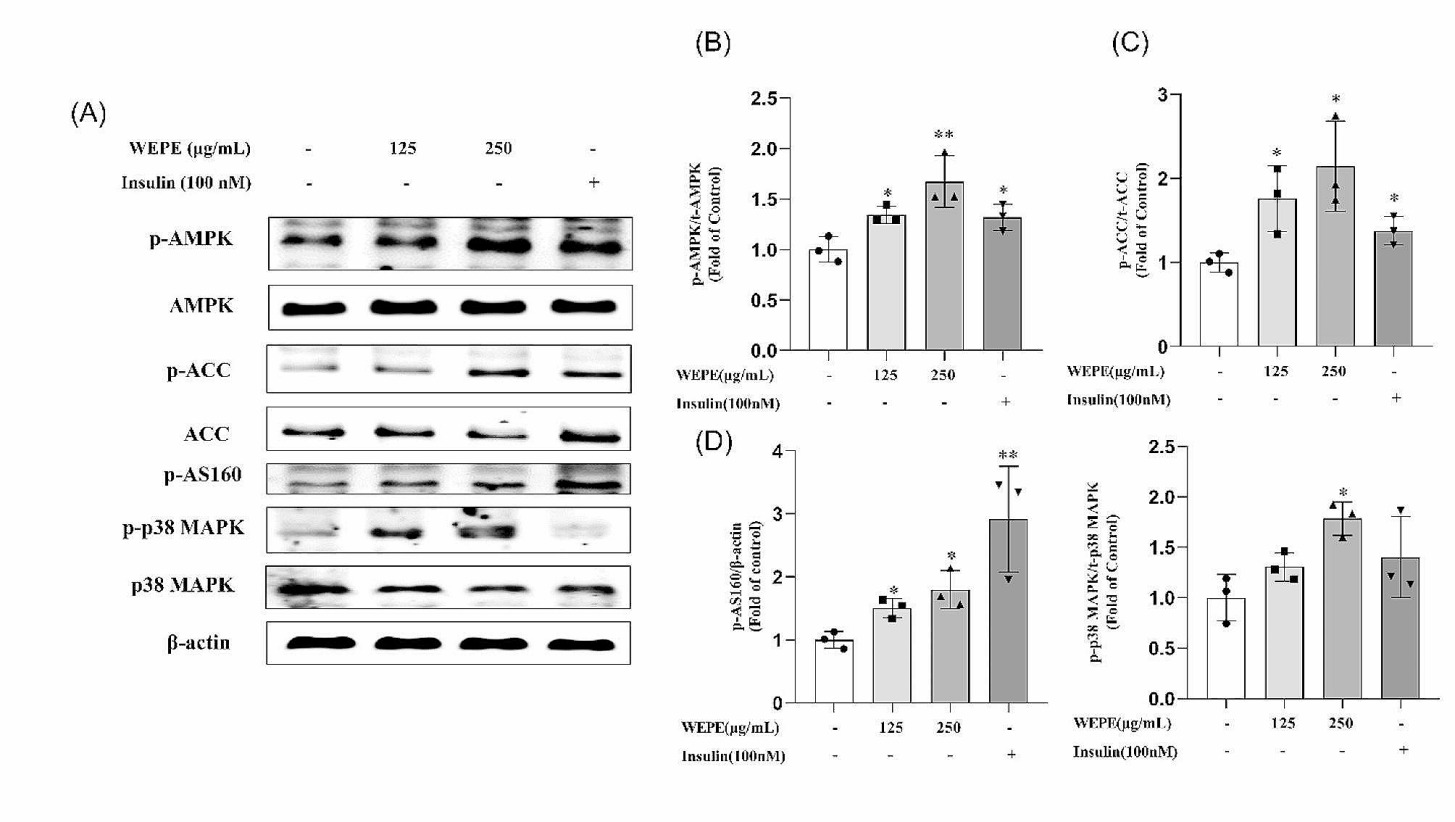
WEPE upregulated phosphorylation of AMPK, ACC, AS160, and p38 MAPK in C2C12 myotubes. Myotubes were treated with WEPE (125 and 250 µg/mL) for 3 h or insulin (100 nmol/L) for 30 min. Western blotting **(A)** and quantification **(B, C)** of phospho-AMPK and phospho- ACC in whole cell lysates. Western blotting **(A)** and quantification **(D)** of phospho-AS160 and phospho- p38 MAPK. Values are expressed as means ± SD (*n* = 3). *P < 0.05, **P < 0.01, vs. control group

Treatment C2C12 myotubes with WEPE (125 µg/mL) for 3 h significantly upregulated the levels of phosphorylated AMPK, ACC, AS160 and p38MAPK by 34.3%, 75.7%, 50.5% and 57.5% respectively, compared to the control group (Fig. 2A-D, *P* < 0.05). Meanwhile, treatment with 250 µg/mL WEPE resulted in an upregulation of the phosphorylated levels of AMPK, ACC, AS160 and p38 MAPK by 67.2%, 114.3%, 80.1% and 78.5%, respectively (Fig. 2A-D, *P* < 0.05 or 0.01). These results indicated that WEPE significantly activated the AMPK pathway.

**Fig. 3:**
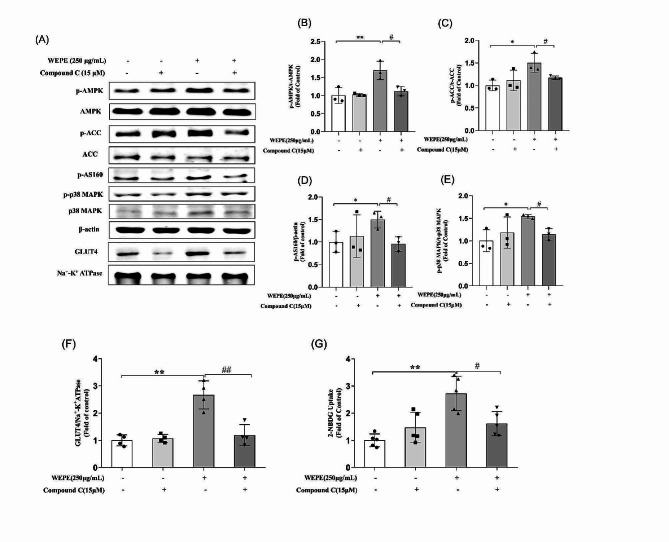
Effects of the AMPK inhibitor compound C on WEPE-induced AMPK activation, GLUT4 translocation and glucose uptake. Myotubes were incubated in the presence or absence of compound C (15 µmol/L) for 1 h followed by exposure to WEPE for 3 h. Western blotting of whole cell lysates was performed to detect phosphorylation of AMPK, ACC, AS160 and p38 MAPK. Western blotting **(A)** and quantification **(B-E)** of phospho-AMPK, phospho-ACC, phospho-AS160, phospho-p38 MAPK (*n* = 3). C2C12 myotubes were incubated in the presence or absence of compound C (15 µmol/L) for 1 h followed by exposure to WEPE for 3 h, and then GLUT4 in cell membrane proteins was detected. Western blotting **(A)** and quantification **(F)** of GLUT4 in cell membrane protein fractions (*n* = 4), glucose uptake **(G)** was measured using the 2-NBDG method (*n* = 5). Values are expressed as mean ± SD. *P < 0.05, **P < 0.01, vs. control group, ^#^*P* < 0.05, ^##^*P* < 0.01, vs. WEPE group

To determine whether WEPE stimulated glucose uptake and GLUT4 translocation through AMPK activation, compound C (an AMPK-specific inhibitor) and AMPK siRNA (targeting the α1 catalytic subunit) were used in subsequent experiments. C2C12 myotubes were pretreated with compound C (15 µM) for 1 h before a 3-hour treatment with WEPE (250 µg/mL) during which compound C was present. The results showed that the WEPE-stimulated GLUT4 translocation (Fig. 3A, F) and glucose uptake (Fig. 3G) significantly decreased in myotubes pretreated with compound C (*P* < 0.05 or 0.01 vs. WEPE treatment alone). Moreover, compound C blocked the upregulation of phosphorylation of AMPK, ACC, AS160 and p38 MAPK (Fig. 3A-E) induced by WEPE treatment (*P* < 0.05 or 0.01 vs. WEPE treatment alone).

**Fig. 4:**
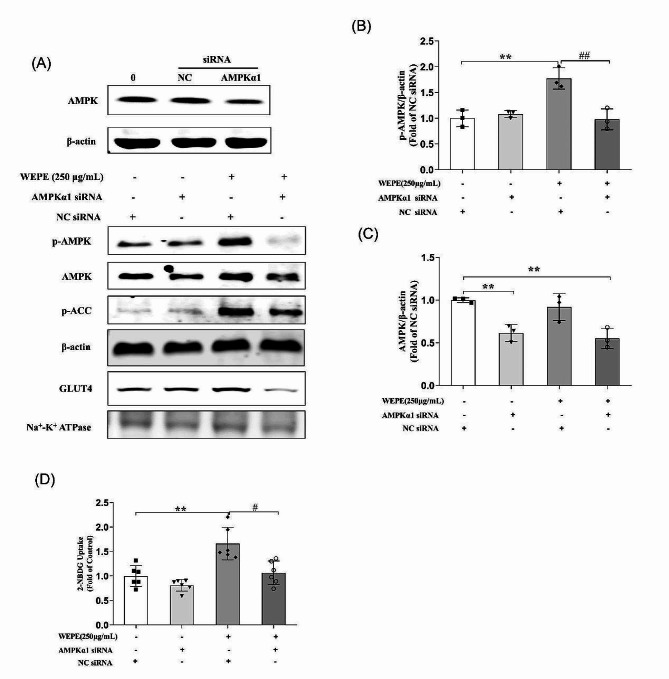
Effects of AMPKα1 siRNA on WEPE-induced AMPK activation, GLUT4 translocation and glucose uptake. C2C12 myotubes were transfected with negative control siRNA or AMPKα1 siRNA (60 nmol/L) for 48 h, followed by exposure to WEPE for 3 h. Cell membrane was extracted and GLUT4 in cell membrane proteins was detected. Western blotting of whole cell lysates was performed to detect phosphorylation of AMPK, ACC. Western blotting **(A)** and quantification **(B**, **C)** of phospho-AMPK, AMPK (*n* = 3). Glucose uptake **(D)** was measured using the 2-NBDG method (*n* = 6). Values are expressed as mean ± SD. **P < 0.01, vs. NC siRNA group, ^#^*P* < 0.05, ^##^*P* < 0.01, vs. WEPE group

To verify the functional significance of AMPK- dependence, AMPK siRNA (60 nM) was employed to reduce AMPK expression in C2C12 myotubes (Fig. 4A, C; reduce by 38.5% of control levels). Subsequent analysis of phosphorylation of AMPK and ACC, as well as GLUT4 translocation showed that AMPK siRNA, but not scrambled siRNA, significantly attenuated the ability of WEPE to activate AMPK (Fig. 4A, B; *P* < 0.01).Furthermore, the WEPE-induced GLUT4 translocation (Fig. 4A) and glucose uptake (Fig. 4D, *P* < 0.05) were also blunted by the siRNA-mediated reduction of AMPK level in C2C12 myotubes. These results indicated that WEPE stimulated glucose uptake in C2C12 myotubes via the AMPK pathway.

### CaMKKβ is involved in the WEPE-activated AMPK pathway in C2C12 myotubes

**Fig. 5:**
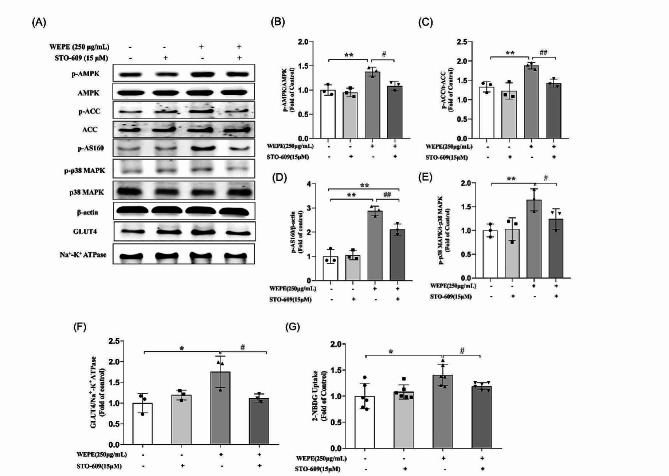
Effects of the CaMKKβ Inhibitor STO-609 on AMPK activation, GLUT4 translocation, and glucose uptake induced by WEPE. Myotubes were incubated in the presence or absence of STO-609 (15 µmol/L) for 1 h followed by exposure to WEPE for 3 h. Western blotting of whole cell lysates was performed to detect phosphorylation of AMPK, ACC, AS160 and p38 MAPK. Western blotting **(A)** and quantification **(B-E)** of phospho-AMPK, phospho-ACC, phospho-AS160 and phospho-p38 MAPK (*n* = 3). C2C12 cells were incubated in the presence or absence of STO-609 (15 µmol/L) for 1 h followed by exposure to WEPE for 3 h, and then GLUT4 in cell membrane proteins was detected. Western blotting **(A)** and quantification **(F)** of GLUT4 in membrane protein fraction (*n* = 3), glucose uptake **(G)** was measured using the 2-NBDG method (*n* = 6). Values are expressed as mean ± SD. *P < 0.05, **P < 0.01, vs. control group, #*P* < 0.05, ##*P* < 0.01, vs. WEPE group

Physiological AMPK activation involves phosphorylation of Thr-172 within the activation loop of the N-terminal kinase domain in the AMPKα catalytic subunit [27]. The upstream kinases of AMPK, CaMKKβ (Ca^2+^/calmodulin-dependent protein kinase β), are known to phosphorylate the Thr-172 of the AMPKα subunit [28]. To explore the possible role of CaMKKβ in WEPE-stimulated glucose uptake, a CaMKKβ inhibitor (STO-609) was employed. As results shown in Fig. 5, STO-609 blocked the WEPE-induced phosphorylation of AMPK and its downstream proteins ACC, AS160 and p38 MAPK (Fig. 5A-E; *P* < 0.05 or 0.01 vs. WEPE treatment alone), indicating that WEPE activated the AMPK pathway via CaMKKβ. Moreover, pretreatment with STO-609 inhibited both WEPE-induced GLUT4 translocation (Fig. 5A and F, *P* < 0.05 vs. WEPE treatment alone) and glucose uptake (Fig. 5G; *P* < 0.05 vs. WEPE treatment alone). These results indicated that WEPE enhanced glucose uptake through the CaMKKβ-AMPK-p38 MAPK-AS160 pathway.

### WEPE ameliorate palmitate -induced insulin resistance in C2C12 myotubes

Palmitate, a primary inducer of insulin resistance, has been widely reported to cause insulin resistance in hepatocytes and myotubes [29, 30]. In this study, we used palmitate to induce insulin resistance in C2C12 myotubes.

**Fig. 6:**
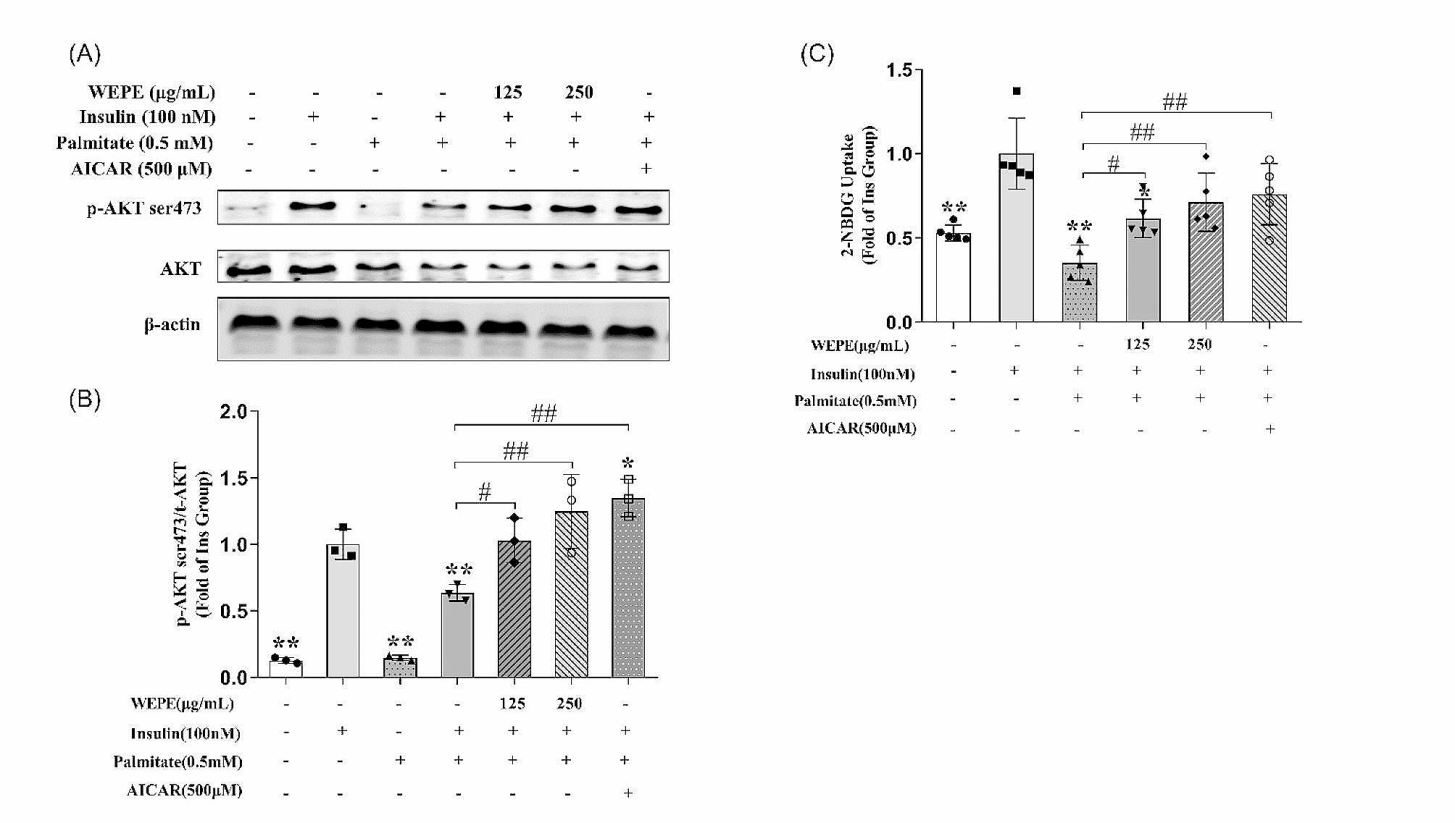
WEPE attenuates palmitate-induced insulin resistance in C2C12 myotubes. Differentiated C2C12 cells were stimulated for 24 h in the absence or presence of palmitate (0.5 mmol/L), and followed by exposure to 125 and 250 µg/mL WEPE for another 6 h (in the absence of palmitate). AICAR (500 µmol/L) was incubated for 1 h as positive control. Insulin (100 nmol/L) was added 30 min before harvest. Whole cell lysates were prepared and western blotting was performed to detect level of phospho-AKT (Ser 473). Western blotting **(A)** and quantification **(B)** of protein level of phospho-AKT (Ser473) (*n* = 3). Glucose uptake **(C)** was measured using the 2-NBDG method (*n* = 5). *P < 0.05, **P < 0.01 vs. insulin group, ^**#**^*P* < 0.05, ^**##**^*P* < 0.01 vs. Model group (Palmitate + Insulin group)

The myotubes were exposed to 500 µM palmitate for 24 h to establish an insulin resistance model, followed by treatment with WEPE at concentrations of 125 and 250 µg/mL for 6 h (in the absence of palmitate). To verify whether WEPE affects palmitate-induced insulin resistance, we performed a 2-NBDG uptake assay. Insulin was used to stimulate cell glucose uptake and insulin signaling. Our results (Fig. 6A, B, and C) showed that compared to the control, insulin significantly increased the phosphorylation of AKT and glucose uptake in C2C12 cells (*P* < 0.01). However, these effects were blocked when the myotubes were treated with 500 µM palmitate for 24 h (*P* < 0.05 or 0.01). Interestingly, WEPE (125 and 250 µg/mL) significantly reversed the palmitate-induced decrease in insulin-stimulated activation of AKT (Ser 473) by 62.1% and 96.4%, respectively (*P* < 0.05 or 0.01 vs. palmitate and insulin co-treatment group, Fig. 6A, B). Furthermore, WEPE (125 and 250 µg/mL) significantly reversed the decrease in insulin-stimulated glucose uptake caused by palmitate (*P* < 0.05 or 0.01, Fig. 6C). These findings suggest that WEPE may have potential therapeutic effects on insulin resistance.

### WEPE ameliorate palmitate-induced insulin resistance via AMPK pathway

**Fig. 7:**
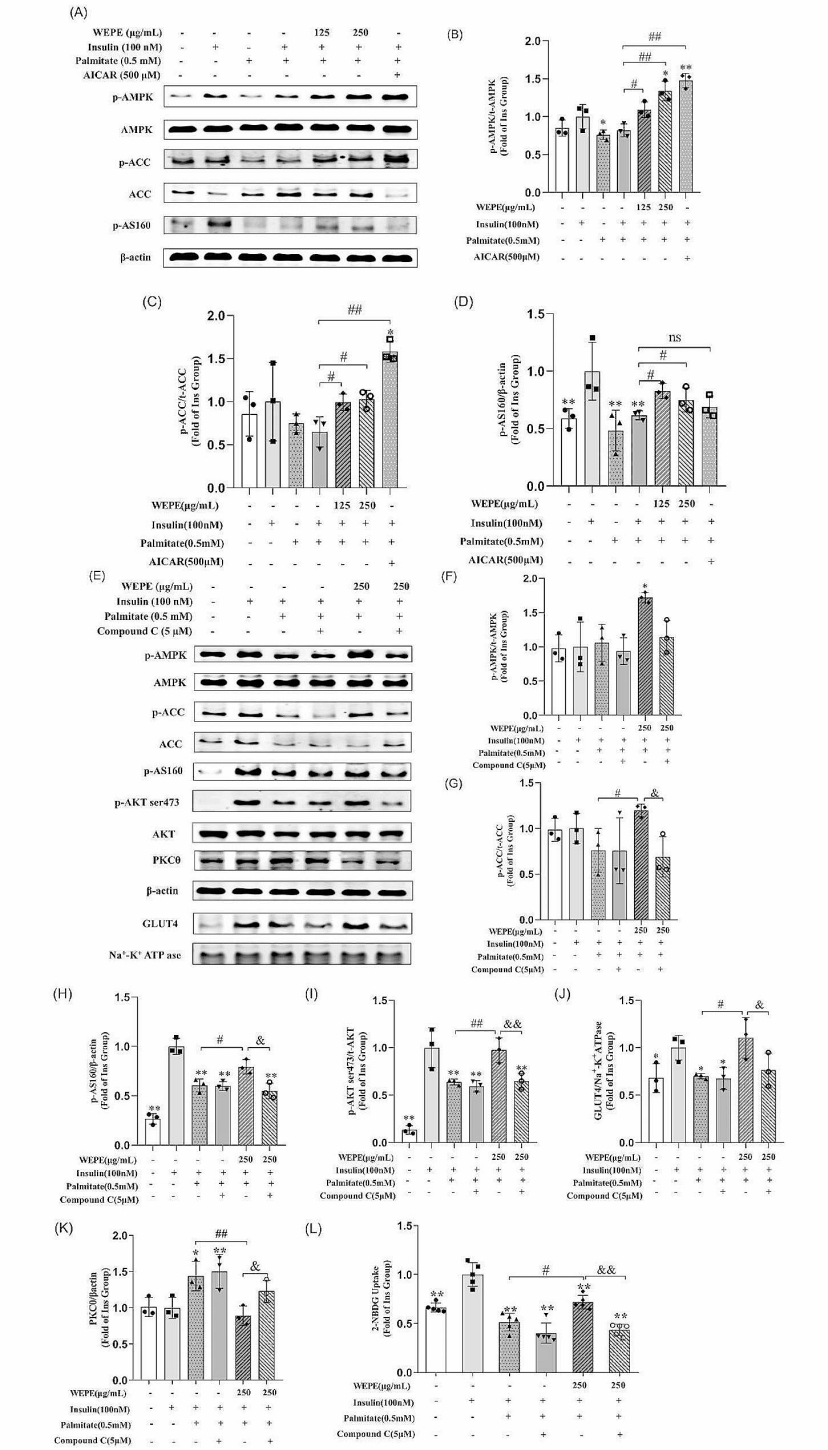
WEPE ameliorated palmitate-induced insulin resistance via AMPK activation in C2C12 myotubes. Differentiated C2C12 myotubes were treated with palmitate (0.5 mmol/L) for 24 h to induce insulin resistance. Then, compound C (5 µmol/L) was pretreated for 1 h, and followed by exposure to WEPE (250 µg/mL) for another 6 h. C2C12 myotube were stimulated with insulin (100 nmol/L) for 30 min before harvest. Western blotting **(A)** and quantification **(B-D)** of protein levels of phosho-AMPK, phosphor-ACC, phosho-AS160 (n = 3). Western blotting **(E)** and quantification **(F-H)** of protein levels of phosho-AMPK, phosphor-ACC, phosho-AS160 (n = 3). Western blotting **(E)** and quantification **(I-K)** of protein levels of phospho-AKT (Ser473), GLUT4 in membrane protein fraction, and PKCθ (n = 3). Glucose uptake **(L)** was measured using the 2-NBDG method (n = 5). *P<0.05, **P < 0.01 vs. insulin group. ^#^*P* < 0.05, ^##^*P* < 0.01 vs. Model group (Palmitate+Insulin group).  ^&^*P* < 0.05, ^&&^*P* < 0.01 vs. WEPE group

We further investigated whether the protective effects of WEPE against palmitate-induced insulin resistance in myotubes involved AMPK pathway. We did this by measuring the phosphorylation levels of AMPK, ACC, and AS160. As results shown in Fig. 7A-D, WEPE (125 and 250 µg/mL) significantly increased the phosphorylation of AMPK, ACC, and AS160 (*P* < 0.05 or 0.01), indicating that it significantly activated the AMPK pathway in palmitate-induced insulin resistant myotubes.

Based on these results, we hypothesized that WEPE attenuated palmitate-induced insulin resistance through an AMPK-dependent mechanism. To validate this hypothesis, we used compound C. As results shown in Fig. 7E-H, pretreatment with compound C significantly blocked the WEPE-induced phosphorylation of AMPK, ACC and AS160 (*P* < 0.05 or 0.01). We also evaluated the effects of compound C on the stimulatory effect of WEPE on insulin-stimulated phosphorylation of AKT, GLUT4 translocation and glucose uptake. As shown in Fig. 7E, I, J and L, palmitate significantly decreased the insulin-stimulated phosphorylation of AKT, GLUT4 translocation and glucose uptake. These effects were reversed by treatment with 250 µg/mL WEPE (*P* < 0.05 or 0.01, Fig. 7E, I, J and L). However, compound C prevented the restoration of AKT phosphorylation, GLUT4 translocation, and glucose uptake by WEPE treatment (*P* < 0.05 or 0.01). Moreover, compound C also reversed the inhibitory effect of WEPE on palmitate-induced PKCθ upregulation (*P* < 0.05, Fig. 7E and K). These results suggest that WEPE ameliorated palmitate-induced insulin resistance via AMPK pathway in C2C12 myotubes.

## Discussion

In the present study, WEPE enhanced GLUT4 translocation to the plasma membrane and subsequently stimulated basal glucose uptake through activating AMPK signaling pathway. Furthermore, WEPE significantly ameliorated insulin resistance in C2C12 myotubes induced by palmitate, also via the activation of the AMPK signaling pathway.

The translocation of GLUT4 is commonly regulated by the AMPK and insulin signaling pathways [27, 31]. In this study, WEPE treatment did not affect phosphorylated levels of IRS1 (Tyr632) or AKT (Ser473 and Thr308), indicating that WEPE had no effect on the insulin pathway at tested concentrations. However, WEPE was observed to significantly activate AMPK and promote phosphorylation of its downstream targets ACC, AS160 and p38 MAPK in a dose-dependent manner. These results suggest that AMPK activation might be associated with WEPE -stimulated glucose uptake. This is in agreement with previous studies that showed that AMPK activation enhances GLUT4 expression and translocation in skeletal muscle cells [21, 25]. To further confirm the role of AMPK in mediating the effects of WEPE, we used the AMPK inhibitor compound C and AMPKα1 siRNA. We found that both compound C and AMPKα1 siRNA blocked WEPE -induced phosphorylation of AMPK and its downstream targets ACC, AS160 and p38 MAPK. Moreover, WEPE-induced GLUT4 translocation and glucose uptake were abolished by compound C and AMPKα1 siRNA treatment. These results indicated that WEPE significantly enhanced GLUT4 translocation and subsequently stimulated basal glucose uptake in C2C12 myotubes via AMPK- p38MAPK-AS160 pathway.

The activity of AMPK is regulated by CaMKKβ, an upstream kinase that activates AMPK through the phosphorylation of the cyclic threonine 172 on its AMPKα subunit [32]. Our findings revealed that pre-incubation with STO-609, a CaMKKβ inhibitor, counteracted the WEPE-induced GLUT4 translocation and glucose uptake, as well as the activation of the AMPK pathway. These suggest that WEPE stimulated glucose uptake and activates AMPK through CaMKKβ. However, we also observed that WEPE-induced GLUT4 translocation was independent of the PI3K/Akt pathway, which is the canonical pathway for insulin-stimulated GLUT4 translocation. This suggests that WEPE might have a unique mechanism of action that differs from insulin. Further studies are needed to elucidate the molecular details of WEPE-induced AMPK activation and its interaction with other signaling pathways. In conclusion, our results indicated that WEPE significantly enhanced GLUT4 translocation and subsequently stimulated basal glucose uptake in C2C12 myotubes via CaMKKβ/AMPK/p38MAPK/AS160 pathway.

Skeletal muscle is a key target tissue for insulin and plays a crucial role in maintaining body glucose metabolism. Insulin resistance in skeletal muscle impairs the ability of insulin to regulate glucose homeostasis, leading to glucose intolerance and hyperglycaemia [33].

Insulin resistance, a condition often linked to chronic overnutrition, results in increased levels of circulating glycerol and free fatty acids, which are key contributors to the development of type 2 diabetes [11, 18, 34]. These free fatty acids, entering cells through diffusion or transport proteins like CD36, are stored as intramyocellular lipids [11]. However, excessive lipid accumulation in skeletal muscle has been associated with insulin resistance in numerous studies [16]. This lipid accumulation leads to a buildup of harmful metabolites such as diacylglycerol and ceramide, which interfere with insulin signal transduction [16, 35–37].

In this study, we used palmitate, a common dietary saturated free fatty acid known to induce insulin resistance [29, 30], to establish an insulin-resistant cell model. Our findings align with previous studies [29, 30, 38], showing that palmitate inhibited insulin-induced glucose uptake in myotubes by disrupting the insulin signaling pathway. Specifically, palmitate decreased AKT and AS160 phosphorylation, inhibited GLUT4 translocation to the plasma membrane, and upregulated PKCθ, a negative regulator of insulin signaling. However, our study also revealed that treatment of palmitate-exposed myotubes with WEPE significantly counteracted the inhibitory effects of palmitate. WEPE restored insulin-stimulated glucose uptake, GLUT4 translocation, and AKT activation, effectively reversing the insulin resistance induced by palmitate. These results indicate that WEPE mitigates palmitate-induced insulin resistance in C2C12 myotubes.

In addition to boosting insulin-stimulated glucose uptake, WEPE also triggered the AMPK pathway in insulin-resistant myotubes caused by palmitate. Previous studies has shown that AMPK activation can increase glucose uptake independently of insulin, improve insulin resistance in skeletal muscle, and lower plasma glucose and lipid levels [21, 39, 40]. To explore whether AMPK activation was responsible for the beneficial effects of WEPE on insulin resistance, we employed compound C in this study. Our results showed that pre-treatment with compound C negated the effects of WEPE on insulin-stimulated GLUT4 translocation and glucose uptake. Furthermore, compound C treatment hindered WEPE’s ability to restore the insulin-stimulated phosphorylation of AKT and AS160, which was compromised by palmitate. In addition, compound C treatment counteracted the inhibitory effect of WEPE on the upregulation of PKCθ triggered by palmitate. These findings suggest that AMPK activation is crucial for the protective effects of WEPE against palmitate-induced insulin resistance in C2C12 myotubes.

**Fig. 8:**
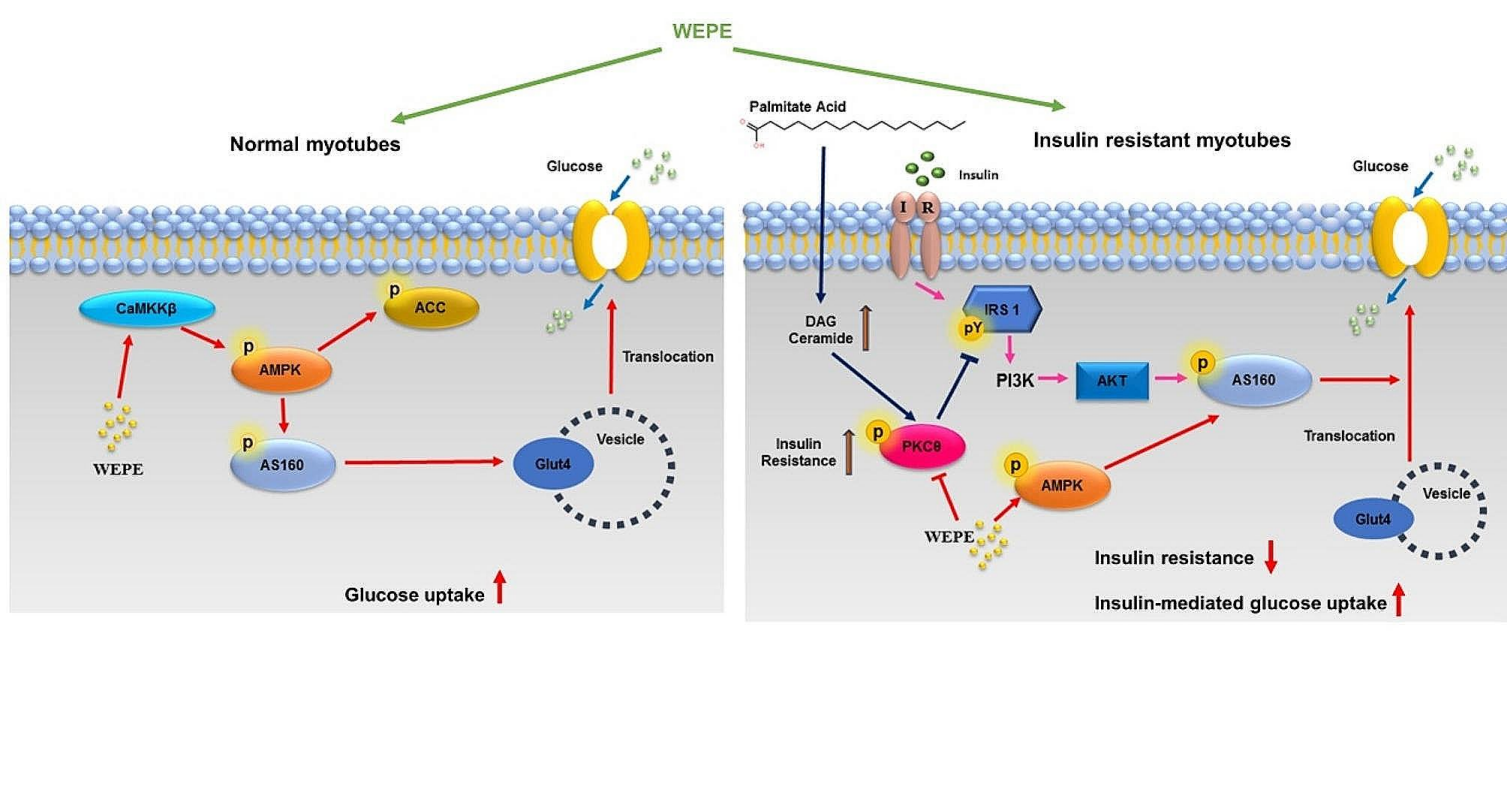
The potential mechanisms of WEPE on basal glucose uptake and palmitate-induced insulin resistance in C2C12 myotubes. This extract stimulates basal glucose uptake through CaMKKβ-AMPK pathway, and increases insulin-mediated glucose uptake through AMPK activation in palmitate-treated C2C12 myotubes

## Conclusions

The results of this study demonstrate that WEPE enhances basal glucose uptake and ameliorates palmitate-induced insulin resistance through the AMPK pathway in C2C12 myotubes (Fig. 8). These findings suggest that WEPE may have potential therapeutic effects for diabetes by enhancing glucose utilization and insulin sensitivity in skeletal muscle. Our study provides new insights into the anti-diabetic effects of WEPE and the fruit of *Phyllanthus emblica* L., which could be developed as a promising anti-diabetic agent.

### Electronic supplementary material

Below is the link to the electronic supplementary material.

Supplementary Material 1

Supplementary Material 2

Supplementary Material 3

Supplementary Material 4

Supplementary Material 5

Supplementary Material 6

Supplementary Material 7

## Data Availability

The data was included in figures of the manuscript, and the raw data for this study are available upon reasonable request to the corresponding author.

## Abbreviations

2-NBDG: 2-(N-(7-nitrobenz-2-oxa-1,3-diazol-4-yl) amino)-2-deoxyglucose
ACC: Acetyl-CoA carboxylase
AICAR: 5-aminoimidazole-4-carboxamide ribonucleotide
AMPK: AMP-activated protein kinase
AS160: Akt substrate of 160 kDa
BCA: Bicinchoninic acid
BSA: Bovine serum albumin
CAMK: Calmodulin-dependent protein kinase
CaMKKβ: Ca^2+^/calmodulin-dependent protein kinase β
DAG: Diacylglycerol
DMEM: Dulbecco’s modified Eagle’s medium
DMSO: Dimethyl sulfoxide
FBS: Fetal bovine serum
GLUT4: Glucose transporter 4
IRS1: Insulin receptor substrate 1
MAPK: Mitogen-activated protein kinase
MTT: 3-(4,5-dimethylthiazol-2-yl)-2,5-diphenyltetrazolium bromide
PBS: Phosphate-buffered saline
PI3K: Phosphoinositide 3 kinase
PKB/AKT: Protein Kinase B
SDS-PAGE: SDS-polyacrylamide gel electrophoresis
WEPE: Water extract of *Phyllanthus emblica* L. fruit
