# Supplementary figures and images for: Extract of Phyllanthus emblica L. fruit stimulates basal glucose uptake and ameliorates palmitate-induced insulin resistance through AMPK activation in C2C12 myotubes
Source: BMC Complement Med Ther. 2024 Aug 2;24:296. doi: 10.1186/s12906-024-04592-1 (PMC11295889; doi:10.1186/s12906-024-04592-1)

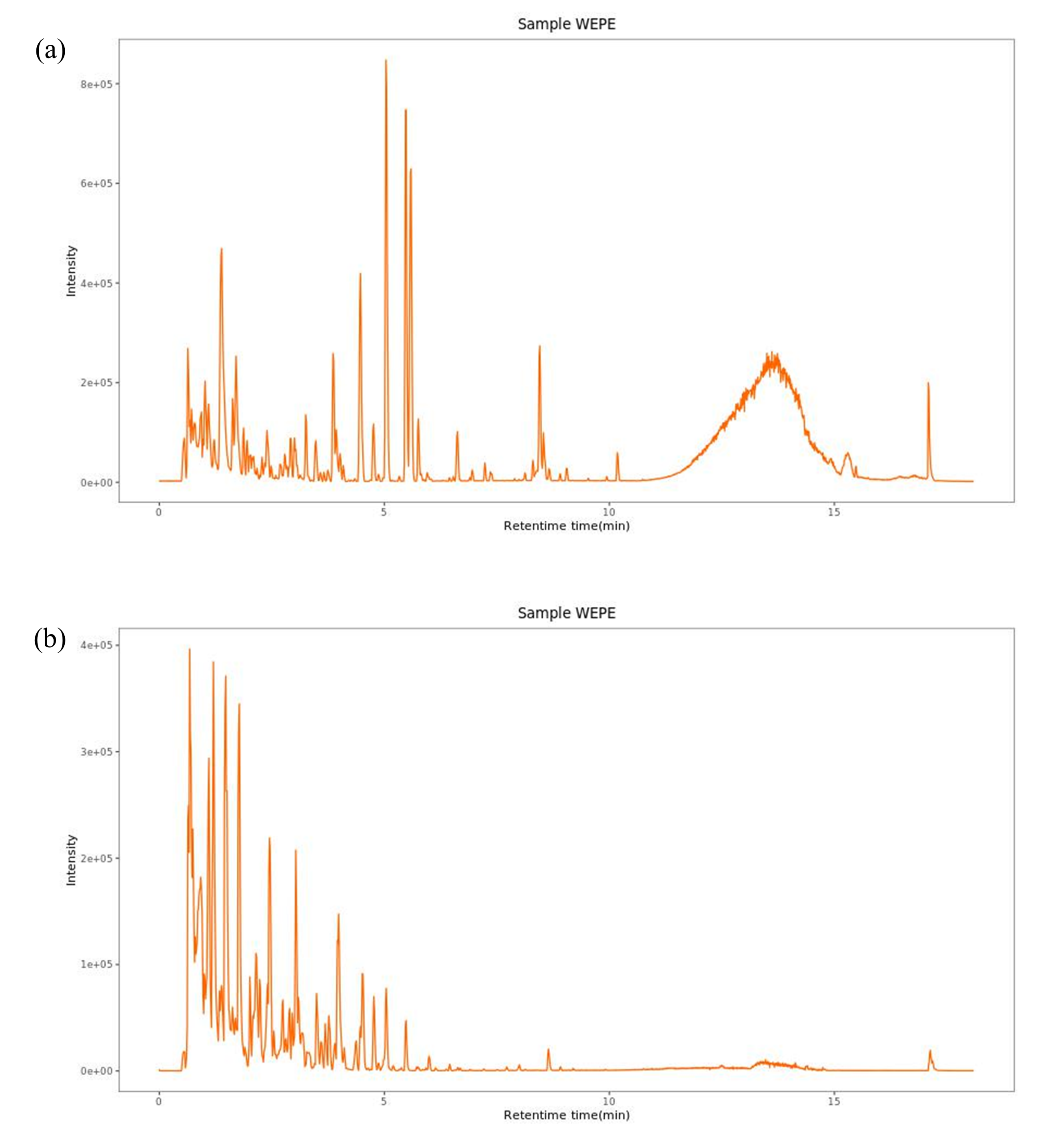


Fig. S1 The total ion chromatogram of WEPE, represented in both positive (a) and negative (b) ion modes

Supplement: Supplementary file 1 — Supplementary Material 1 [file 12906_2024_4592_MOESM1_ESM.docx]
