# Supplementary material for: Extract of Phyllanthus emblica L. fruit stimulates basal glucose uptake and ameliorates palmitate-induced insulin resistance through AMPK activation in C2C12 myotubes
Source: BMC Complement Med Ther. 2024 Aug 2;24:296. doi: 10.1186/s12906-024-04592-1 (PMC11295889; doi:10.1186/s12906-024-04592-1)
Supplement: Supplementary file 2 — Supplementary Material 2 [file 12906_2024_4592_MOESM2_ESM.docx]

**Supplementary Tables**

Table S1. The identification of metabolites from WEPE.

See the attached xlsx. File: Supplementary Table S1. The identification of metabolites from WEPE

Table S2. Numbers of metabolites at the sub class level

| **Sub Class** | **Area%** | **Numbers** |
| --- | --- | --- |
| Flavonoids | 26.4509% | 728 |
| Diradylglycerols | 13.1866% | 21 |
| Hydrolyzable tannins | 7.6594% | 74 |
| Fatty Acids and Conjugates | 6.4765% | 315 |
| Carbohydrates and carbohydrate conjugates | 5.9209% | 142 |
| Glycerophosphocholines | 4.7874% | 48 |
| Benzoic acids and derivatives | 4.1541% | 25 |
| Sphingoid bases | 3.4625% | 15 |
| Glycerophosphoethanolamines | 2.8876% | 56 |
| Glycerophosphoserines | 2.5259% | 20 |
| Fatty esters | 2.5221% | 90 |
| Sterols | 2.3855% | 34 |
| Amino acids, peptides, and analogues | 1.7706% | 93 |
| Isoprenoids | 1.3213% | 82 |
| Quinones and hydroquinones | 1.2373% | 4 |
| Flavonoid glycosides | 1.0280% | 60 |
| Fatty acyl thioesters | 0.9000% | 35 |
| Fatty acyl glycosides | 0.6029% | 41 |
| Sugar acids and derivatives | 0.5610% | 5 |
| Isoflav-2-enes | 0.4709% | 5 |
| Carbazoles | 0.4697% | 2 |
| Arylsulfates | 0.4106% | 10 |
| Steroid conjugates | 0.4014% | 23 |
| Benzenesulfonyl compounds | 0.3543% | 2 |
| (3'->5')-cyclic dinucleotides and analogues | 0.3541% | 1 |
| Ceramides | 0.3357% | 8 |
| Glycerophosphoethanolamines | 0.3252% | 20 |
| Linear tetracyclines | 0.3229% | 4 |
| Fatty amides | 0.2523% | 49 |
| Anthracenecarboxylic acids and derivatives | 0.2496% | 4 |
| Carboxylic acid derivatives | 0.2438% | 4 |
| Coumestans | 0.2414% | 6 |
| Oxygenated hydrocarbons | 0.2397% | 22 |
| Glycerophosphates | 0.2267% | 16 |
| Eicosanoids | 0.2233% | 38 |
| Cinnamic acid esters | 0.2146% | 8 |
| 1,4-benzodiazepines | 0.1926% | 6 |
| Aromatic polyketides | 0.1905% | 16 |
| Octadecanoids | 0.1889% | 24 |
| Beta hydroxy acids and derivatives | 0.1745% | 4 |
| Phosphosphingolipids | 0.1523% | 8 |
| Secosteroids | 0.1499% | 20 |
| Bile acids and derivatives | 0.1462% | 13 |
| Monoradylglycerols | 0.1442% | 5 |
| Glycerophosphoglycerophosphates | 0.1329% | 3 |
| Steroids | 0.1310% | 24 |
| Dibenzodiazepines | 0.1160% | 1 |
| Benzoxazolones | 0.1065% | 9 |
| Sesquiterpenoids | 0.1046% | 7 |
| Fatty aldehydes | 0.0954% | 15 |
| Oxidized glycerophospholipids | 0.0924% | 22 |
| Bisphosphonates | 0.0906% | 1 |
| Hydrocarbons | 0.0895% | 12 |
| Hydroxycinnamic acids and derivatives | 0.0877% | 26 |
| Piperazines | 0.0836% | 3 |
| Pyrimidine 2'-deoxyribonucleosides | 0.0759% | 3 |
| Flavones | 0.0720% | 8 |
| Coumarin glycosides | 0.0655% | 5 |
| Beta lactams | 0.0628% | 9 |
| Diarylthioethers | 0.0586% | 7 |
| Biflavonoids and polyflavonoids | 0.0580% | 7 |
| Indoles | 0.0574% | 8 |
| Hydroxycoumarins | 0.0553% | 9 |
| Diterpenoids | 0.0543% | 7 |
| Benzenediols | 0.0510% | 5 |
| Furanocoumarins | 0.0503% | 9 |
| Benzenesulfonamides | 0.0467% | 11 |
| Neutral glycosphingolipids | 0.0457% | 7 |
| Thiophosphoric acid esters | 0.0422% | 2 |
| Pyrimidines and pyrimidine derivatives | 0.0420% | 5 |
| Terpene glycosides | 0.0417% | 11 |
| Substituted pyrroles | 0.0355% | 1 |
| Tetracarboxylic acids and derivatives | 0.0348% | 6 |
| Chlorohydrins | 0.0346% | 1 |
| Other acyl sugars | 0.0329% | 3 |
| Phenylnaphthalenes | 0.0325% | 6 |
| Benzodiazines | 0.0312% | 5 |
| Tricarboxylic acids and derivatives | 0.0302% | 11 |
| Dicarboxylic acids and derivatives | 0.0294% | 5 |
| Hydroxyindoles | 0.0290% | 2 |
| Fatty acid esters | 0.0290% | 11 |
| Isoflavonoid O-glycosides | 0.0280% | 3 |
| Chalcones and dihydrochalcones | 0.0267% | 6 |
| Purines and purine derivatives | 0.0263% | 11 |
| Ergostane steroids | 0.0260% | 1 |
| Cinnamic acids | 0.0259% | 1 |
| Macrolides and lactone polyketides | 0.0251% | 4 |
| Terpene lactones | 0.0250% | 6 |
| Quinone and hydroquinone lipids | 0.0247% | 4 |
| Quinoline carboxylic acids | 0.0202% | 5 |
| Anisoles | 0.0202% | 7 |
| Cytochalasins | 0.0192% | 1 |
| Aminotriazines | 0.0187% | 1 |
| Monosaccharides | 0.0183% | 4 |
| Flavans | 0.0176% | 6 |
| Pyrazoles | 0.0175% | 8 |
| Methoxyphenols | 0.0158% | 6 |
| Steroid esters | 0.0157% | 4 |
| Diphenylethers | 0.0153% | 1 |
| O-methylated flavonoids | 0.0150% | 5 |
| Sulfonic acids | 0.0147% | 1 |
| Indolyl carboxylic acids and derivatives | 0.0141% | 2 |
| Organoarsenic compounds | 0.0139% | 1 |
| Pterins and derivatives | 0.0137% | 3 |
| Carbonyl compounds | 0.0135% | 9 |
| Diphenylacetonitriles | 0.0134% | 1 |
| Tetrahydrofuran lignans | 0.0126% | 1 |
| Linear diarylheptanoids | 0.0123% | 5 |
| Monoterpenoids | 0.0123% | 3 |
| Pyrimidine nucleotide sugars | 0.0120% | 4 |
| Androstane steroids | 0.0120% | 3 |
| Thiazoles | 0.0118% | 2 |
| Estrane steroids | 0.0116% | 4 |
| Polyether antibiotics | 0.0111% | 1 |
| Medium-chain keto acids and derivatives | 0.0107% | 3 |
| Purine ribonucleotides | 0.0107% | 3 |
| Glycosyl compounds | 0.0103% | 5 |
| Pyridinecarboxylic acids and derivatives | 0.0103% | 2 |
| Triterpenoids | 0.0102% | 4 |
| Hydropyridines | 0.0101% | 4 |
| Phosphate esters | 0.0091% | 3 |
| Other Fatty Acyls | 0.0090% | 2 |
| Glycosylglycerols | 0.0090% | 1 |
| Phenylpyruvic acid derivatives | 0.0089% | 3 |
| Medium-chain hydroxy acids and derivatives | 0.0087% | 2 |
| Sulfuric acid esters | 0.0087% | 3 |
| Indolecarboxylic acids and derivatives | 0.0084% | 3 |
| Cyclic purine nucleotides | 0.0083% | 4 |
| Tryptamines and derivatives | 0.0082% | 4 |
| Phenols and derivatives | 0.0080% | 1 |
| Benzylisoquinolines | 0.0080% | 5 |
| Phenolic lipids | 0.0080% | 3 |
| Docosanoids | 0.0080% | 4 |
| Bipyridines and oligopyridines | 0.0076% | 3 |
| Naphthalene sulfonic acids and derivatives | 0.0073% | 2 |
| Organosulfonic acids and derivatives | 0.0073% | 2 |
| Nitrophenols | 0.0072% | 1 |
| Pyridoindoles | 0.0071% | 4 |
| Cyclic diarylheptanoids | 0.0067% | 2 |
| Stilbene glycosides | 0.0065% | 2 |
| Bile acids, alcohols and derivatives | 0.0063% | 3 |
| Ketones | 0.0062% | 1 |
| Glycosyldiradylglycerols | 0.0062% | 1 |
| Indazoles | 0.0060% | 3 |
| Hybrid peptides | 0.0059% | 1 |
| Trifluoromethylbenzenes | 0.0056% | 1 |
| Physalins and derivatives | 0.0054% | 2 |
| Phenylbutylamines | 0.0054% | 1 |
| Prenylated neoflavonoids | 0.0053% | 2 |
| Phenylacetic acids | 0.0053% | 1 |
| Steroid lactones | 0.0050% | 3 |
| Phenylquinolines | 0.0049% | 1 |
| Quinolones and derivatives | 0.0049% | 2 |
| Phenylmethylamines | 0.0049% | 2 |
| Toluenes | 0.0047% | 1 |
| Pyranoisoflavonoids | 0.0046% | 1 |
| Pyranones and derivatives | 0.0046% | 2 |
| Glycerophosphoinositolglycans | 0.0046% | 2 |
| Hydrophenanthrenes | 0.0045% | 1 |
| Isoflavans | 0.0044% | 2 |
| Gamma butyrolactones | 0.0043% | 2 |
| Cytochalasins | 0.0041% | 1 |
| Gamma-keto acids and derivatives | 0.0041% | 3 |
| Thioesters | 0.0040% | 2 |
| Glycosphingolipids | 0.0037% | 2 |
| N-phenylureas | 0.0036% | 1 |
| Pyrrolidinylpyridines | 0.0035% | 2 |
| Cyclamates | 0.0035% | 2 |
| Benzyl alcohols | 0.0035% | 1 |
| (3'->5')-dinucleotides | 0.0034% | 1 |
| Dibenzazepines | 0.0034% | 1 |
| Sulfones | 0.0033% | 1 |
| Phenylpropenes | 0.0033% | 4 |
| Triradylglycerols | 0.0032% | 2 |
| CDP-glycerols | 0.0032% | 1 |
| Phenoxyacetic acid derivatives | 0.0031% | 1 |
| Diphenylmethanes | 0.0031% | 2 |
| Betacyanins and derivatives | 0.0031% | 1 |
| Polyprenols | 0.0030% | 1 |
| Furanoisoflavonoids | 0.0028% | 2 |
| Pyranoflavonoids | 0.0028% | 1 |
| Aniline and substituted anilines | 0.0027% | 2 |
| Imidazolidines | 0.0026% | 1 |
| Isoprenoid phosphates | 0.0024% | 1 |
| Phenothiazines | 0.0022% | 1 |
| Sulfinylbenzimidazoles | 0.0022% | 1 |
| Naphthopyranones | 0.0021% | 1 |
| Branched unsaturated hydrocarbons | 0.0021% | 1 |
| Hydroxyisoflavonoids | 0.0020% | 1 |
| Pregnane steroids | 0.0019% | 1 |
| Benzoyl derivatives | 0.0019% | 1 |
| Other Glycerophospholipids | 0.0019% | 1 |
| Naphthyridines | 0.0018% | 1 |
| Pyrimidine 2',3'-dideoxyribonucleosides | 0.0017% | 1 |
| Cycloartanols and derivatives | 0.0016% | 1 |
| Lysergic acids and derivatives | 0.0016% | 1 |
| Anilides | 0.0016% | 1 |
| Halobenzenes | 0.0016% | 1 |
| 1,4-dioxepanes | 0.0016% | 1 |
| Porphyrins | 0.0016% | 1 |
| Secobenzophenanthridine alkaloids | 0.0015% | 1 |
| Benzopyrenes | 0.0015% | 1 |
| Benzylethers | 0.0015% | 1 |
| Pyrimidine deoxyribonucleotides | 0.0015% | 1 |
| Cycloalkanes | 0.0014% | 1 |
| Angucyclines | 0.0014% | 1 |
| Phenylacetaldehydes | 0.0014% | 1 |
| Furanones | 0.0014% | 1 |
| Primary amines | 0.0014% | 1 |
| Dithioacetals | 0.0013% | 1 |
| Indolines | 0.0013% | 1 |
| Cumenes | 0.0011% | 1 |
| Quaternary ammonium salts | 0.0011% | 1 |
| Phenylpiperidines | 0.0011% | 1 |
| Triazoles | 0.0011% | 1 |
| Basic glycosphingolipids | 0.0010% | 1 |
| Pyrrolo[2,3-d]pyrimidines | 0.0010% | 1 |
| Piperidinones | 0.0010% | 2 |
| Tetrazoles | 0.0009% | 1 |
| Benzenesulfonic acids and derivatives | 0.0009% | 1 |
| Diazanaphthalenes | 0.0008% | 1 |
| Ansamycins and related polyketides | 0.0008% | 1 |
| Dibenzoxepines | 0.0008% | 1 |
| Imidazoles | 0.0008% | 1 |
| Pyrroloindoles | 0.0007% | 1 |
| Tertiary amines | 0.0007% | 1 |
| Phenethylamines | 0.0007% | 1 |
| Phenylpyridines | 0.0007% | 1 |
| Morpholines | 0.0006% | 1 |
| Ethers | 0.0005% | 1 |
| Benzaldehydes | 0.0005% | 1 |
| Oxazoles | 0.0005% | 1 |
| Biphenyls and derivatives | 0.0005% | 1 |
| O-methylated isoflavonoids | 0.0004% | 1 |
| Styrenes | 0.0004% | 1 |
| Hydroquinolines | 0.0004% | 1 |
| Secondary alcohols | 0.0002% | 1 |
