# Supplementary material for: Extract of Phyllanthus emblica L. fruit stimulates basal glucose uptake and ameliorates palmitate-induced insulin resistance through AMPK activation in C2C12 myotubes
Source: BMC Complement Med Ther. 2024 Aug 2;24:296. doi: 10.1186/s12906-024-04592-1 (PMC11295889; doi:10.1186/s12906-024-04592-1)
Supplement: Supplementary file 4 — Supplementary Material 4 [file 12906_2024_4592_MOESM4_ESM.docx]

Supplementary Figure S2


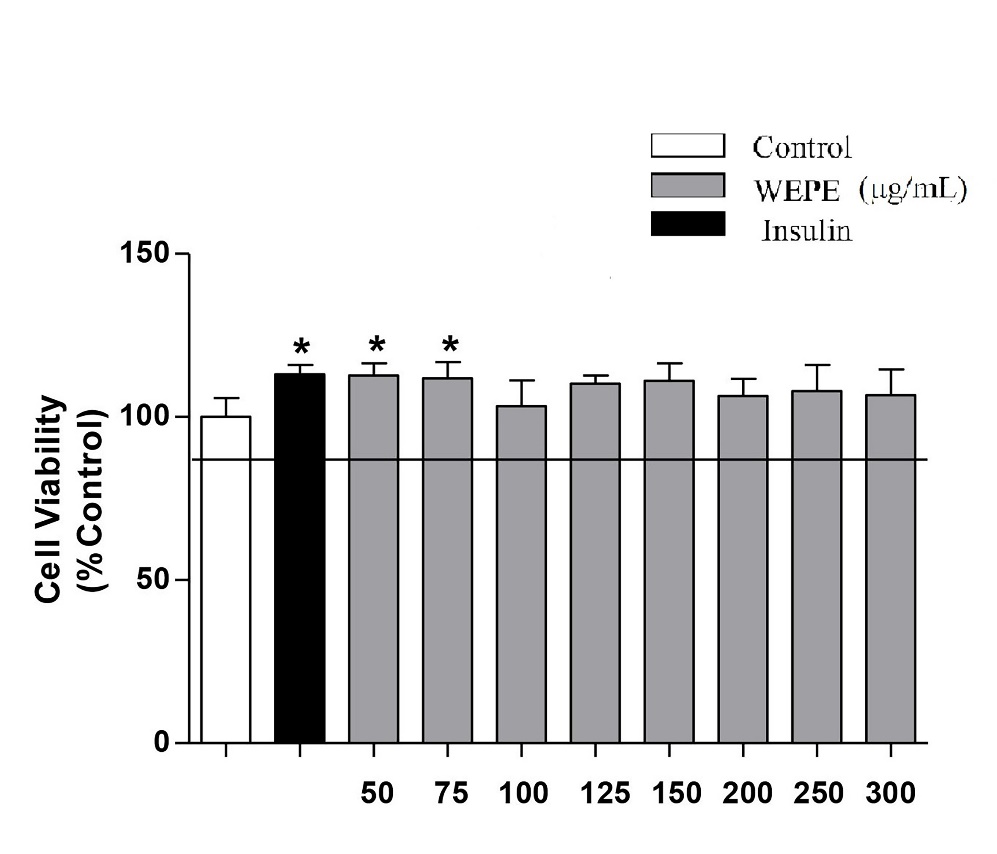


Figure S2. Effects of WEPE on cell viability. Cells were treated with WEPE at dose of 50, 75, 100, 125, 150, 200, 250 or 300 μg/mL for 48 h, and then cell viability were determined using MTT assay (n=10).
