# Supplementary material for: Extract of Phyllanthus emblica L. fruit stimulates basal glucose uptake and ameliorates palmitate-induced insulin resistance through AMPK activation in C2C12 myotubes
Source: BMC Complement Med Ther. 2024 Aug 2;24:296. doi: 10.1186/s12906-024-04592-1 (PMC11295889; doi:10.1186/s12906-024-04592-1)
Supplement: Supplementary file 5 — Supplementary Material 5 [file 12906_2024_4592_MOESM5_ESM.docx]

Supplementary Figure S3


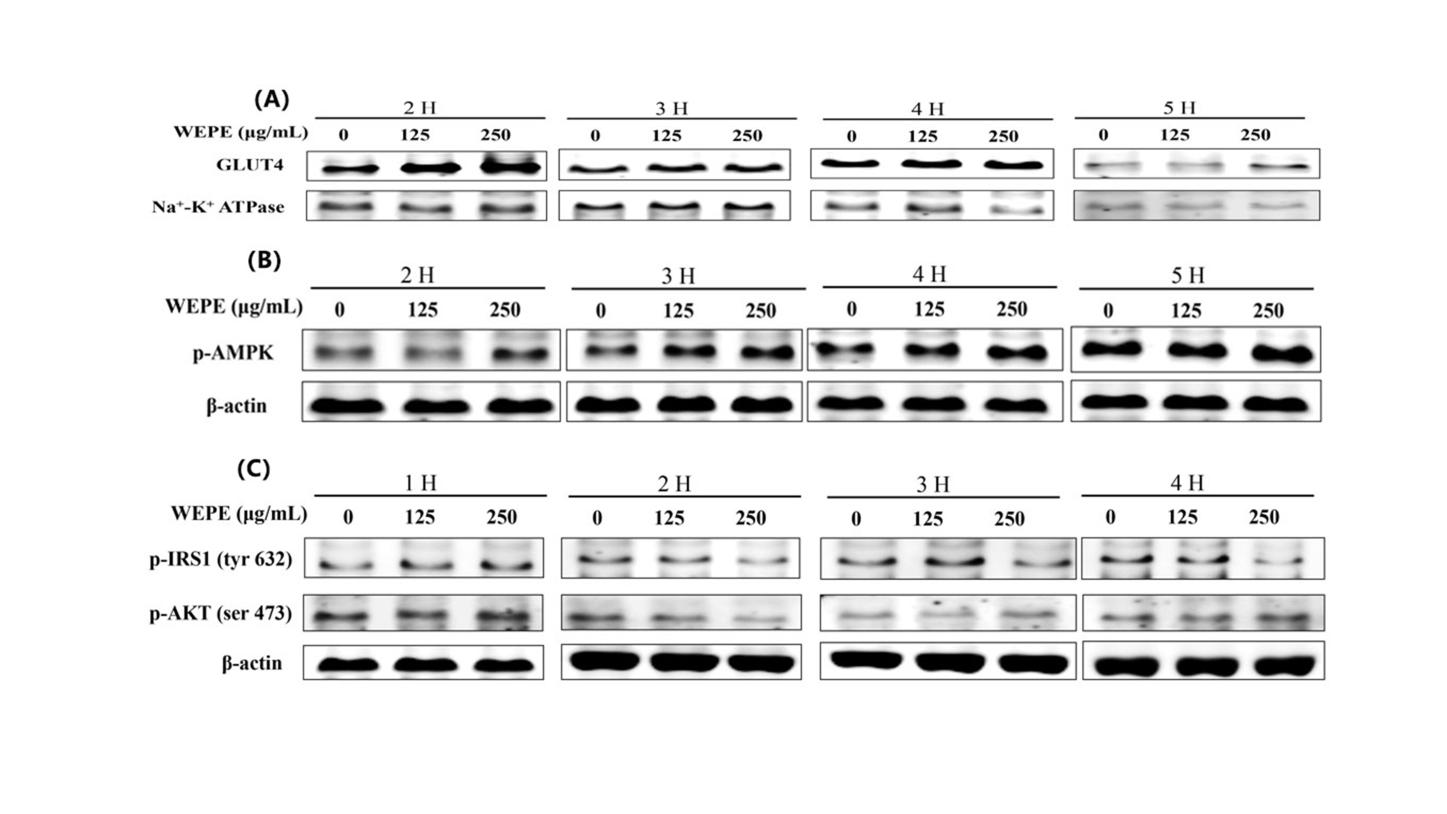


Figure S3. Effects of WEPE on proteins involved in insulin and AMPK signaling pathways as shown by western blotting of whole cell lysates from C2C12 cells incubated with WEPE (125 and 250 μg/mL) for different time. Translocation levels of GLUT4 and phosphorylation of three proteins were examined: AMPK, IRS1 and AKT. Na^+^-K^+^ ATPase and β-actin were used as standards.
