# Supplementary material for: Extract of Phyllanthus emblica L. fruit stimulates basal glucose uptake and ameliorates palmitate-induced insulin resistance through AMPK activation in C2C12 myotubes
Source: BMC Complement Med Ther. 2024 Aug 2;24:296. doi: 10.1186/s12906-024-04592-1 (PMC11295889; doi:10.1186/s12906-024-04592-1)
Supplement: Supplementary file 6 — Supplementary Material 6 [file 12906_2024_4592_MOESM6_ESM.pdf]

Original western blot images of Figure 1-7

Figure 1C GLUT4/Na$^+$-K$^+$ATPase

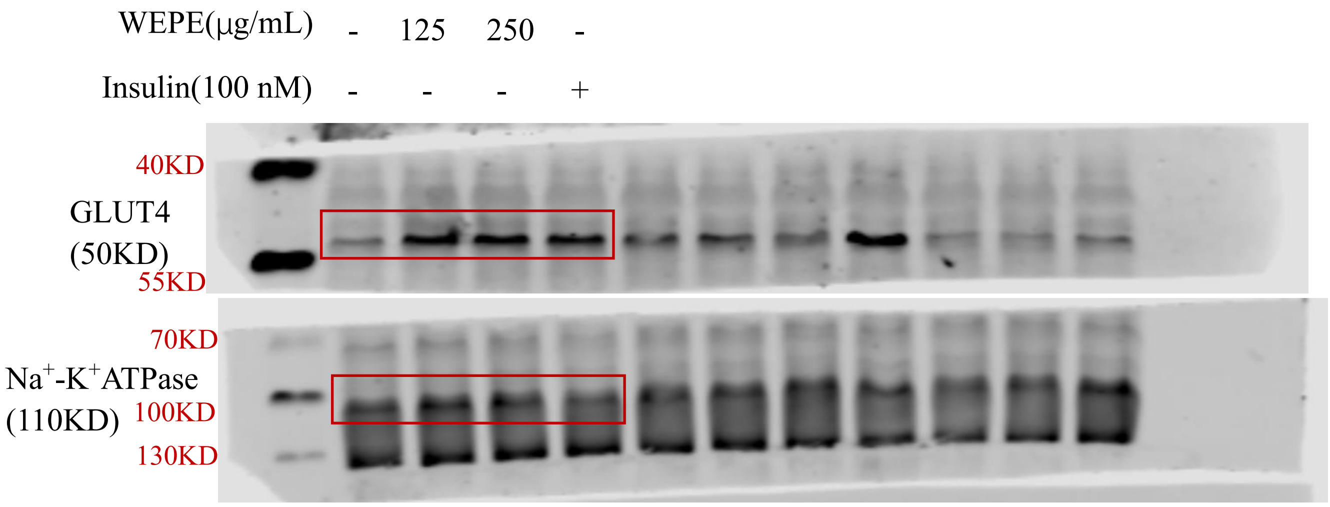

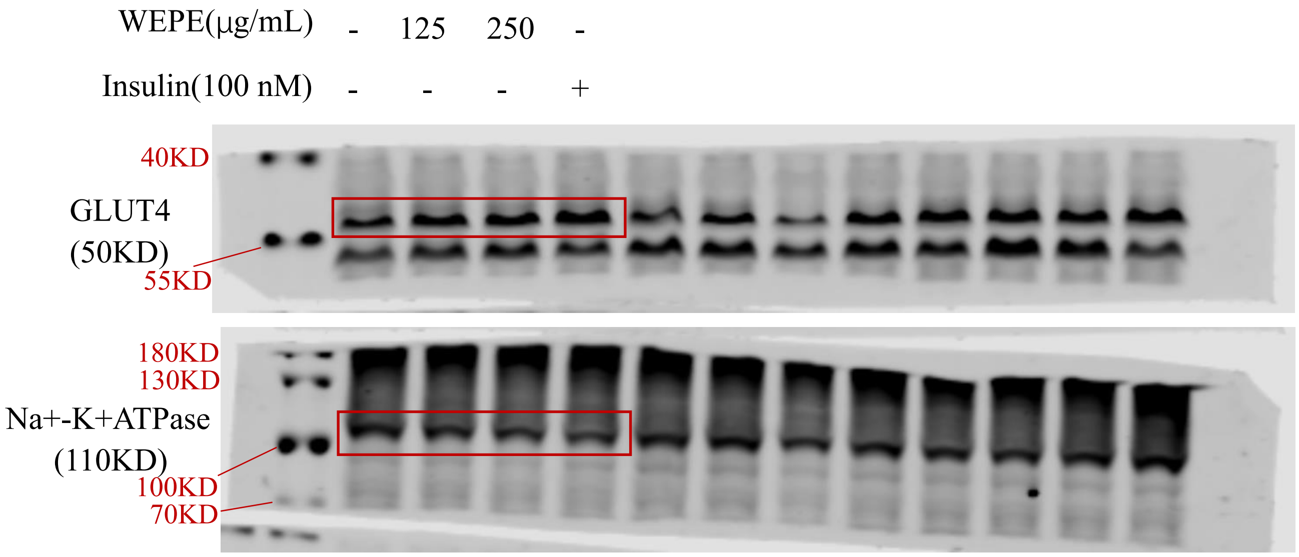

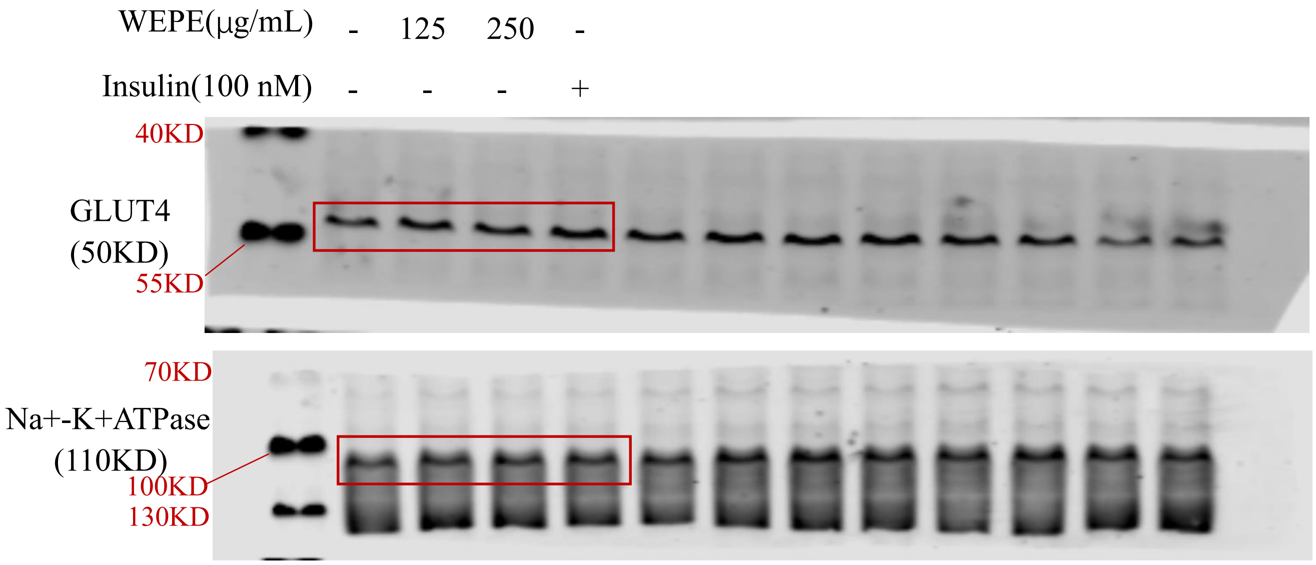

Figure 2A p-AMPK/AMPK

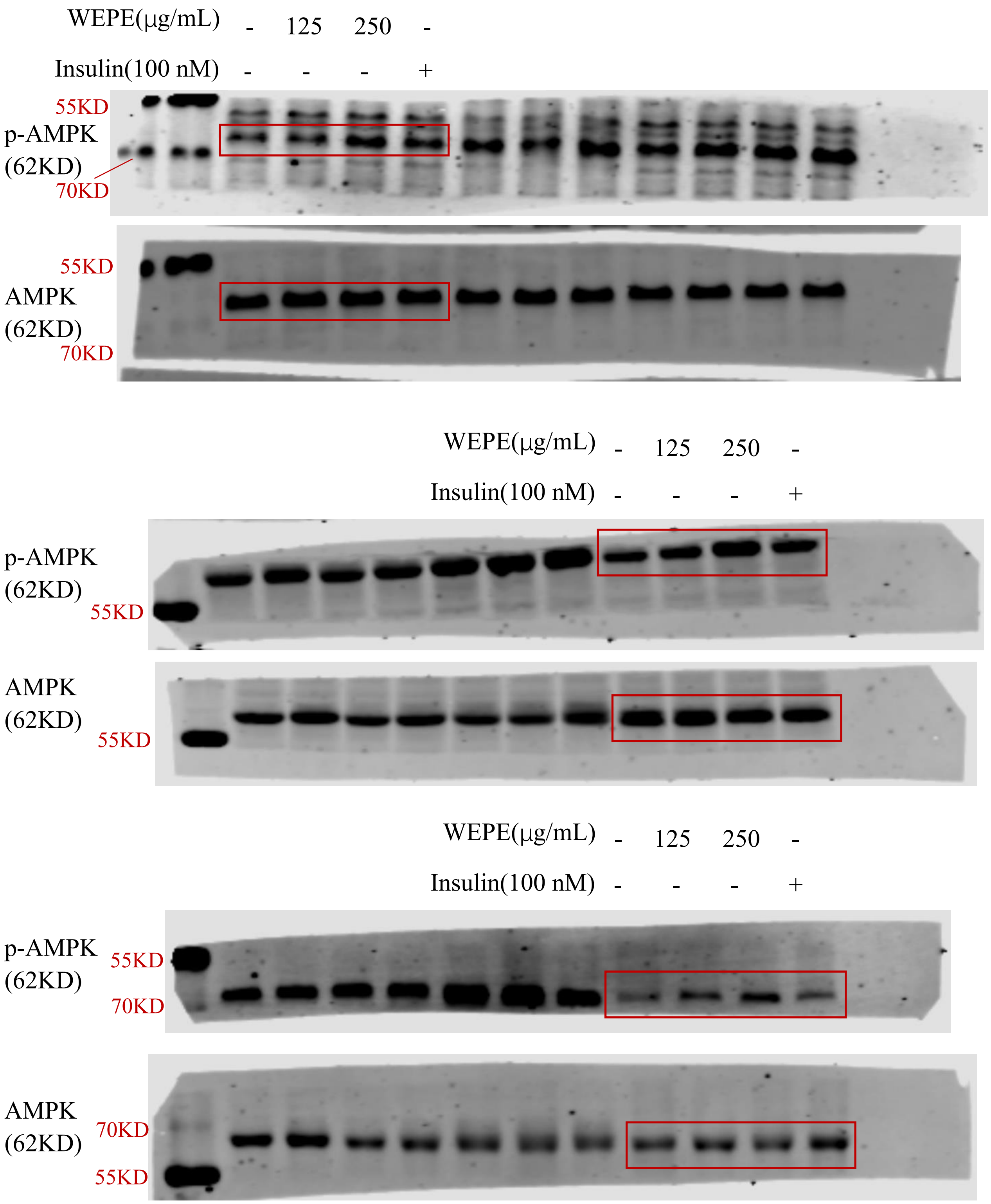

Figure 2A   p-ACC/ACC

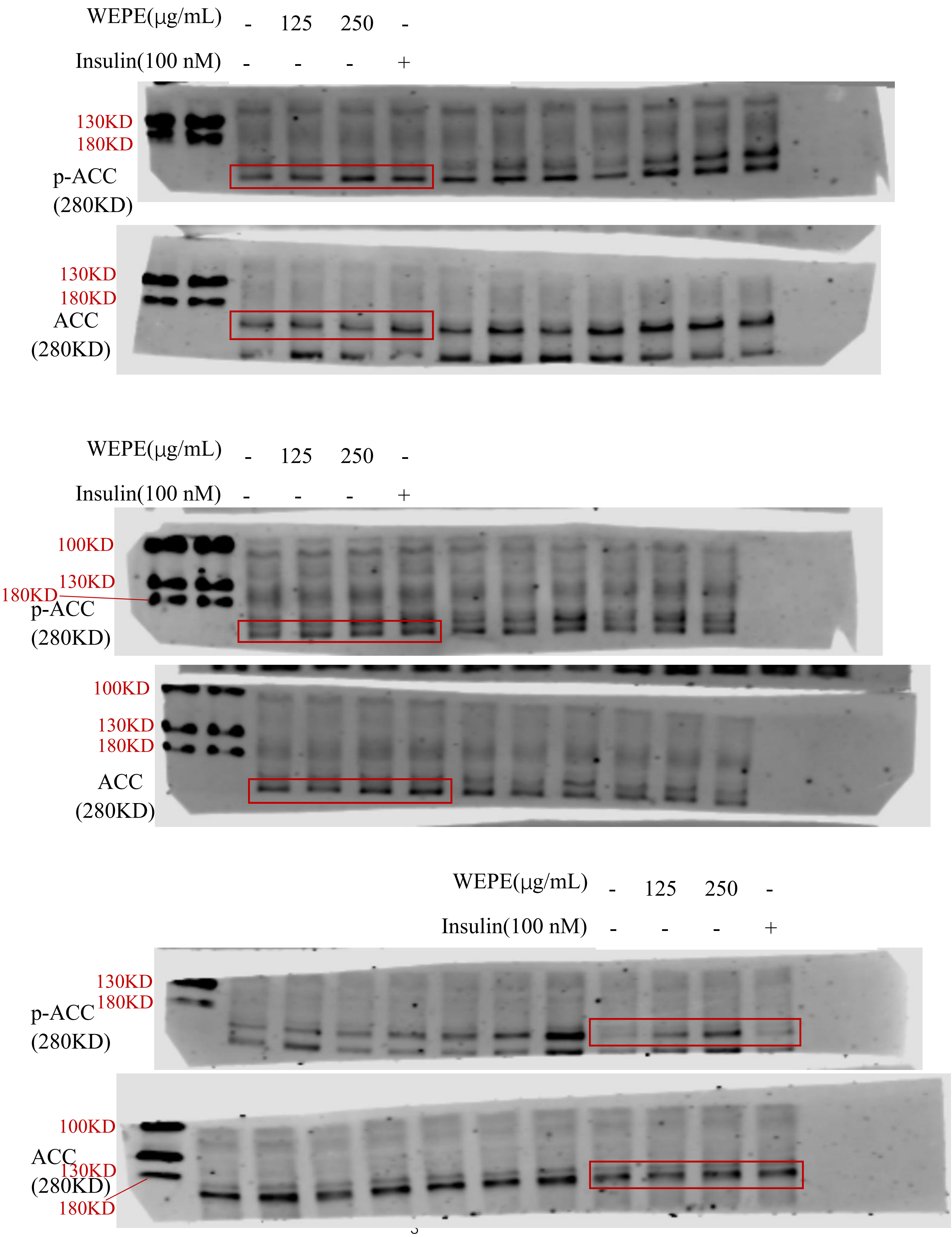

Figure 2A p-AS160/β-actin

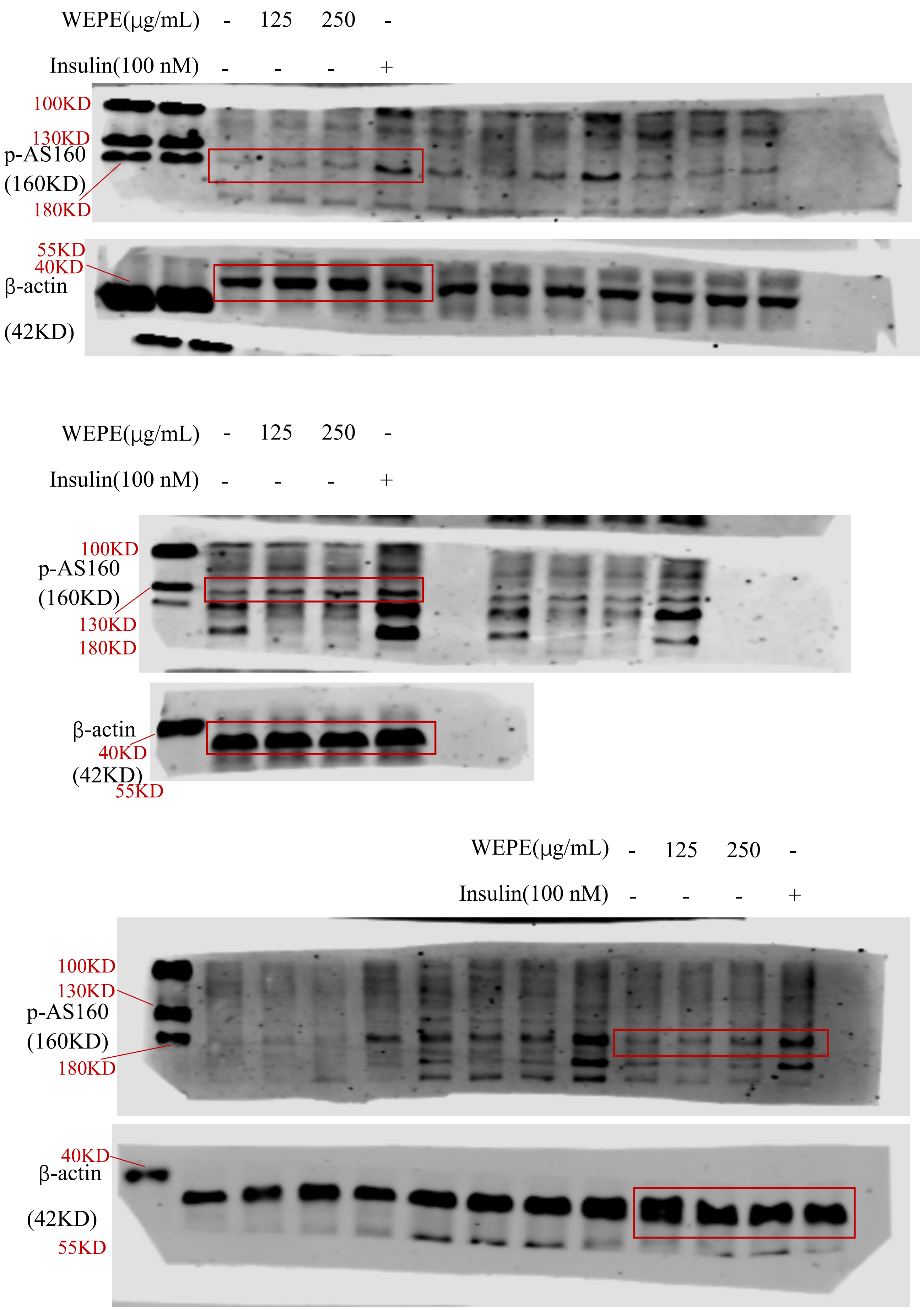

Figure 2A p-p38 MAPK/p38 MAPK

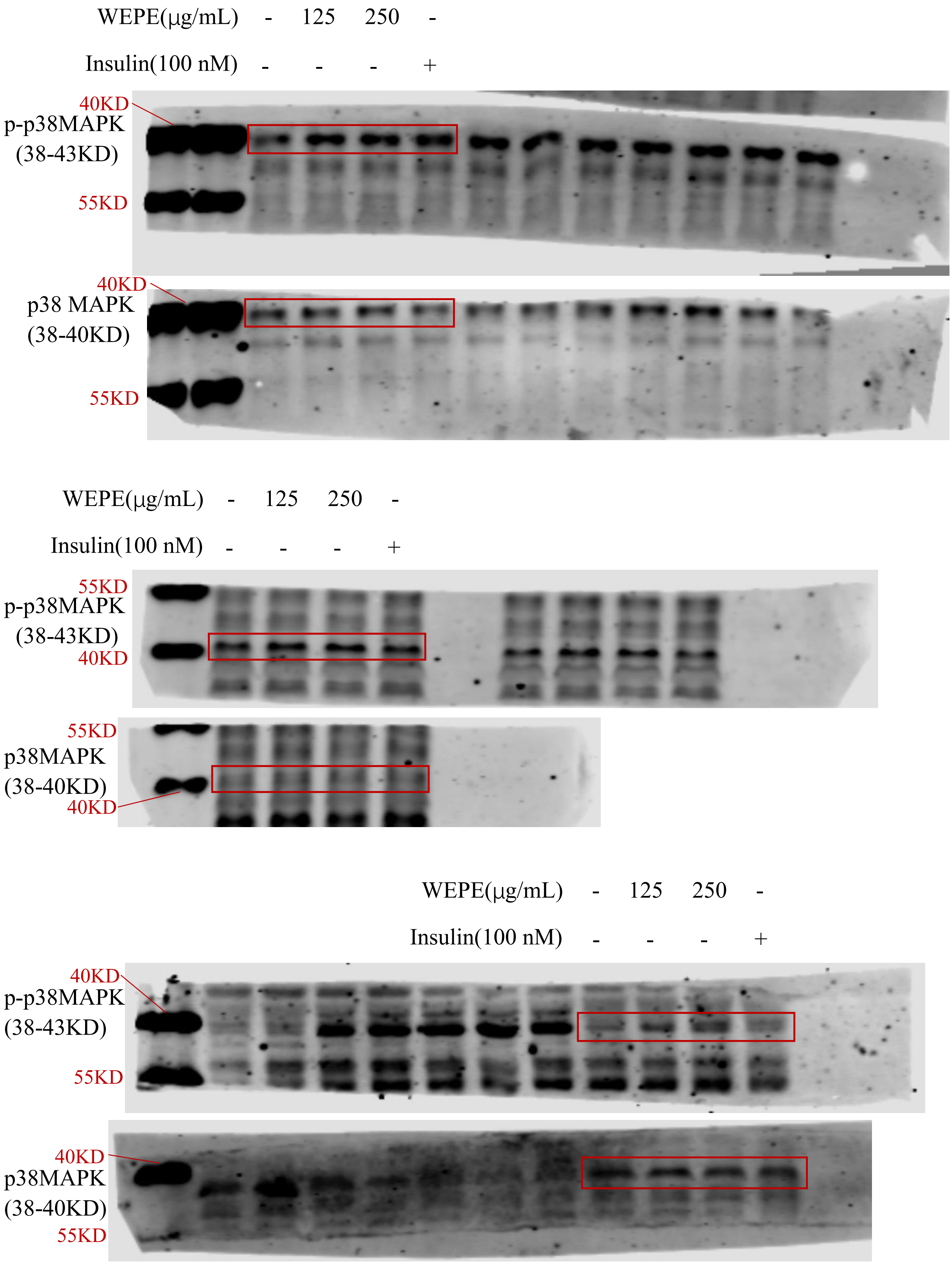

Figure 3A   p-AMPK/AMPK

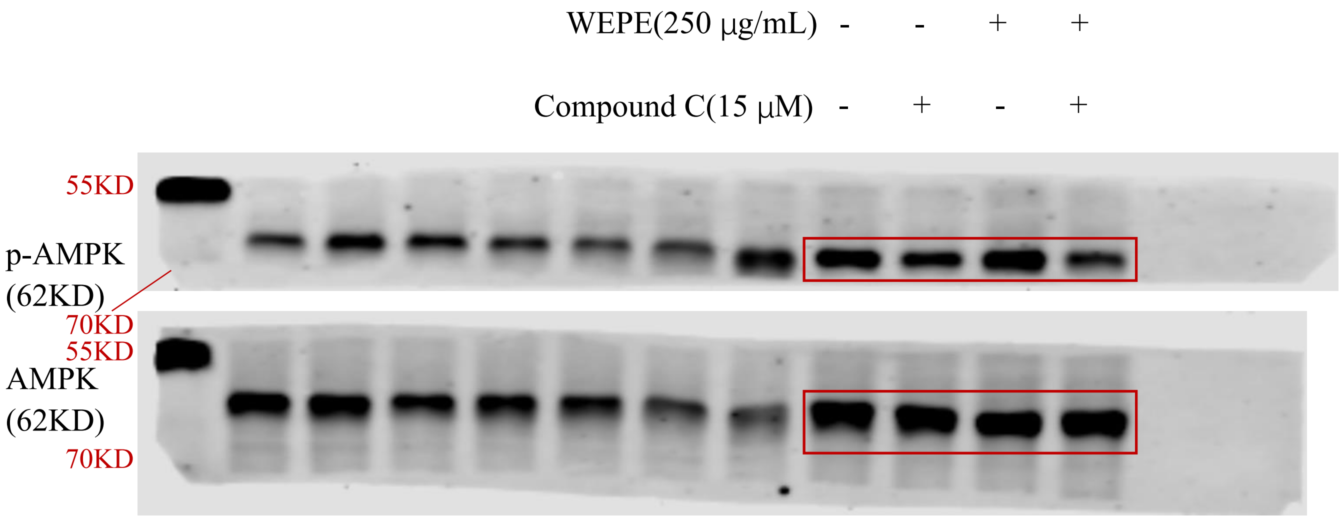

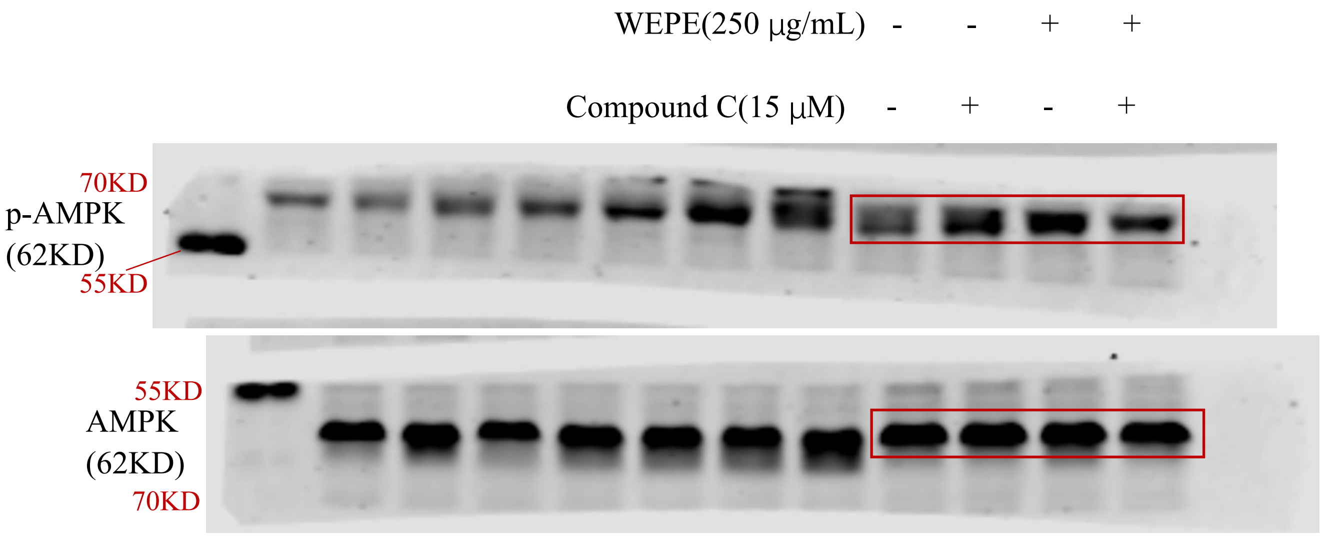

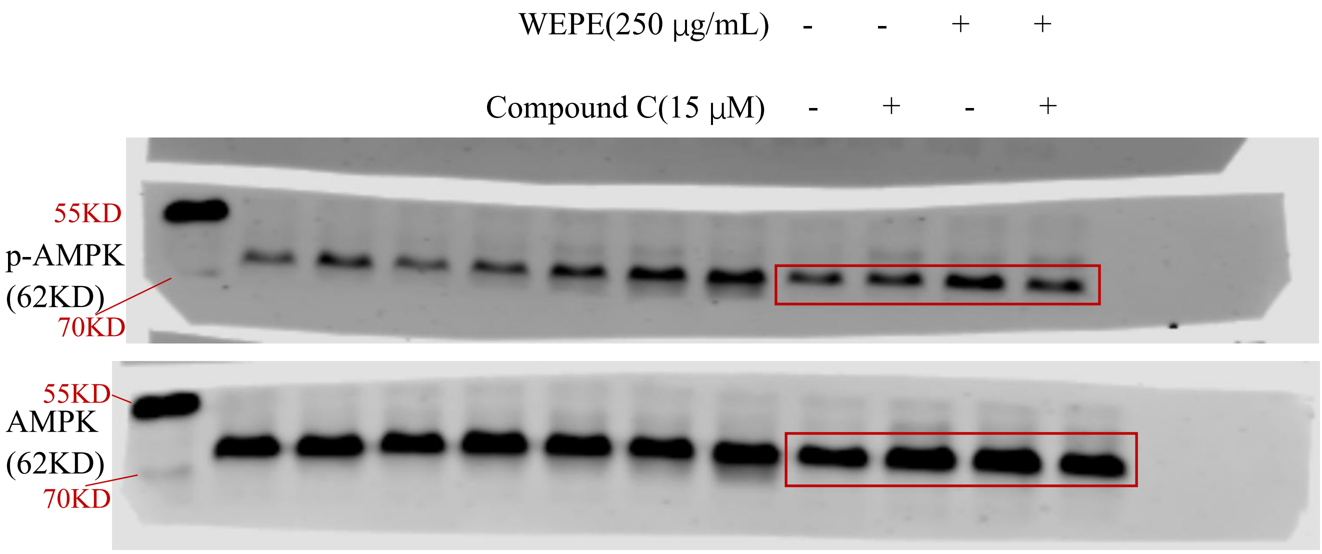

Figure 3A p-ACC/ACC

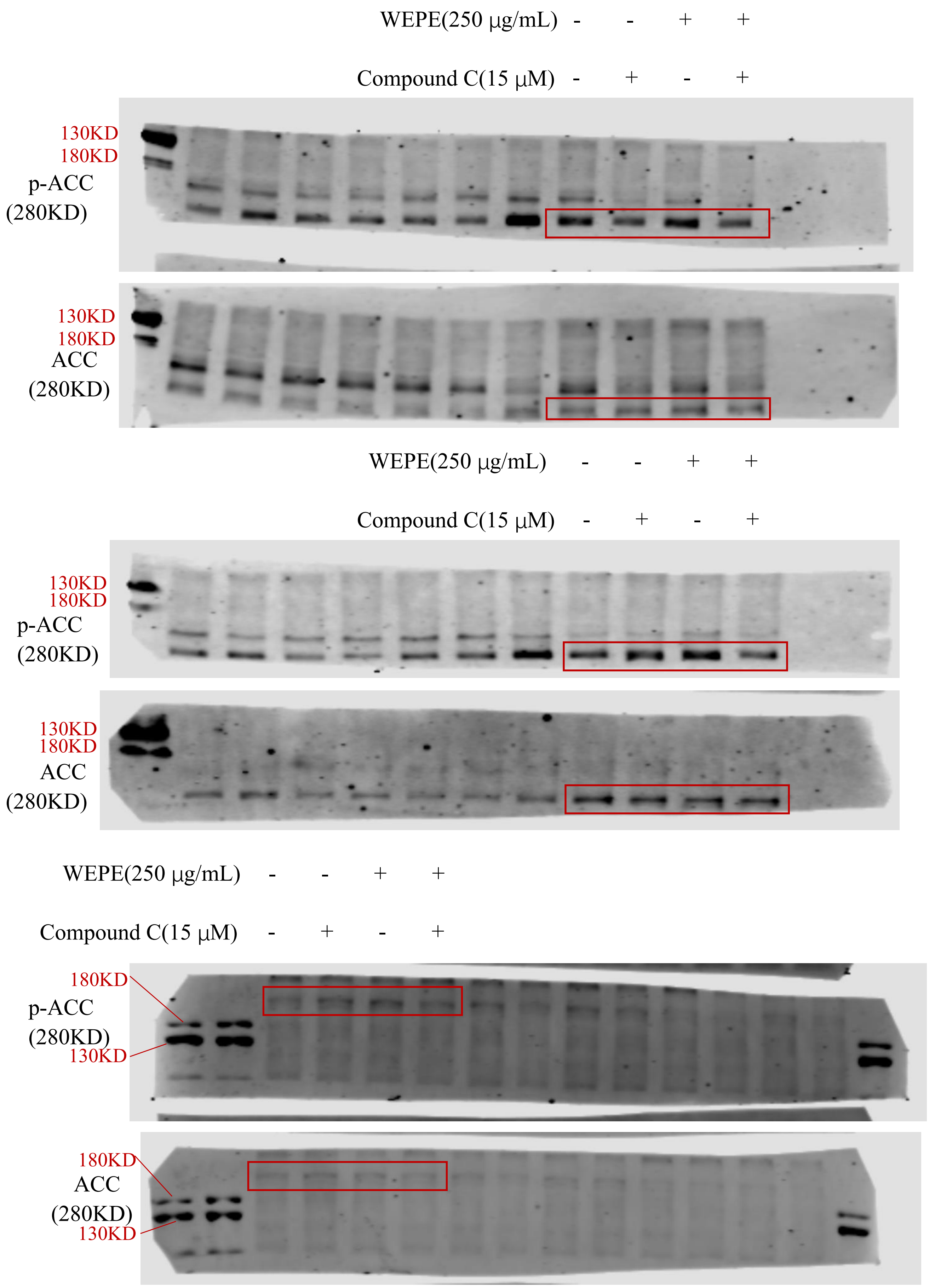

Figure 3A p-AS160/β-actin

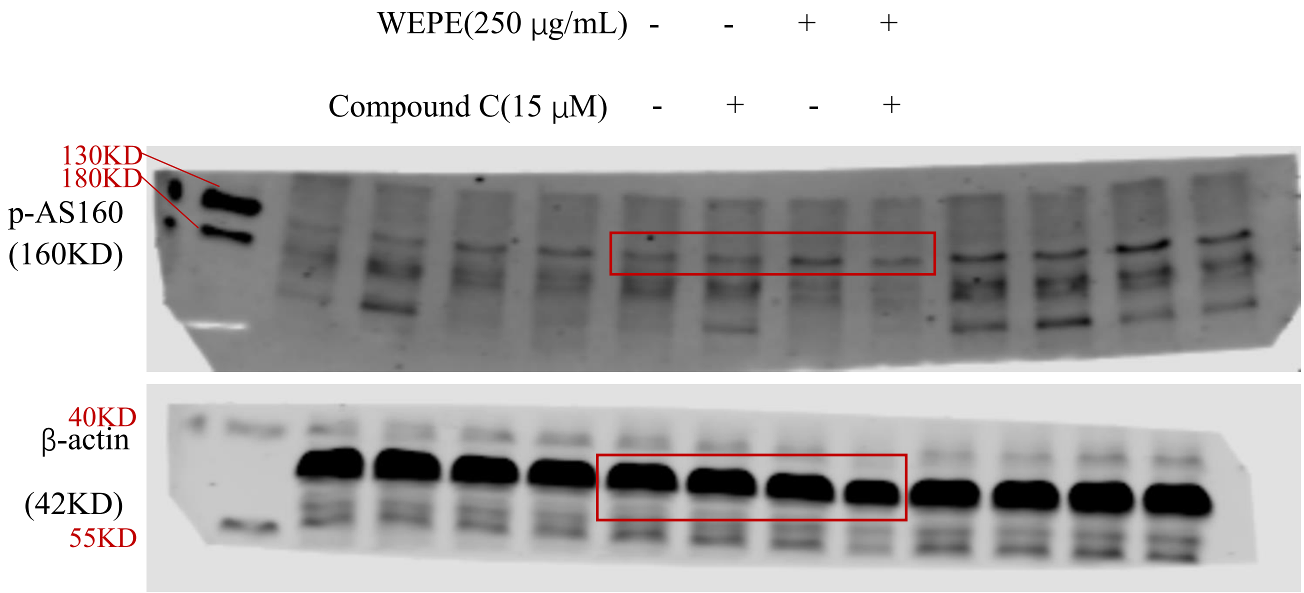

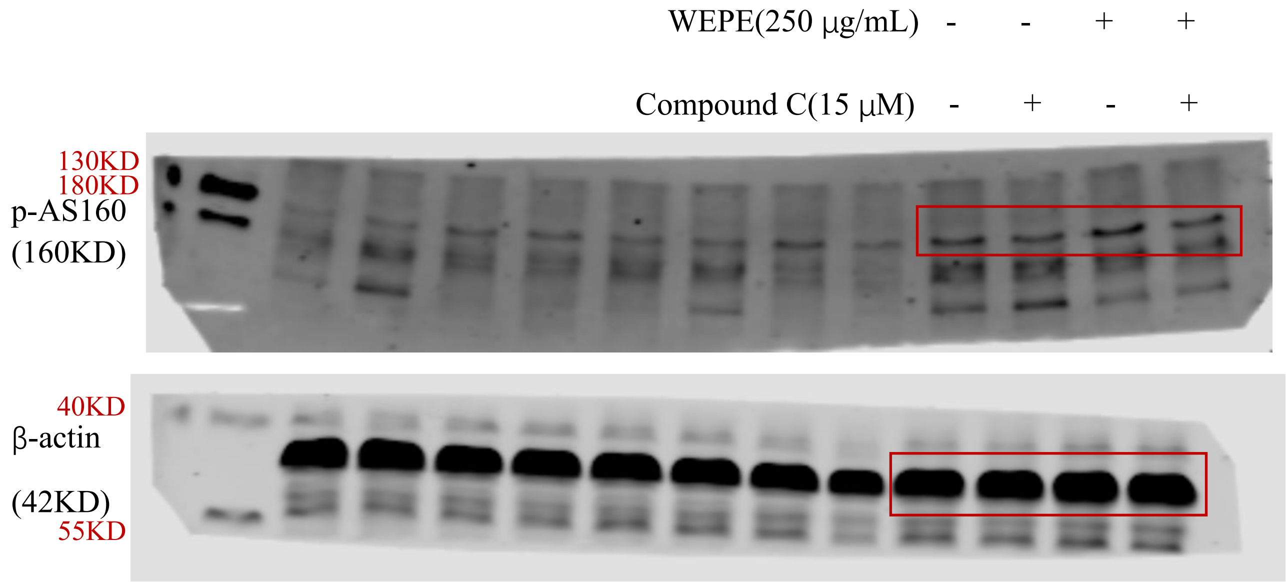

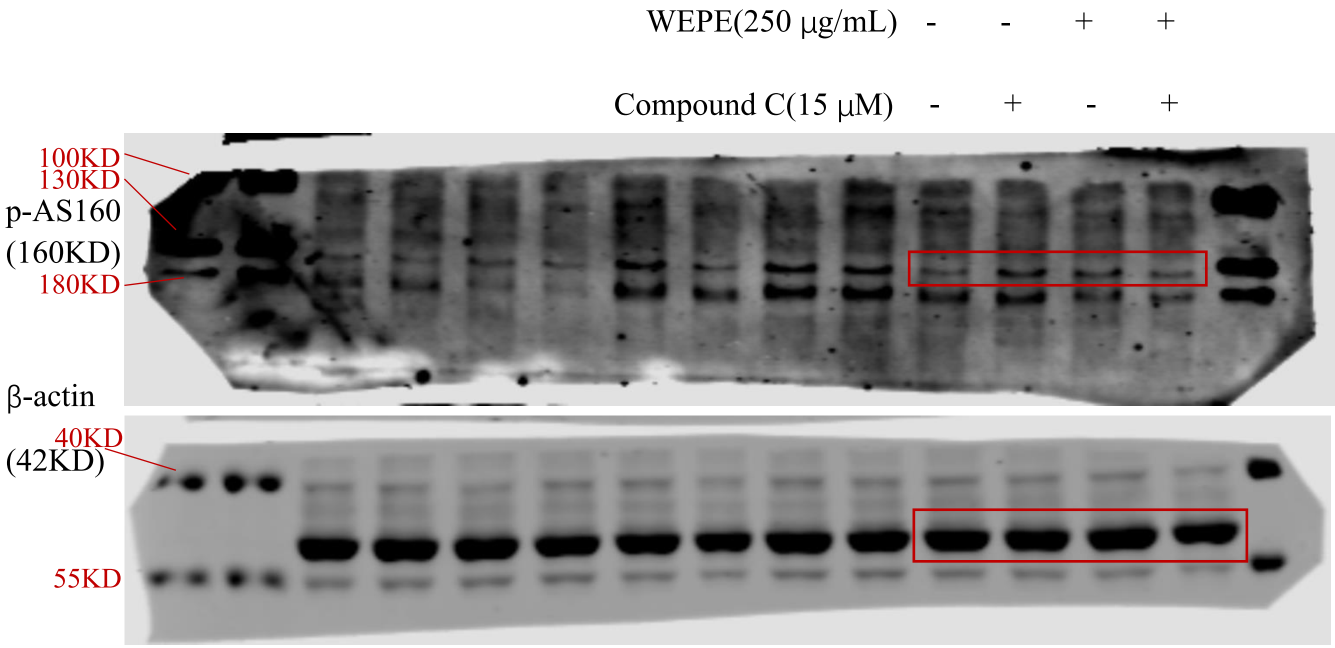

Figure 3A p-p38MAPK/p38MAPK

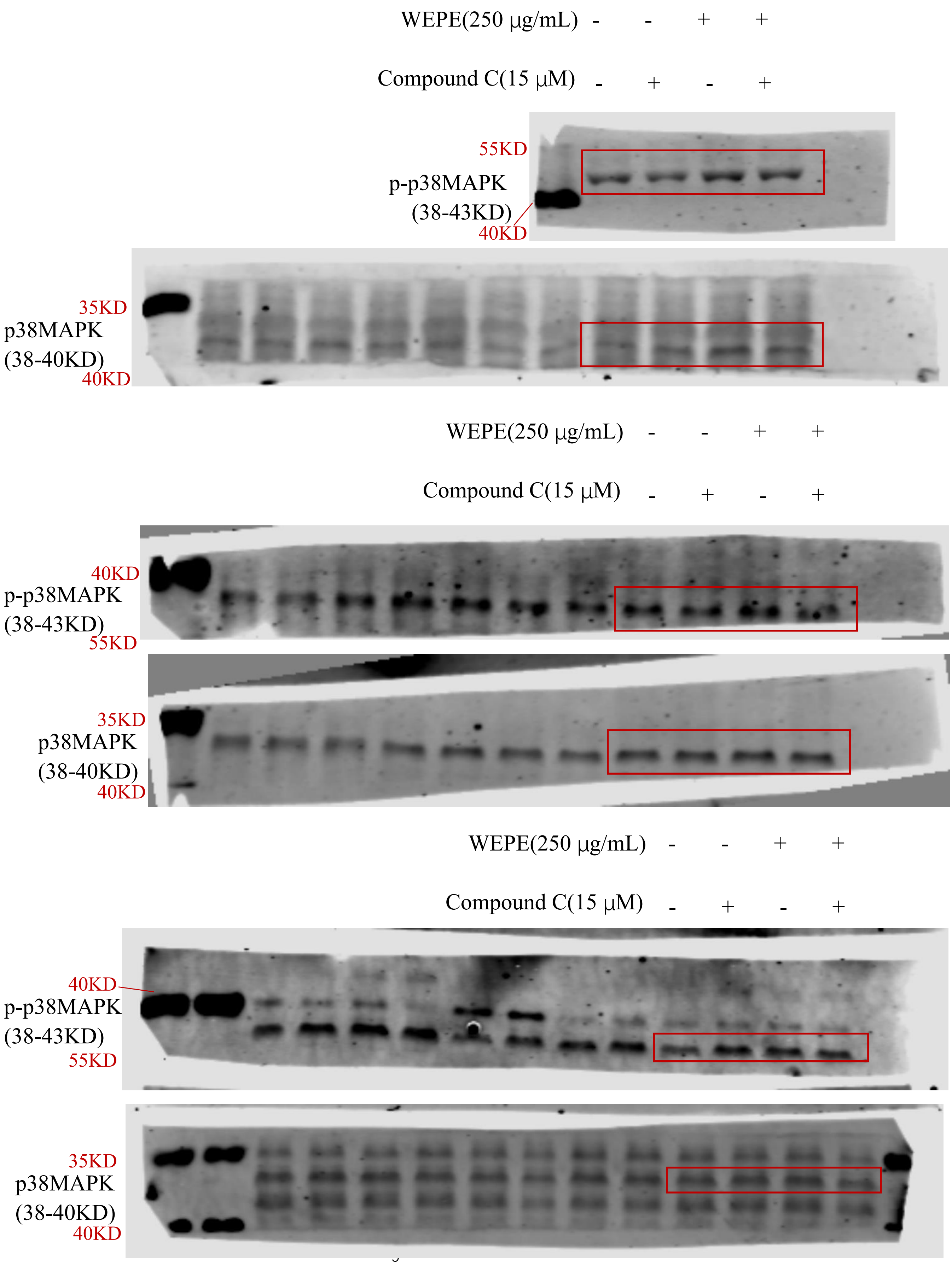

Figure 3A GLUT4/Na^{+}-K^{+}ATPase

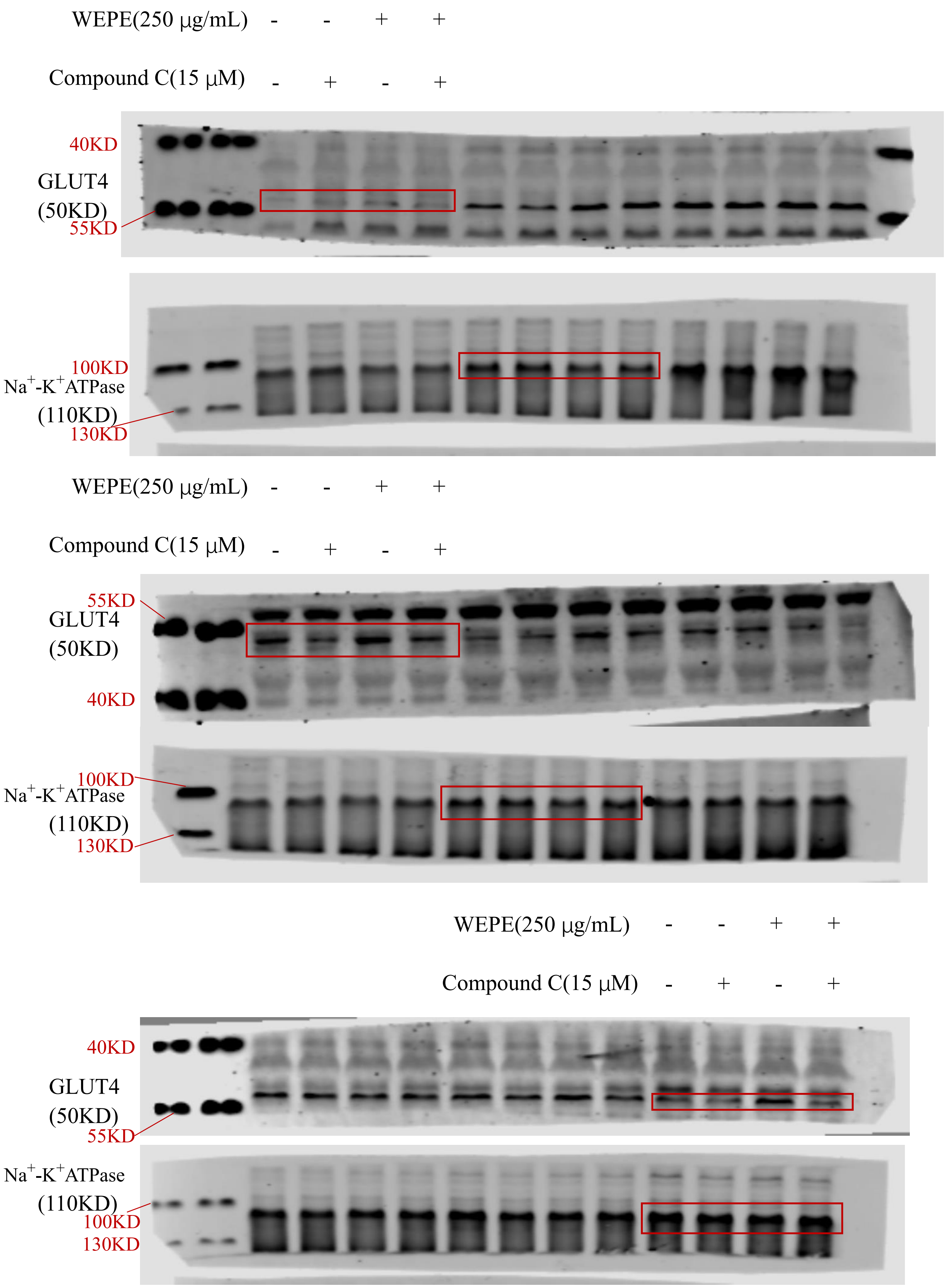

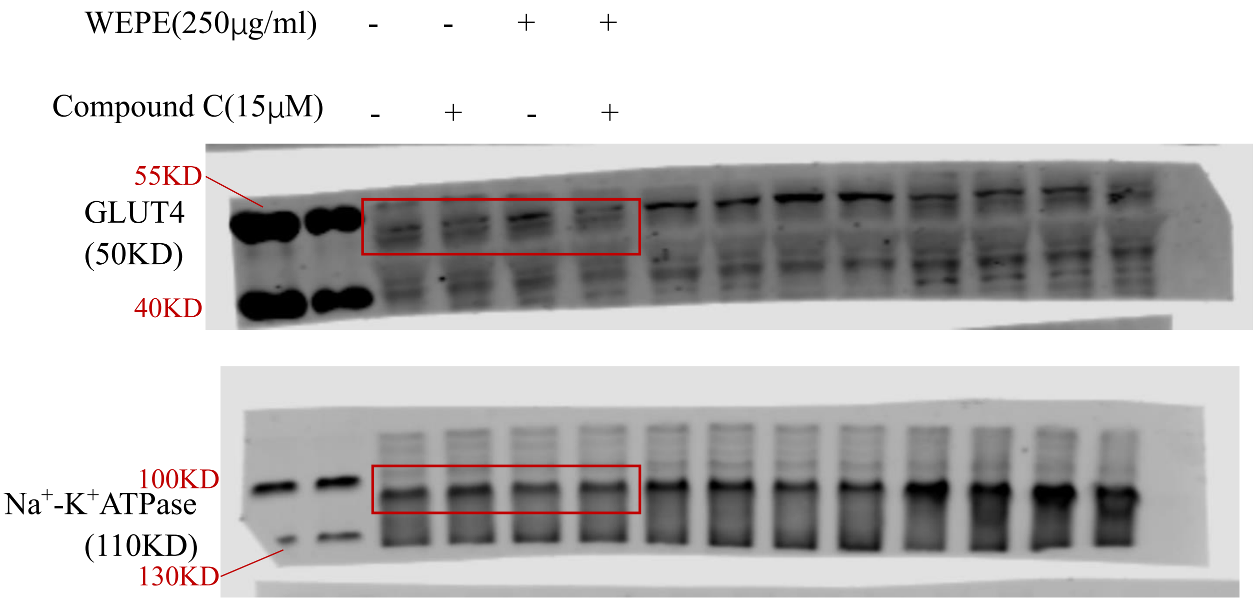

Figure 4A AMPK

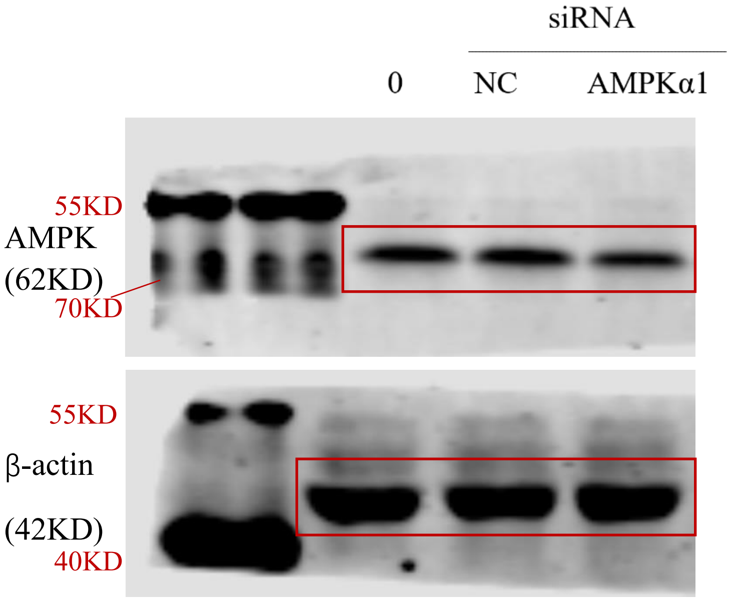

Figure 4A p-AMPK/AMPK/β-actin

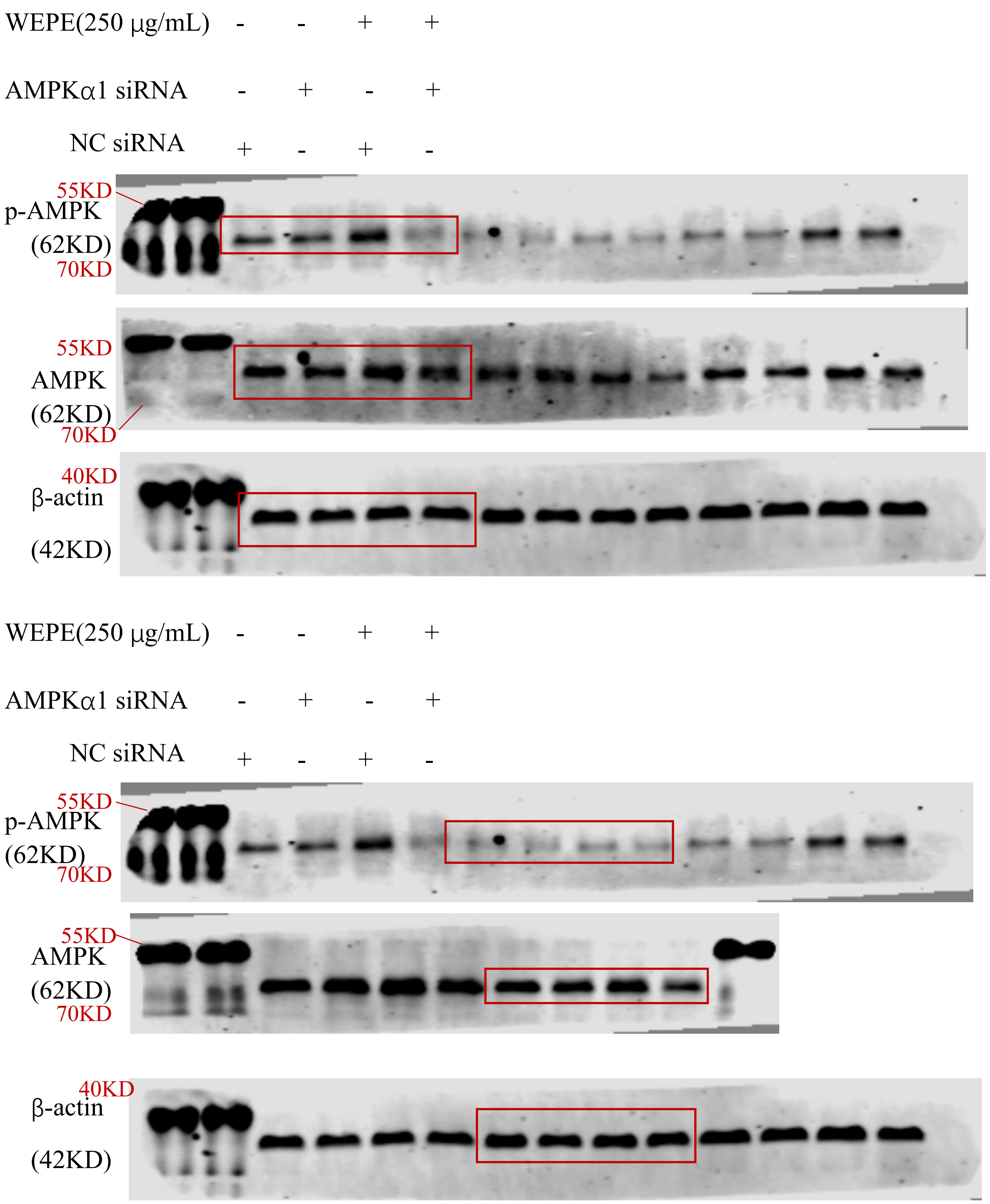

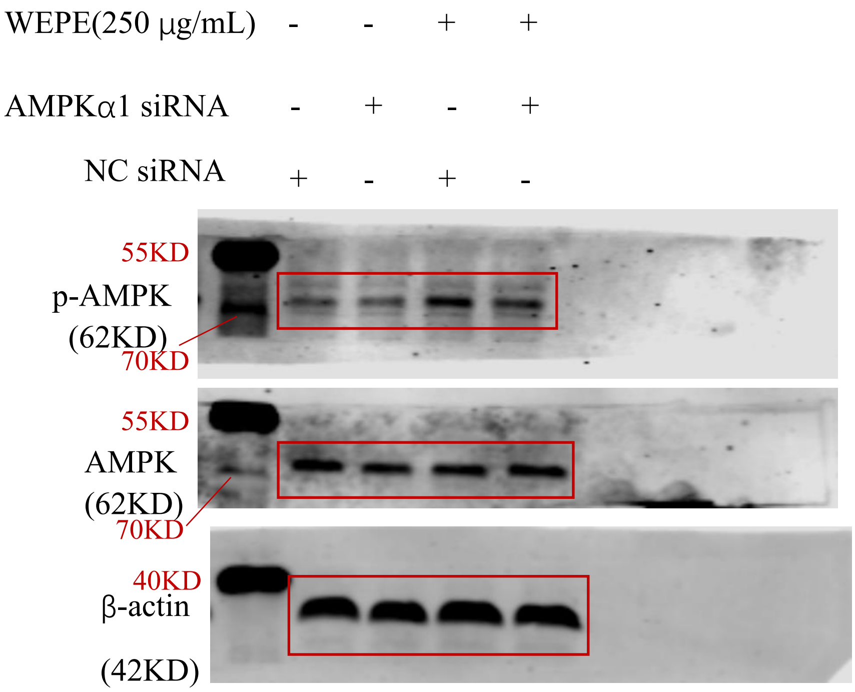
WEPE(250 μg/mL) - - + +
AMPKα1 siRNA - + - +
NC siRNA + - + -
55KD
p-AMPK
(62KD)
70KD
55KD
AMPK
(62KD)
70KD
40KD
β-actin
(42KD)

Figure 4A p-ACC/β-actin

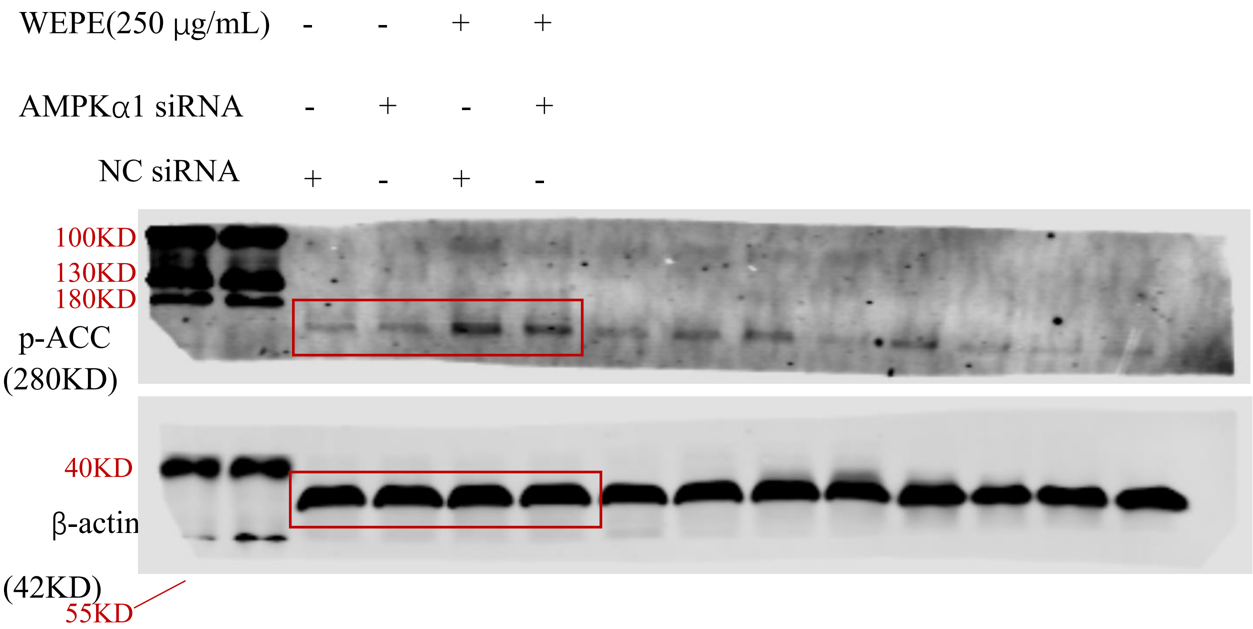

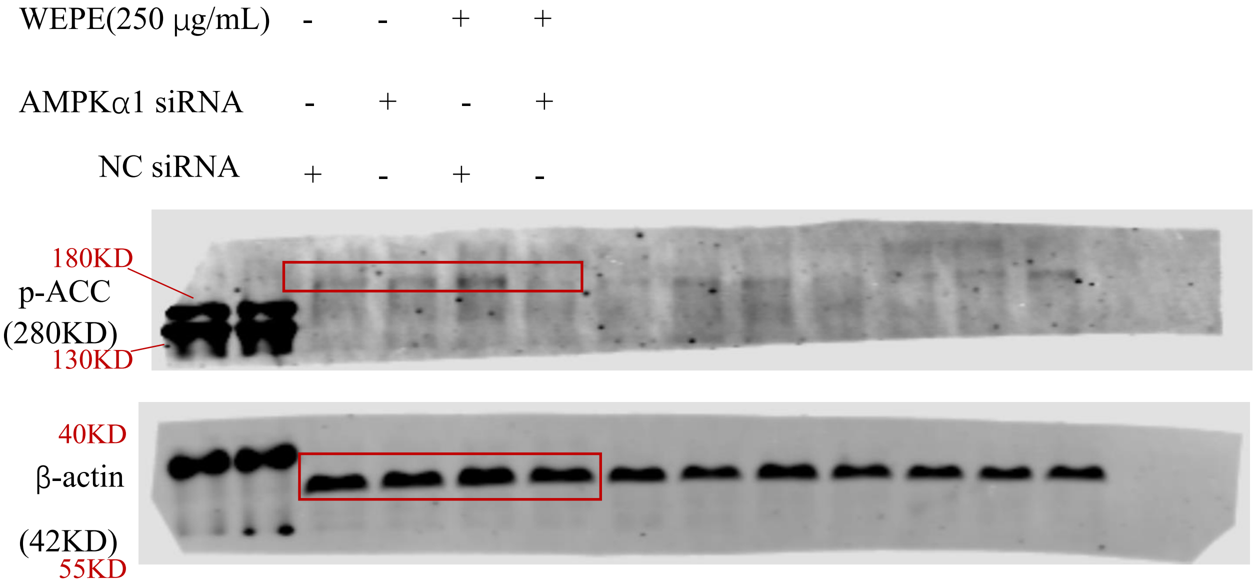

Figure 4A GLUT4/Na^+-K^+ATPase

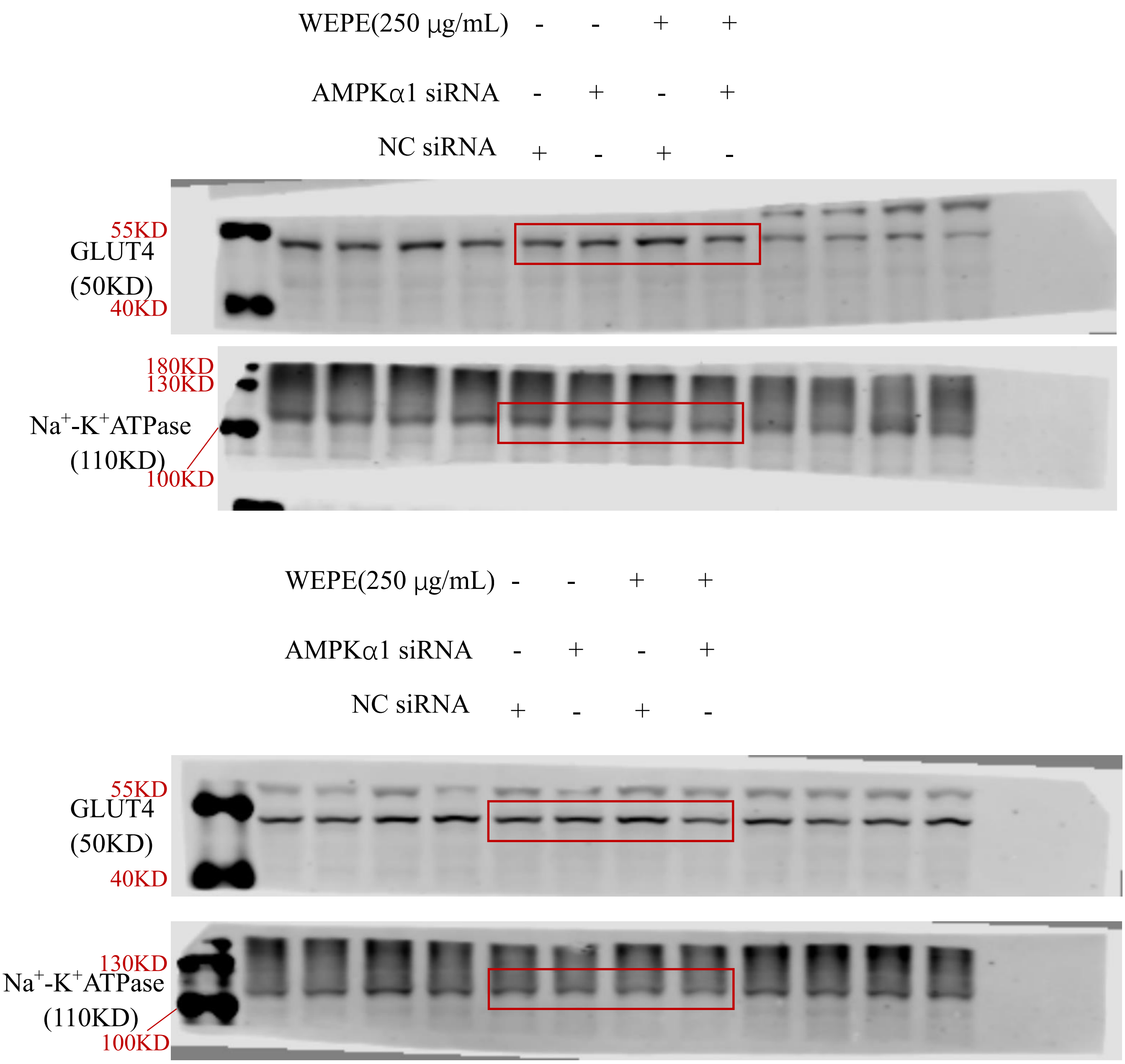

Figure 5A p-AMPK/AMPK

WEPE(250 μg/mL) - - + +

STO-609(15 μM) - + - +

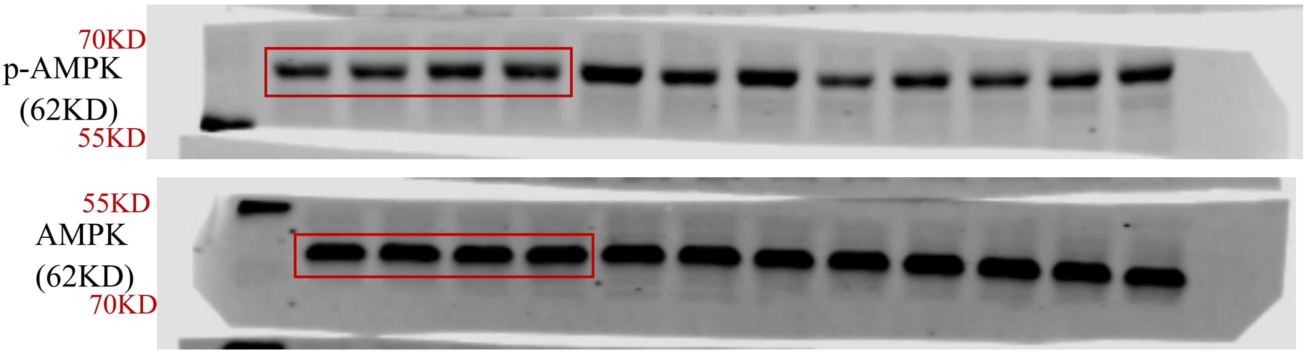

WEPE(250 μg/mL) - - + +

STO-609(15 μM) - + - +

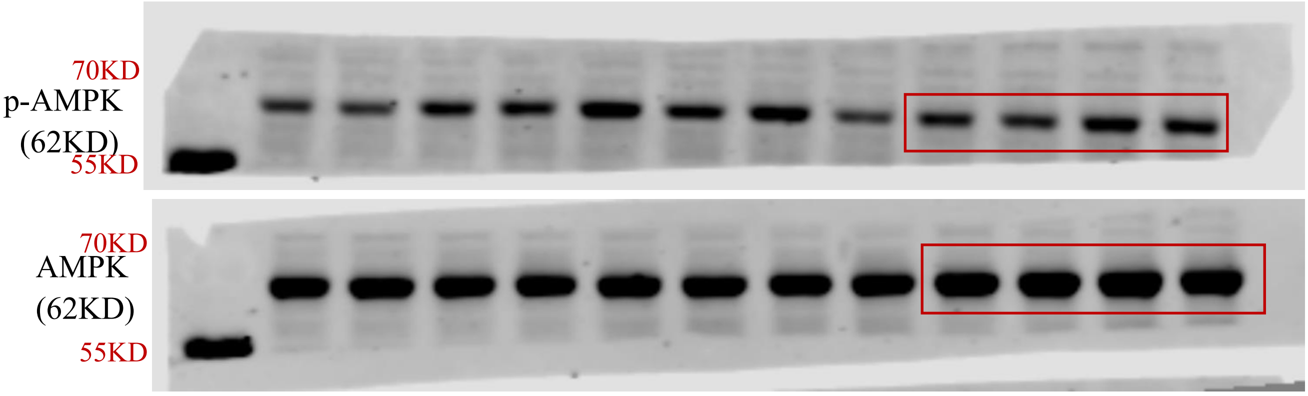

WEPE(250 μg/mL) - - + +

STO-609(15 μM) - + - +

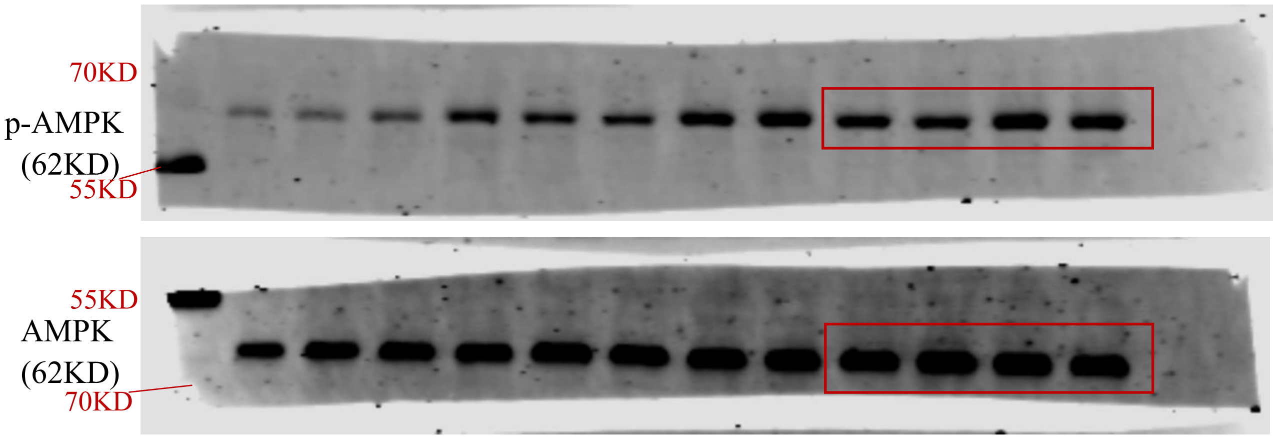

Figure 5A p-ACC/ACC

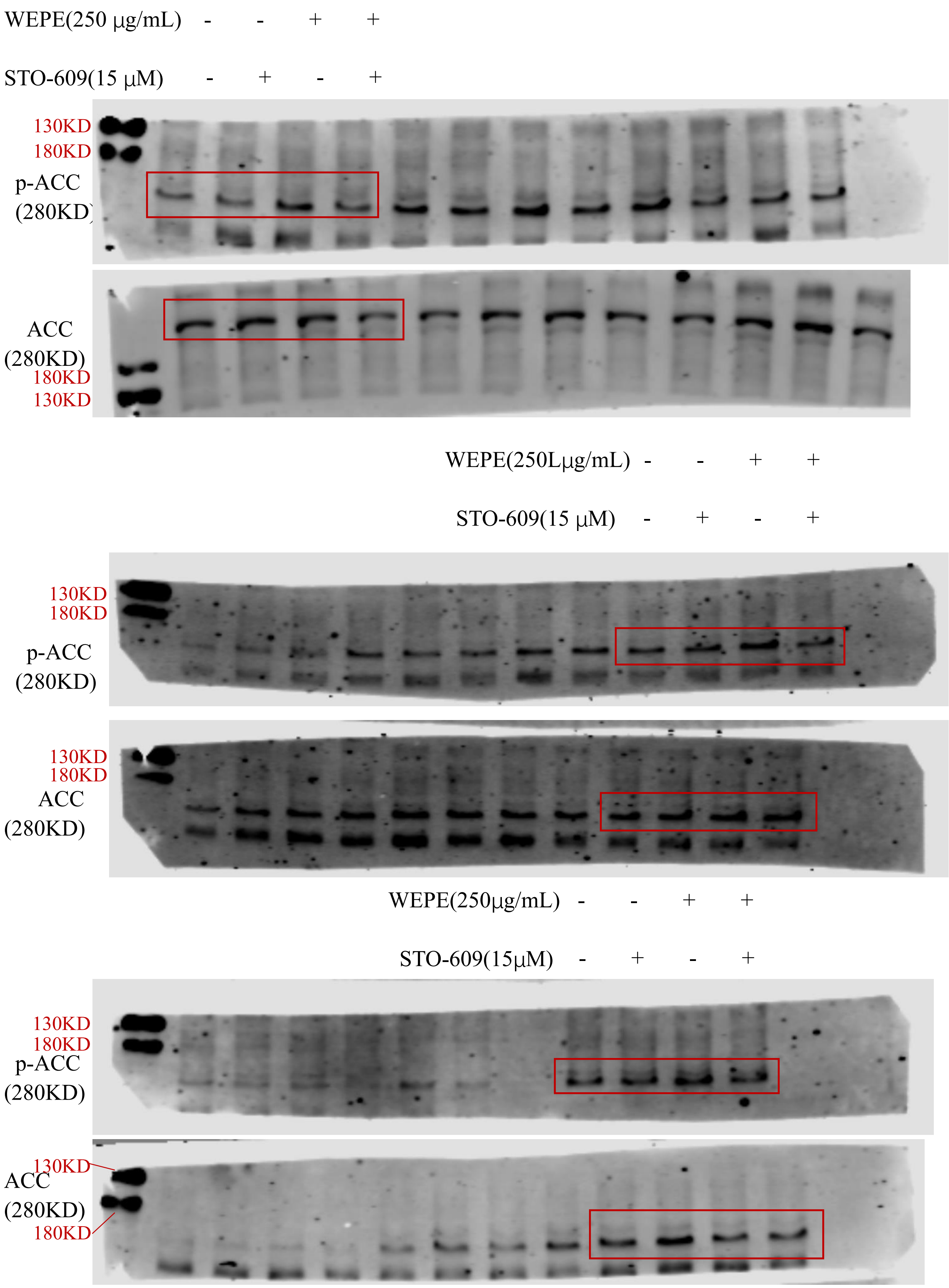

Figure 5A   p-AS160/β-actin

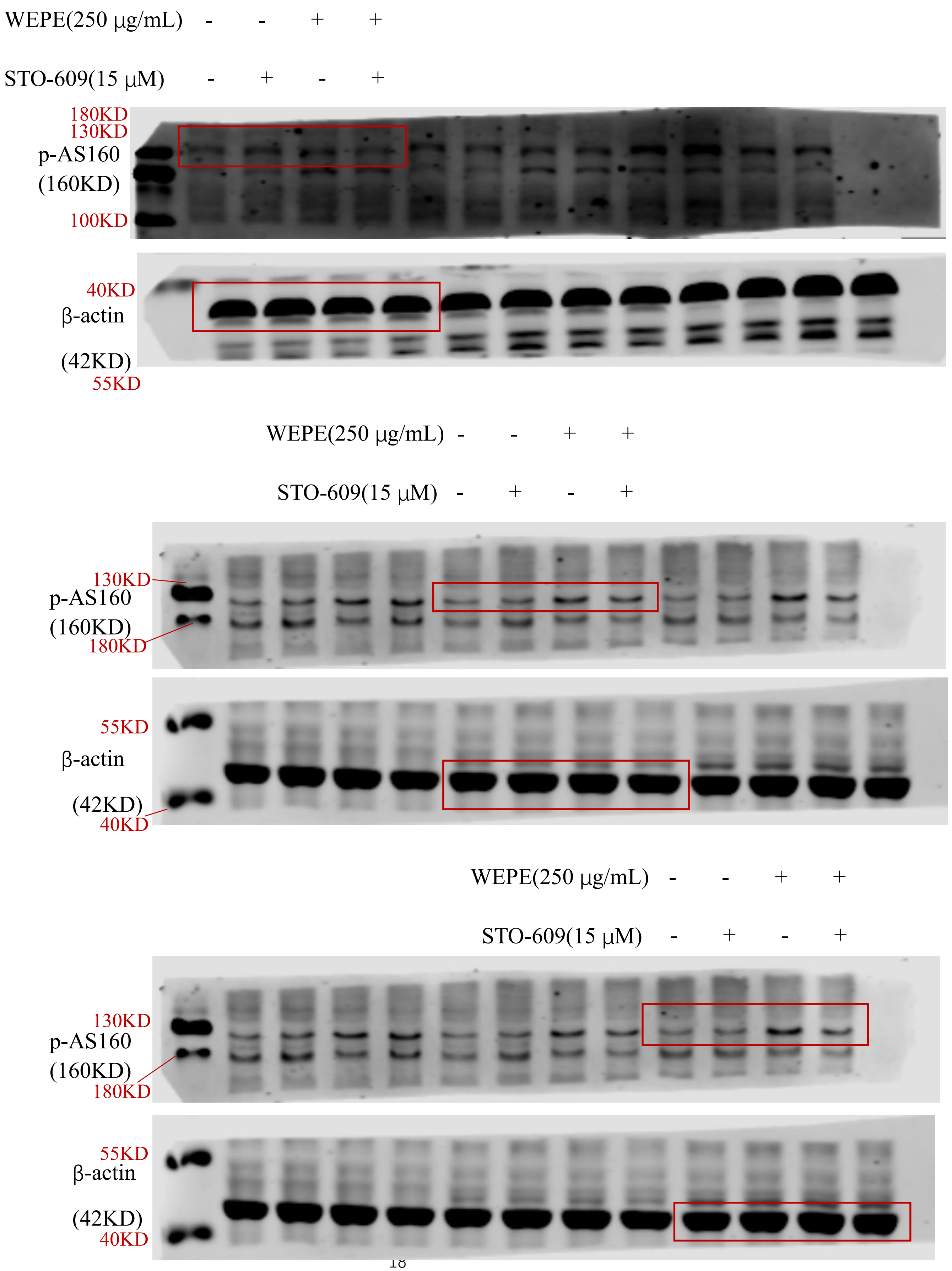

Figure 5A p-p38MAPK/p38MAPK

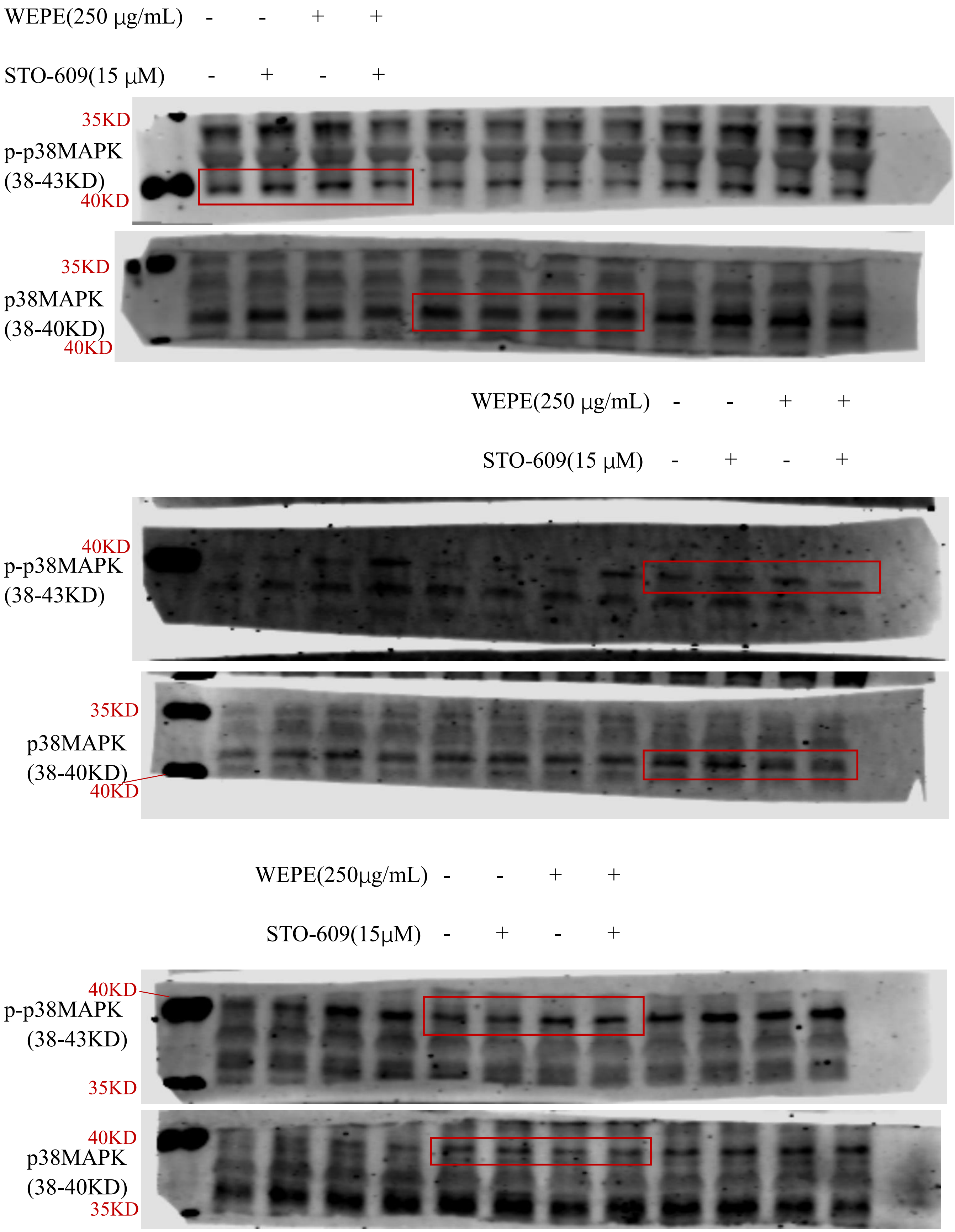

Figure 5A GLUT4/Na^+-K^+ATPase

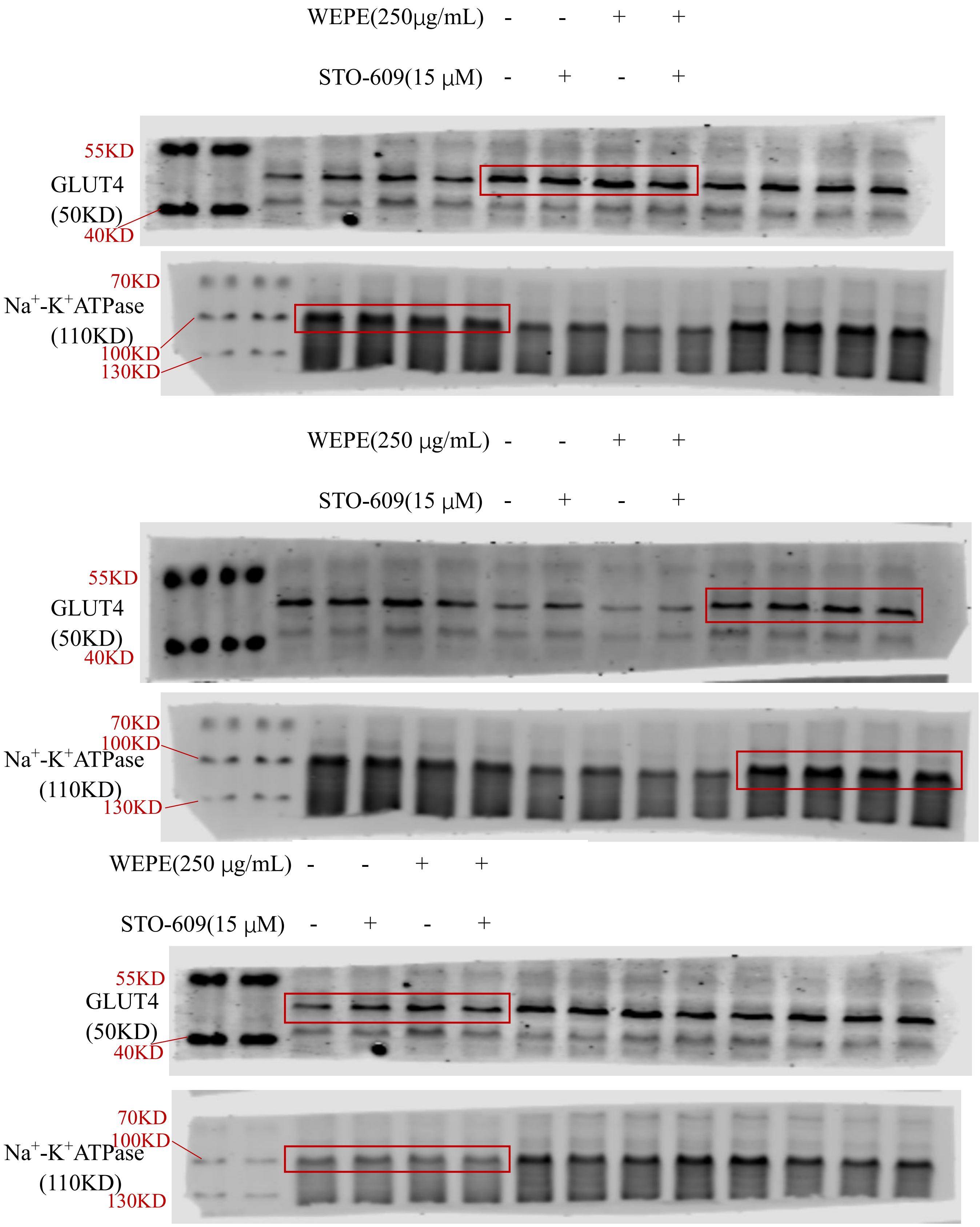

Figure 6A p-AKT (ser473)/AKT

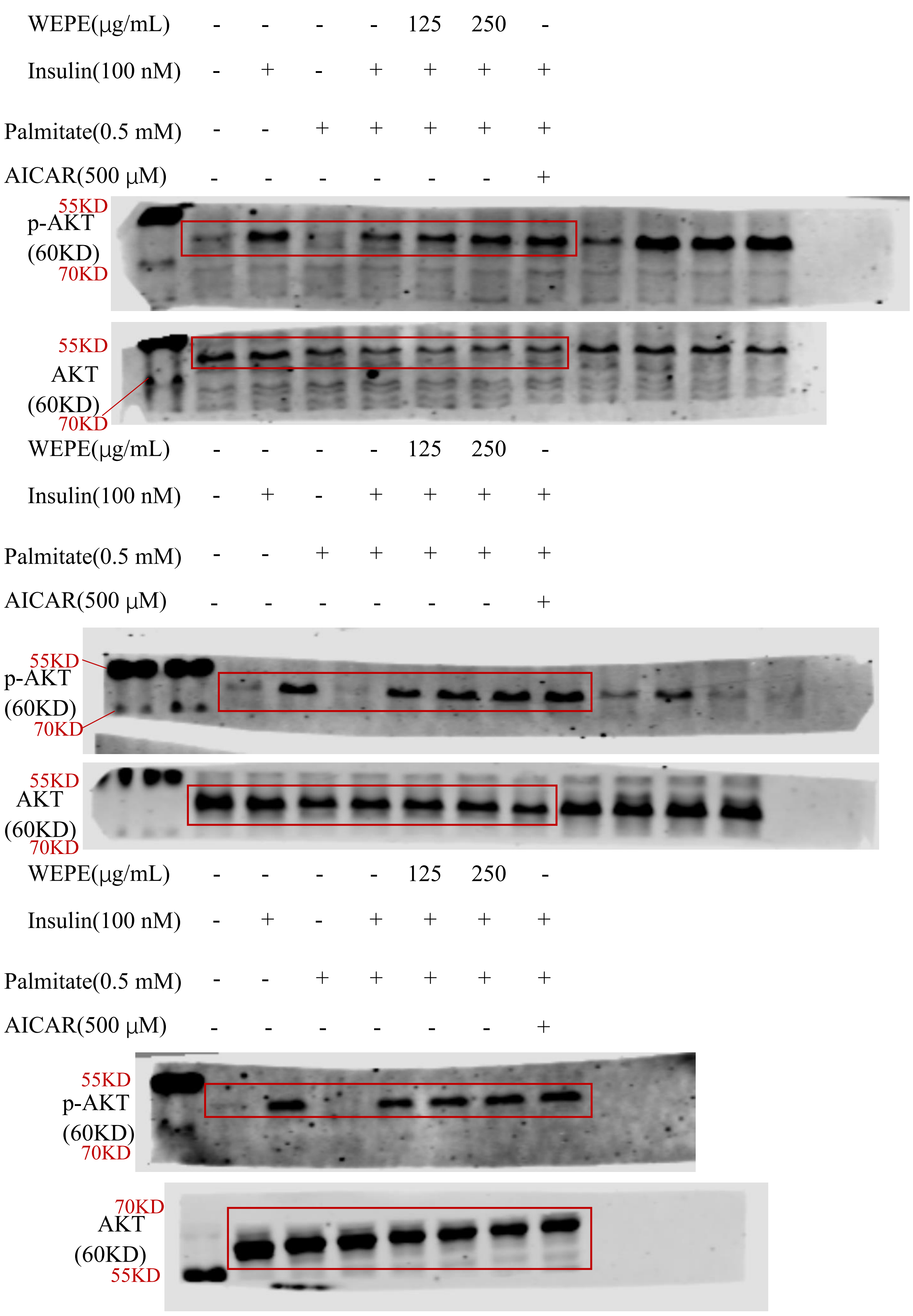

Figure 7A p-AKMPK/AMPK

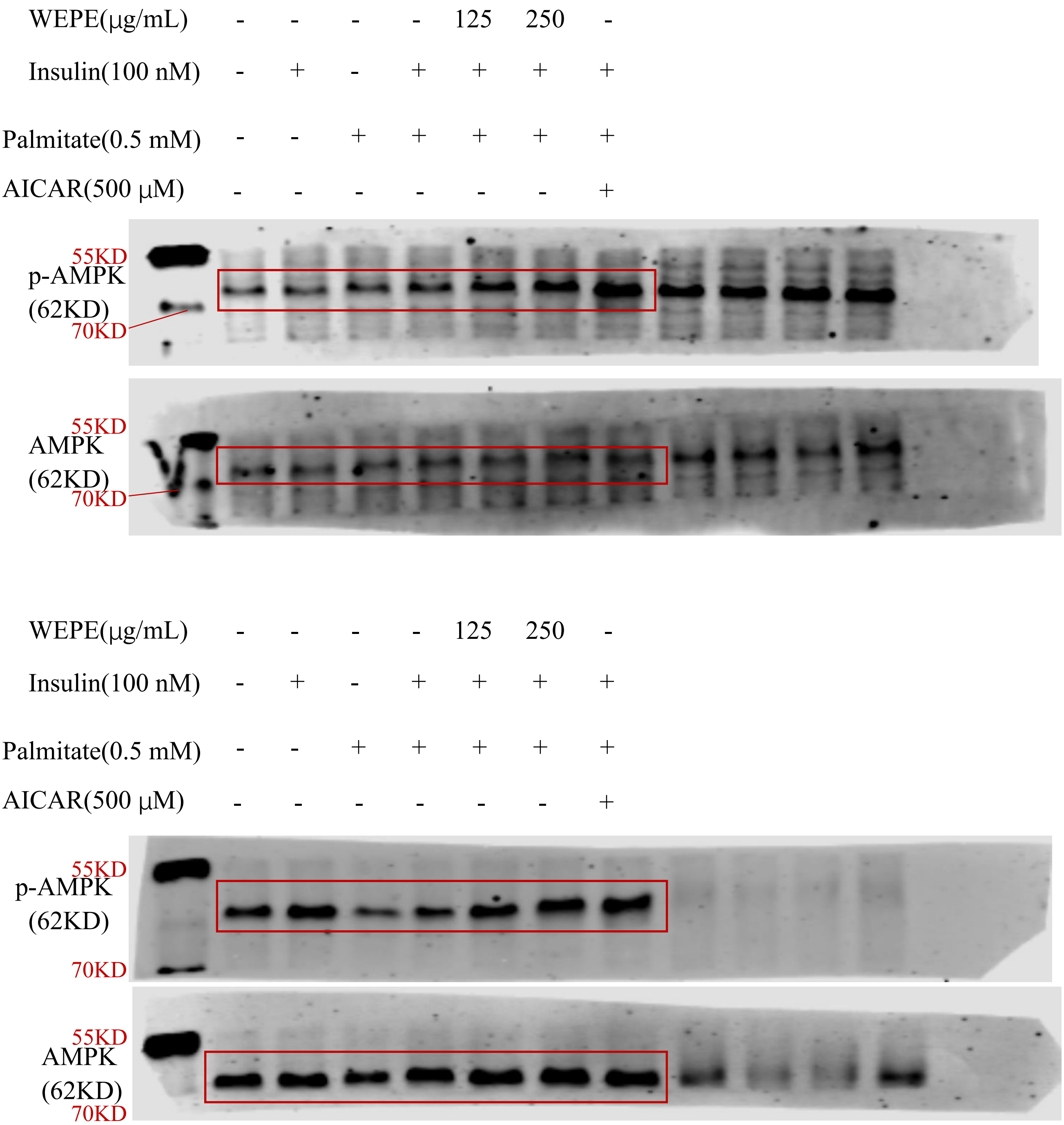

WEPE(μg/mL)	-	-	-	-	125	250	-
Insulin(100 nM)	-	+	-	+	+	+	+
Palmitate(0.5 mM)	-	-	+	+	+	+	+
AICAR(500 μM)	-	-	-	-	-	-	+

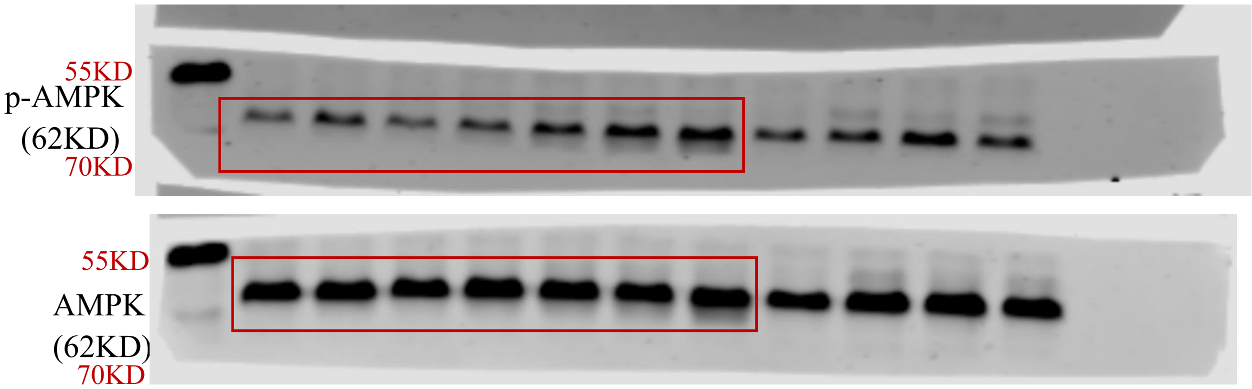

Figure 7A p-ACC/ACC

WEPE(μg/mL)	-	-	-	-	125	250	-
Insulin(100 nM)	-	+	-	+	+	+	+
Palmitate(0.5 mM)	-	-	+	+	+	+	+
AICAR(500 μM)	-	-	-	-	-	-	+

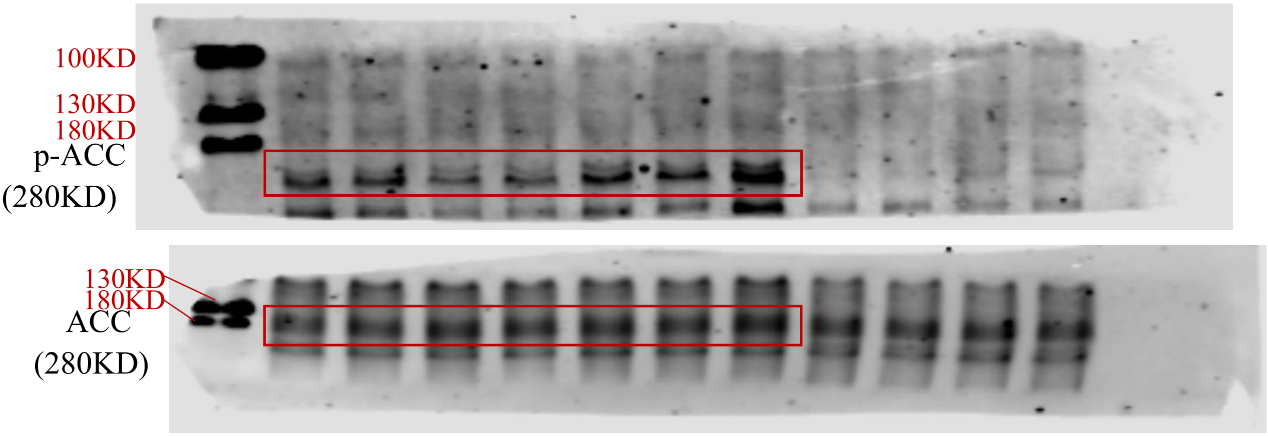

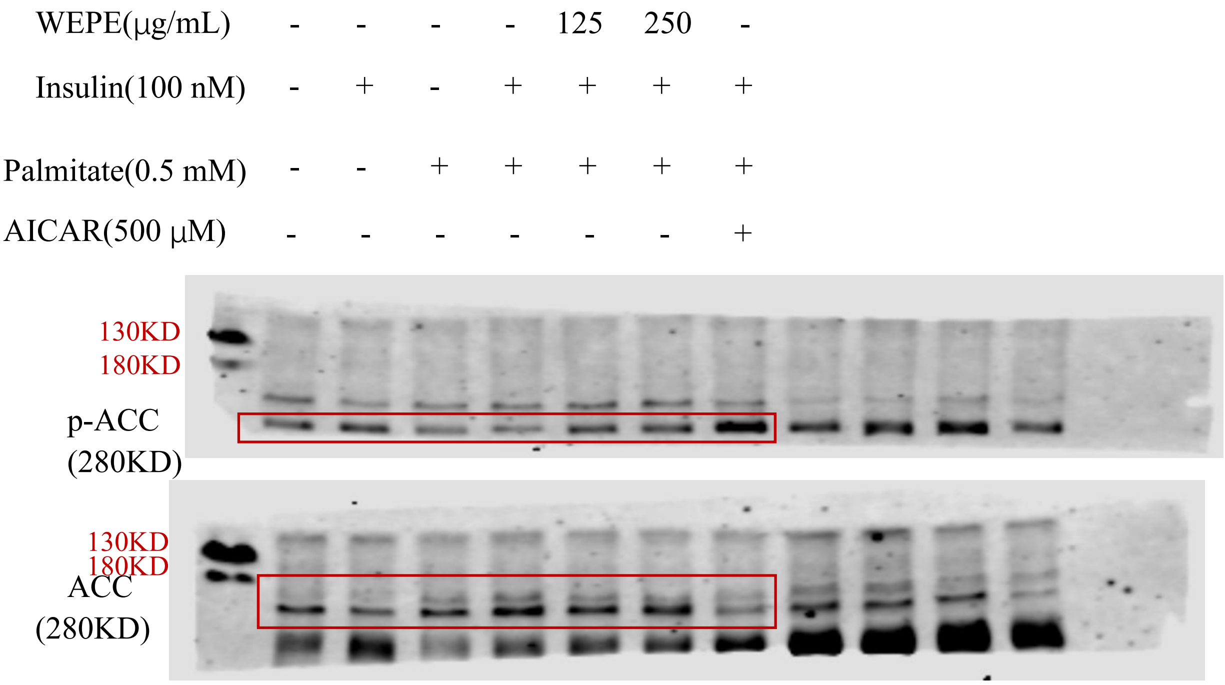
WEPE(μg/mL) - - - - 125 250 -
Insulin(100 nM) - + - + + + +
Palmitate(0.5 mM) - - + + + + +
AICAR(500 μM) - - - - - - +
130KD
180KD
p-ACC
(280KD)
130KD
180KD
ACC
(280KD)

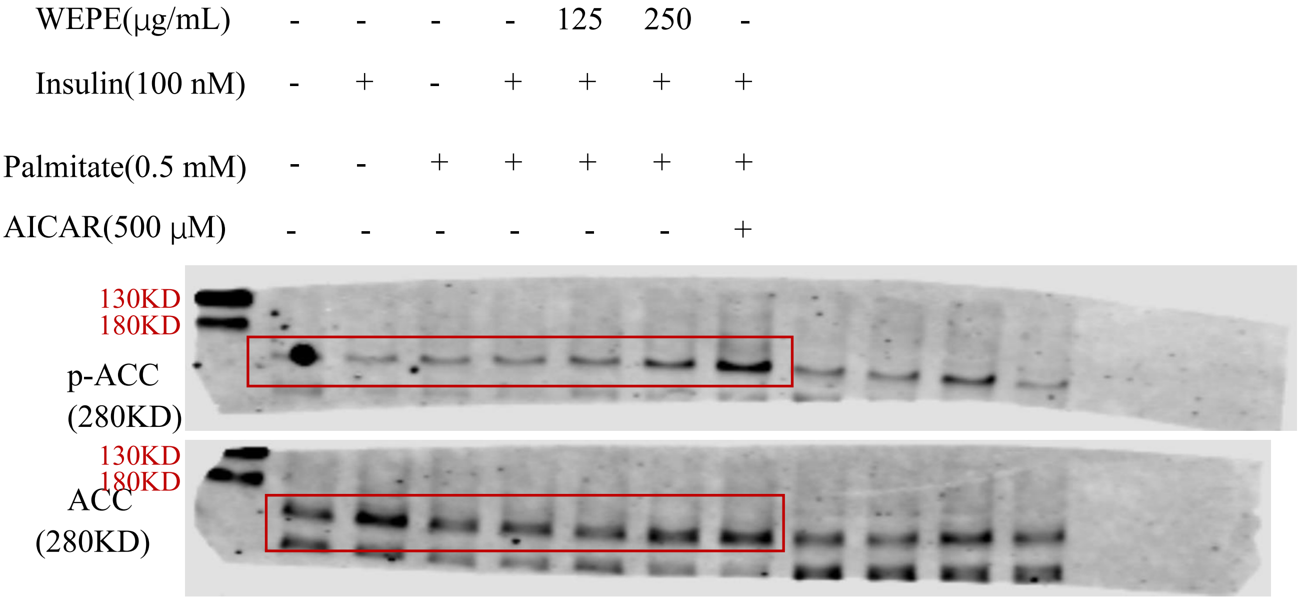
WEPE(μg/mL) - - - - 125 250 -
Insulin(100 nM) - + - + + + +
Palmitate(0.5 mM) - - + + + + +
AICAR(500 μM) - - - - - - +
130KD
180KD
p-ACC
(280KD)
130KD
180KD
ACC
(280KD)

Figure 7A p-AS160/β-actin

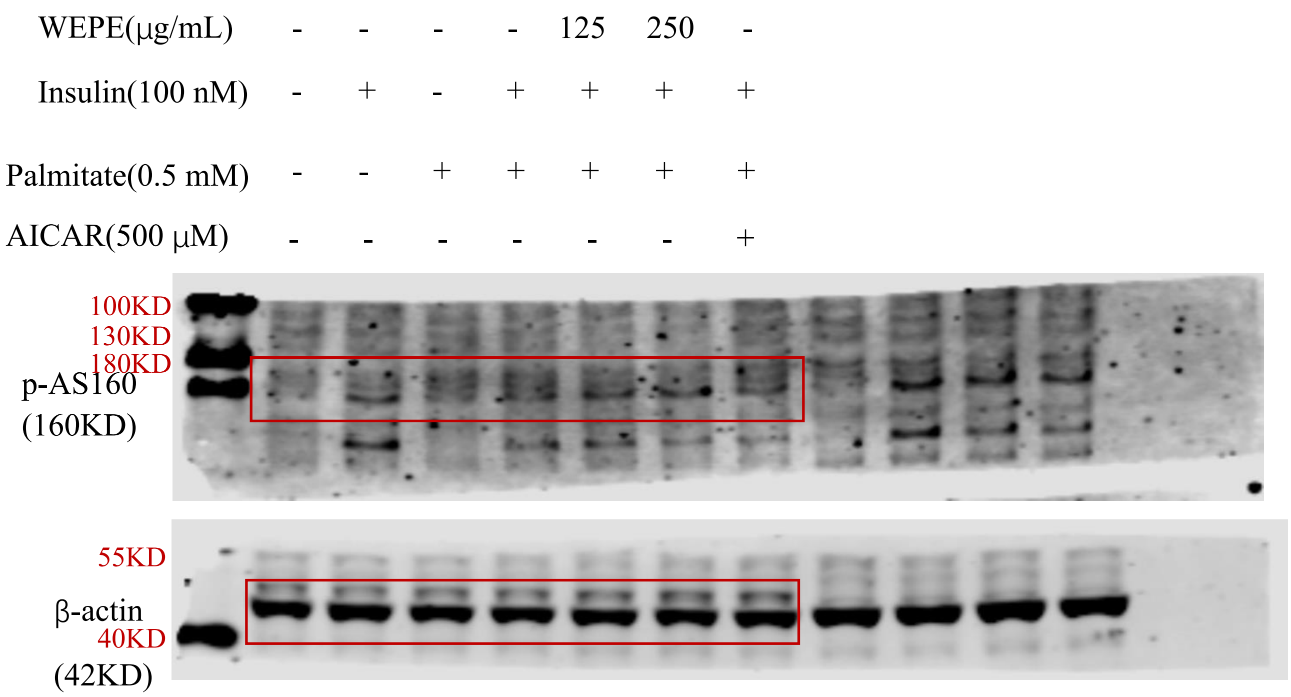

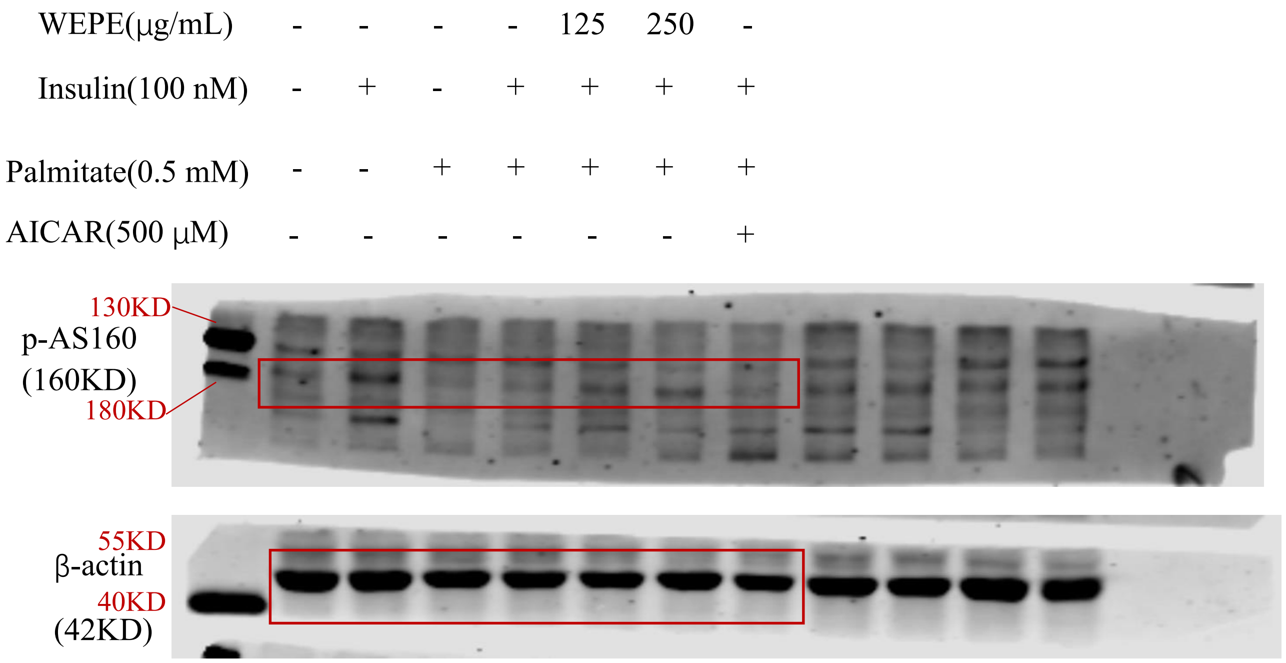

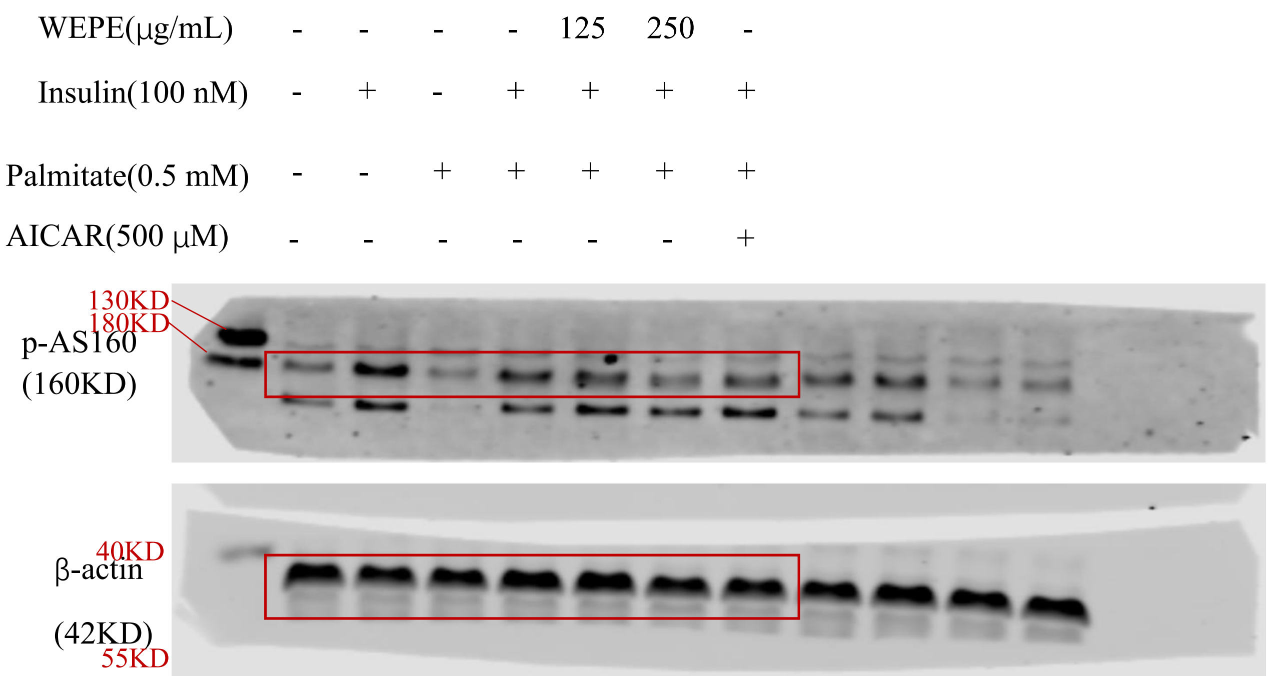
WEPE(μg/mL) - - - - 125 250 -
Insulin(100 nM) - + - + + + +
Palmitate(0.5 mM) - - + + + + +
AICAR(500 μM) - - - - - - +
130KD
180KD
p-AS160
(160KD)
40KD
β-actin
(42KD)
55KD

Figure 7C p-AMPK/AMPK

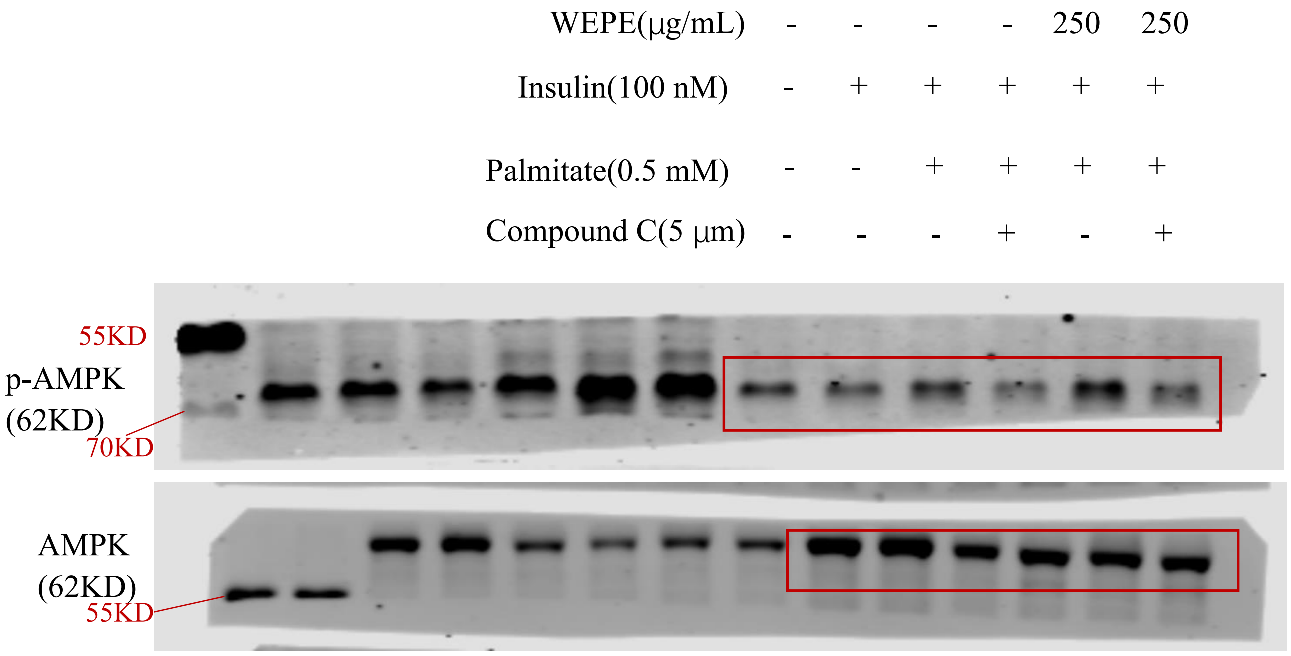

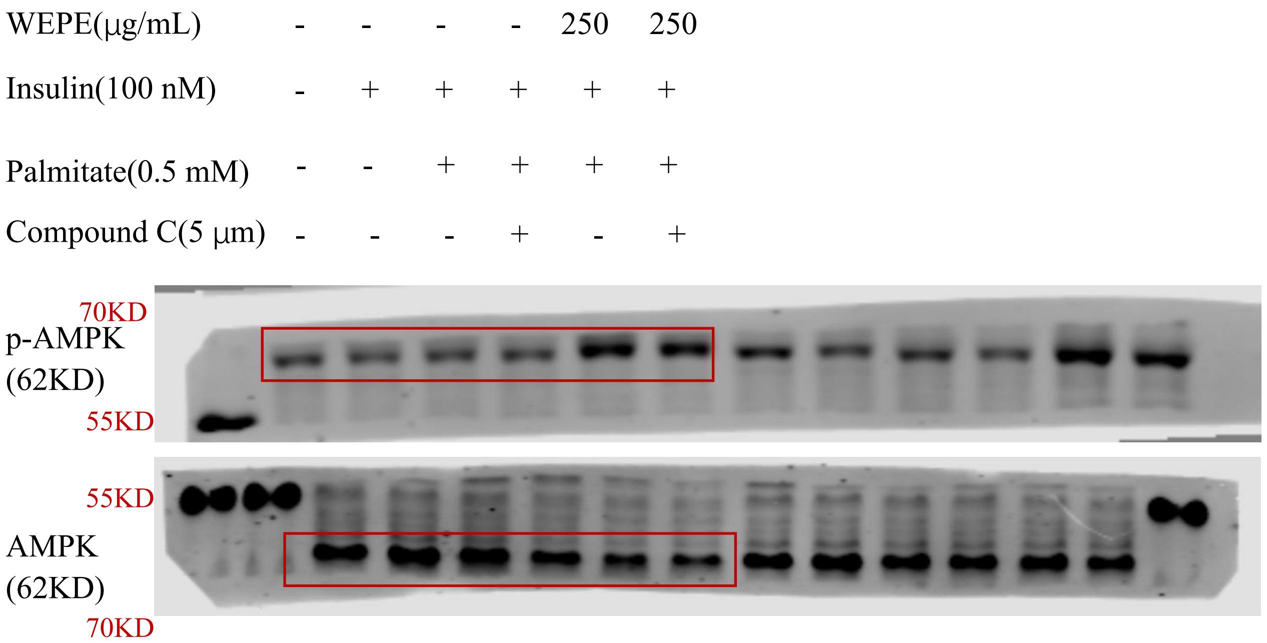

WEPE(μg/ml)	-	-	-	-	250	250
Insulin(100nM)	-	+	+	+	+	+
Palmitate(0.5mM)	-	-	+	+	+	+
Compound C(5μm)	-	-	-	+	-	+

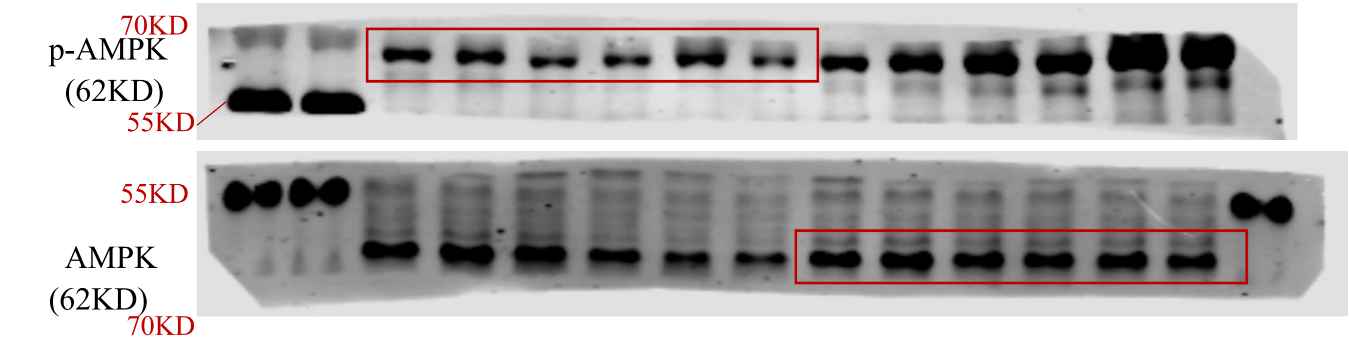

Figure 7C p-ACC/ACC

WEPE(μg/mL)	-	-	-	-	250	250
Insulin(100 nM)	-	+	+	+	+	+
Palmitate(0.5 mM)	-	-	+	+	+	+
Compound C(5 μm)	-	-	-	+	-	+

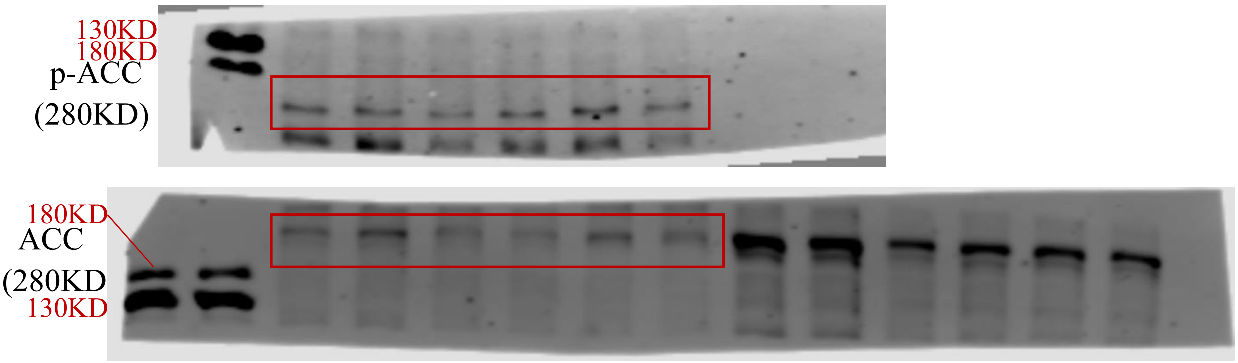

WEPE(μg/mL)	-	-	-	-	250	250
Insulin(100 nM)	-	+	+	+	+	+
Palmitate(0.5 mM)	-	-	+	+	+	+
Compound C(5 μm)	-	-	-	+	-	+

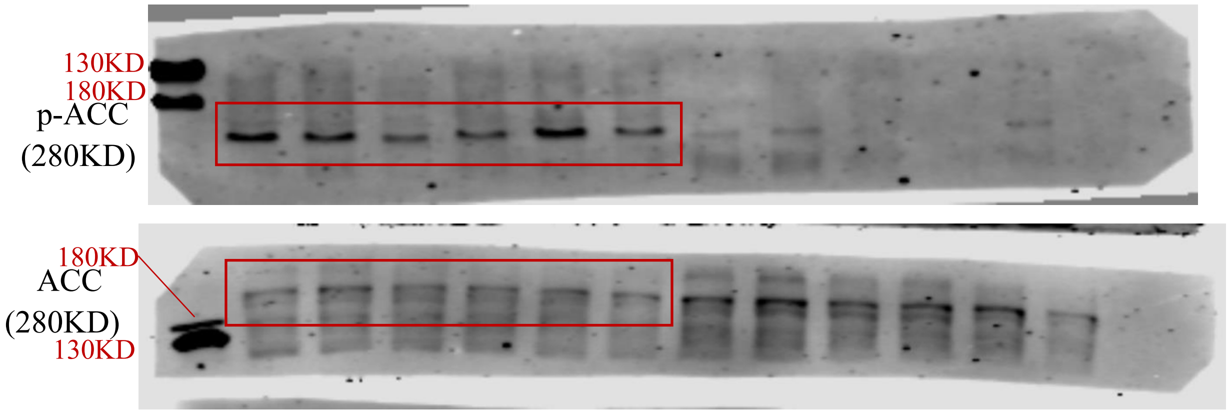

WEPE(μg/mL)	-	-	-	-	250	250
Insulin(100 nM)	-	+	+	+	+	+
Palmitate(0.5 mM)	-	-	+	+	+	+
Compound C(5 μm)	-	-	-	+	-	+

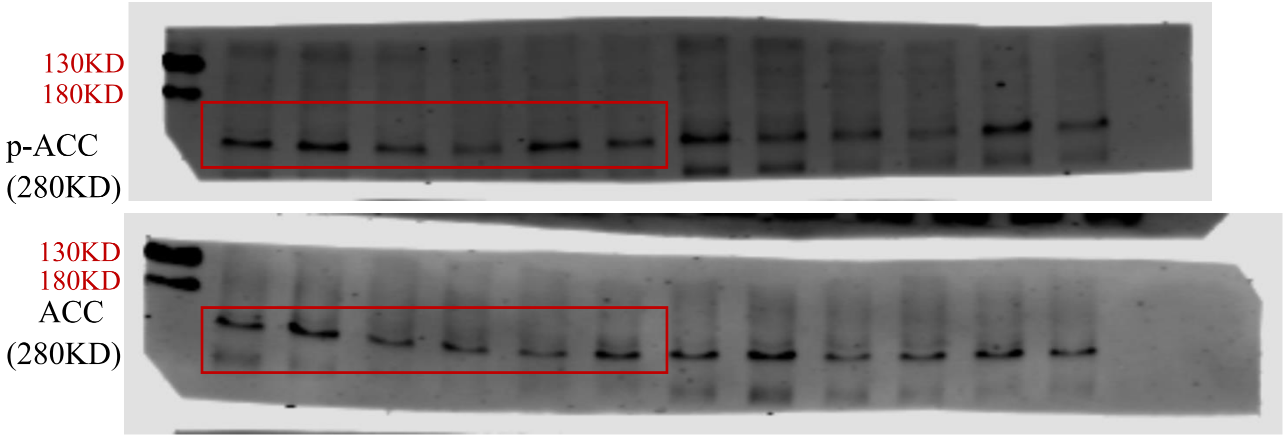

Figure 7C p-AS160/β-actin

WEPE(μg/mL)	-	-	-	-	250	250
Insulin(100 nM)	-	+	+	+	+	+
Palmitate(0.5 mM)	-	-	+	+	+	+
Compound C(5 μm)	-	-	-	+	-	+

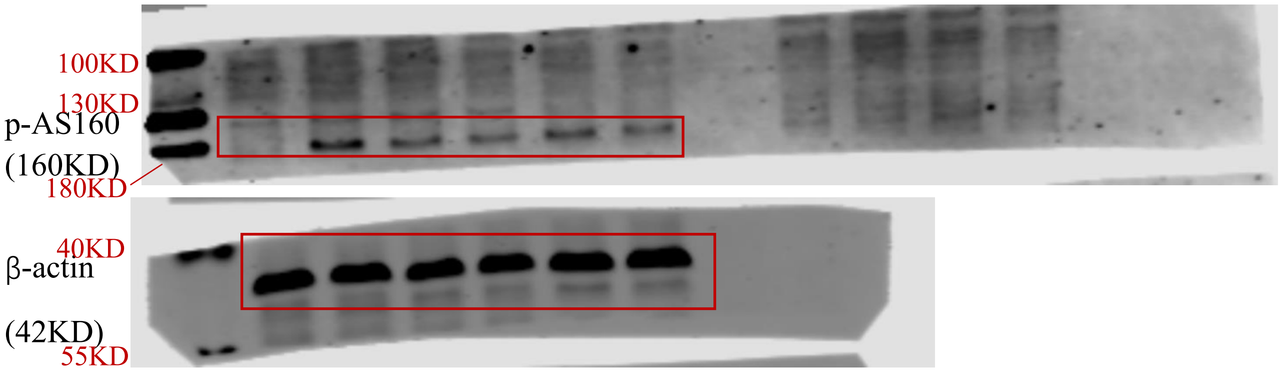

WEPE(μg/mL)	-	-	-	-	250	250
Insulin(100 nM)	-	+	+	+	+	+
Palmitate(0.5 mM)	-	-	+	+	+	+
Compound C(5 μm)	-	-	-	+	-	+

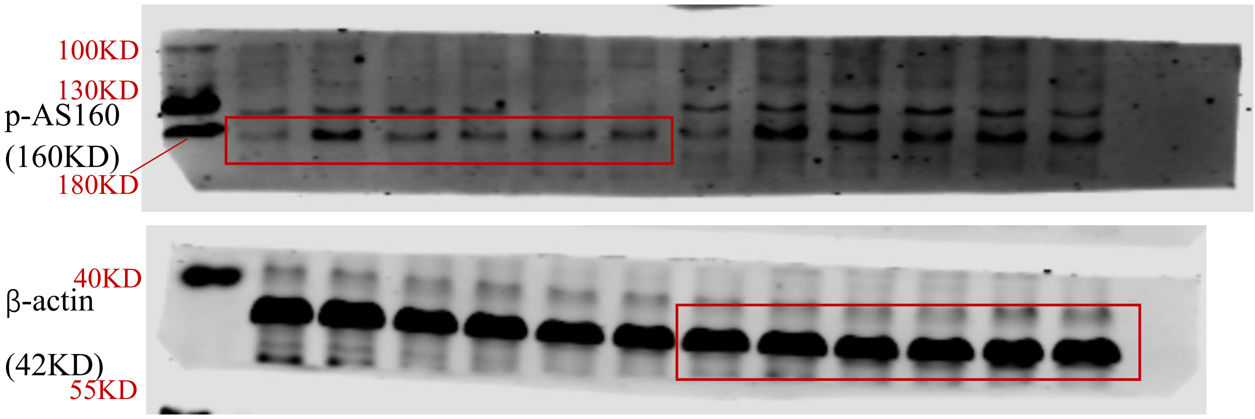

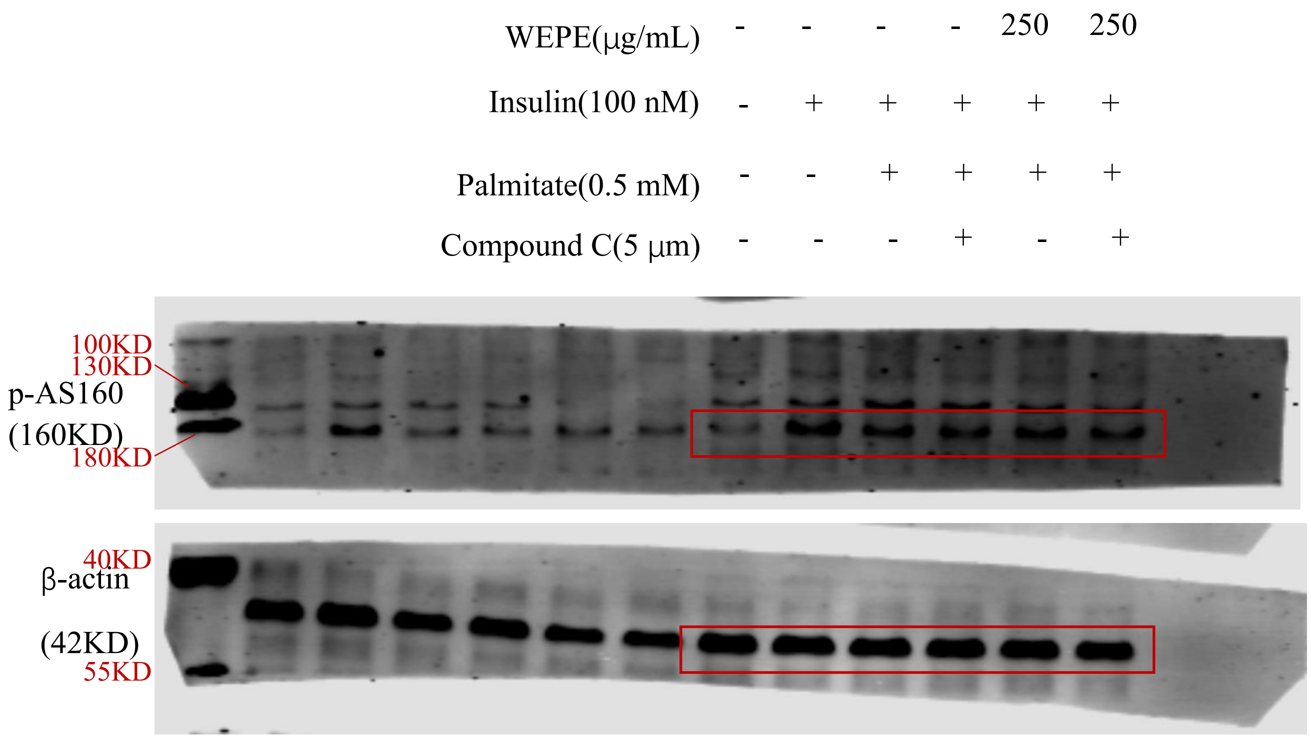

Figure 7C p-AKT/AKT

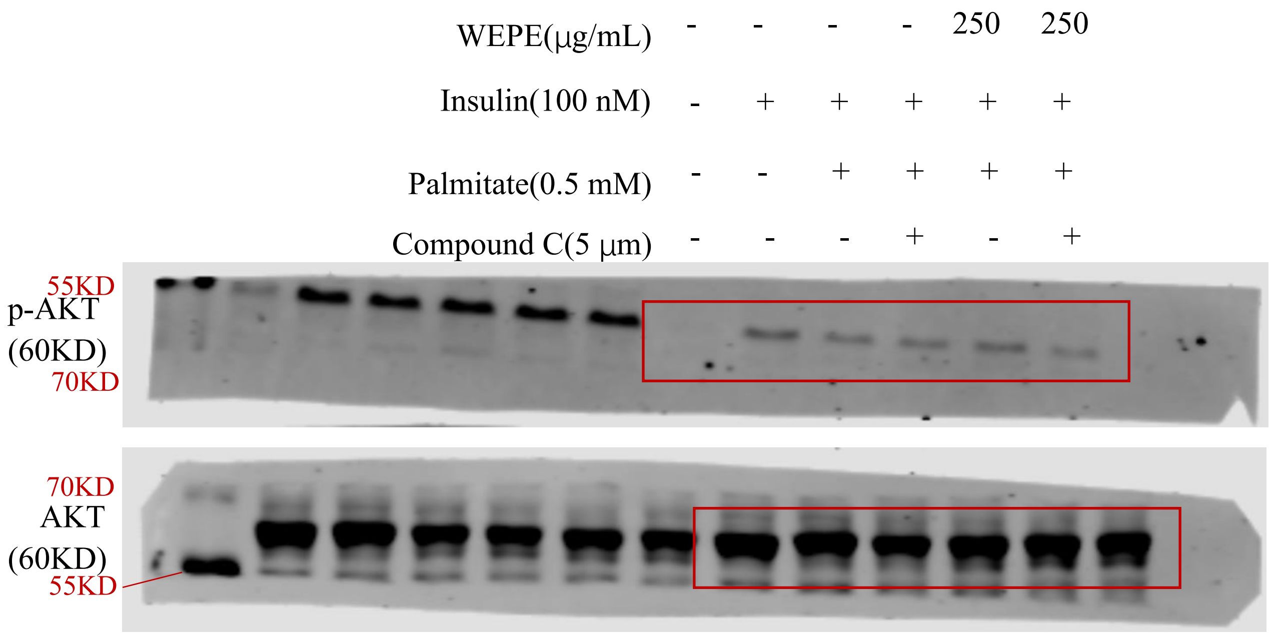

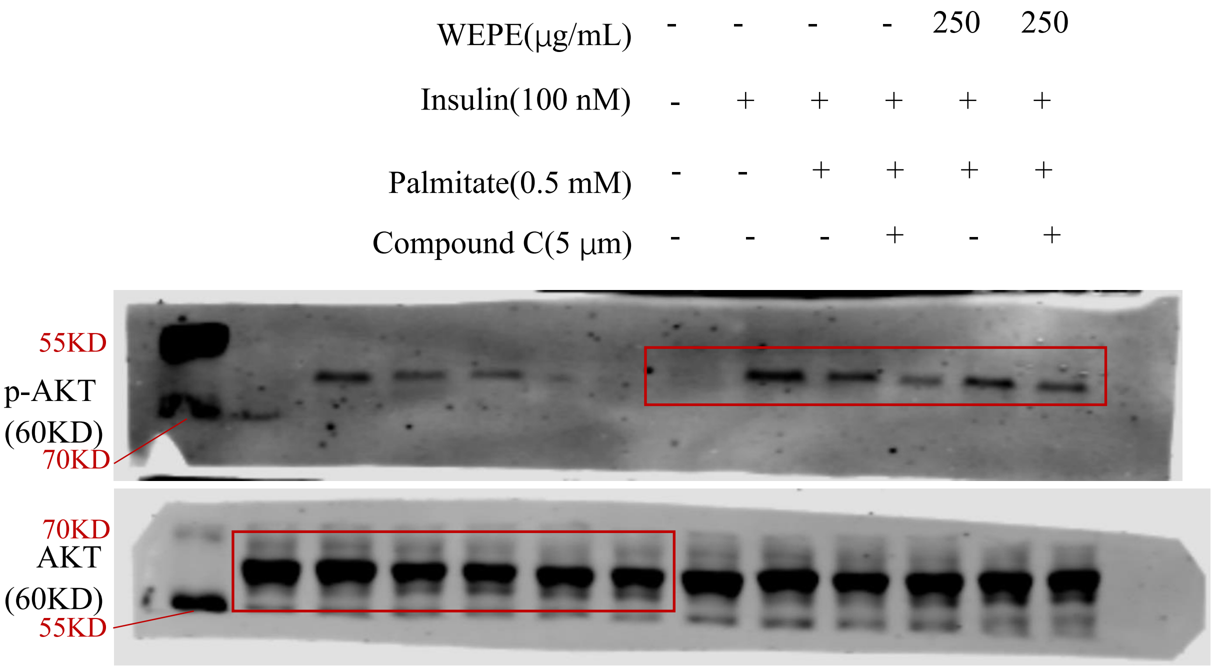
WEPE(μg/mL) - - - - 250 250
Insulin(100 nM) - + + + + +
Palmitate(0.5 mM) - - + + + +
Compound C(5 μm) - - - + - +
55KD
p-AKT
(60KD)
70KD
70KD
AKT
(60KD)
55KD

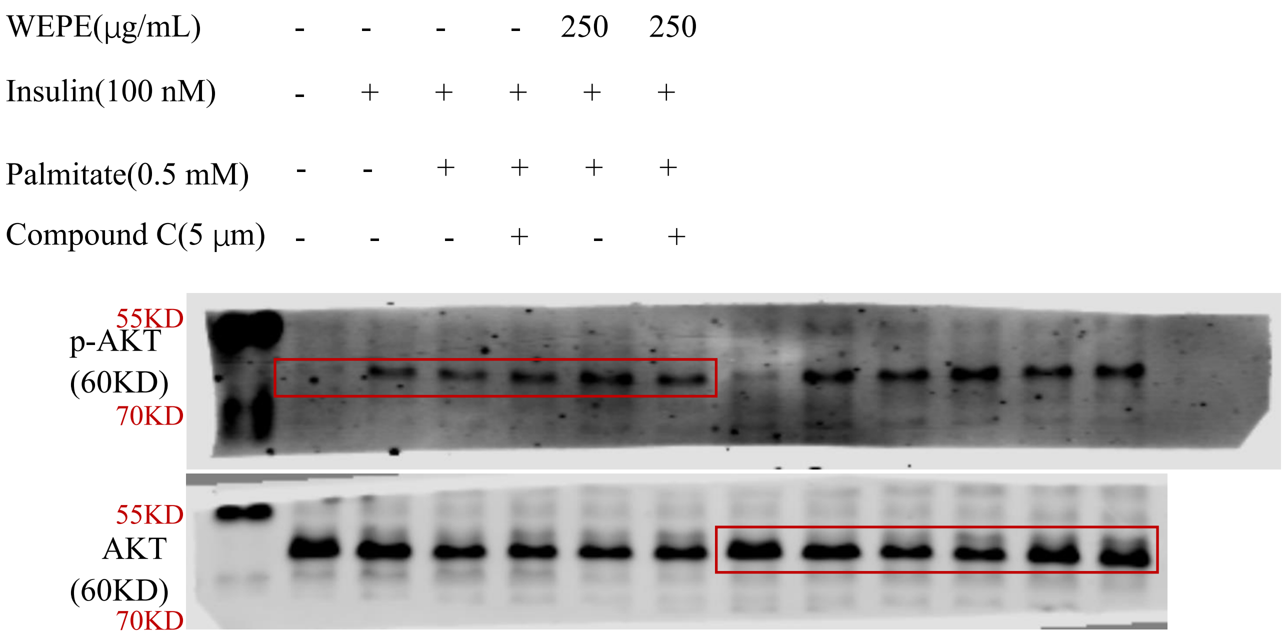
WEPE(μg/mL) - - - - 250 250
Insulin(100 nM) - + + + + +
Palmitate(0.5 mM) - - + + + +
Compound C(5 μm) - - - + - +
55KD
p-AKT
(60KD)
70KD
55KD
AKT
(60KD)
70KD

Figure 7C GLUT4/Na^{+}-K^{+}ATPase

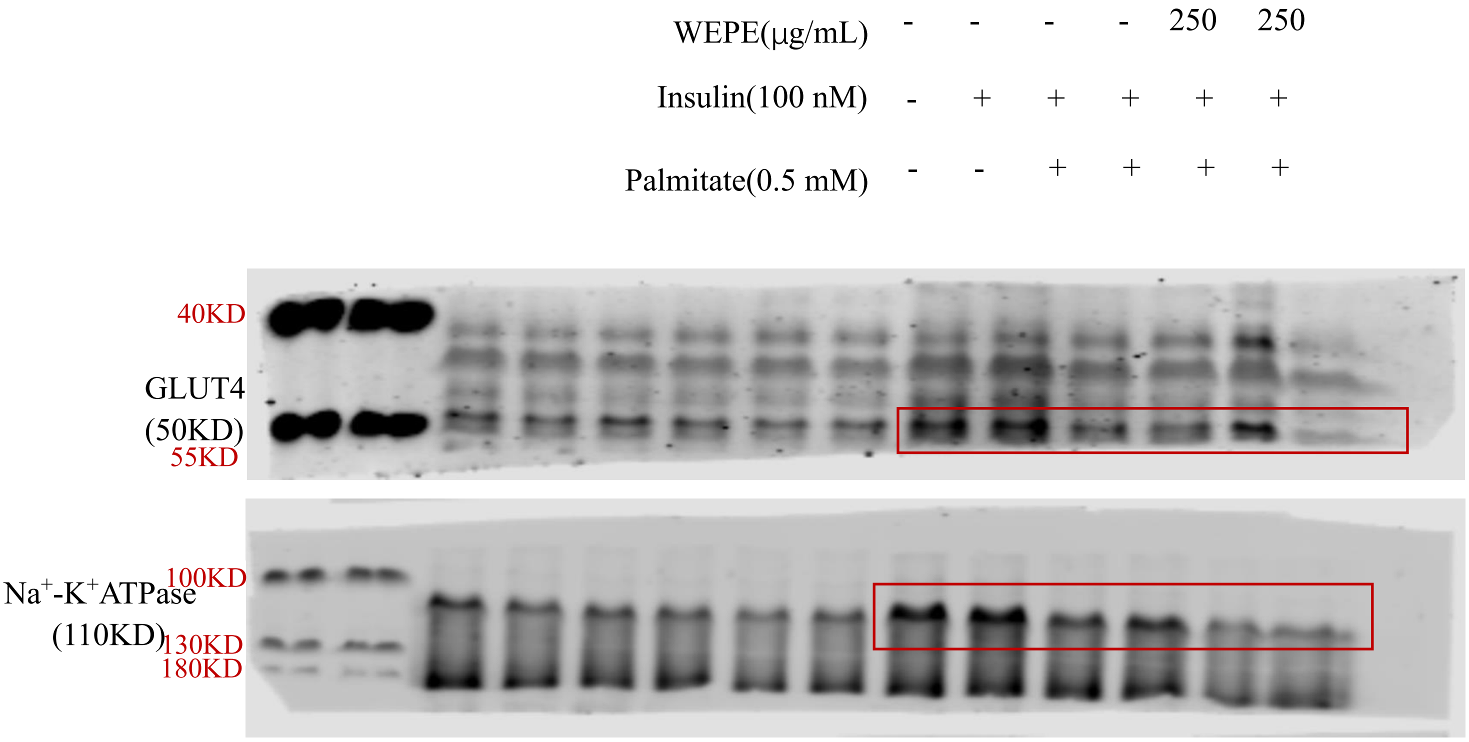

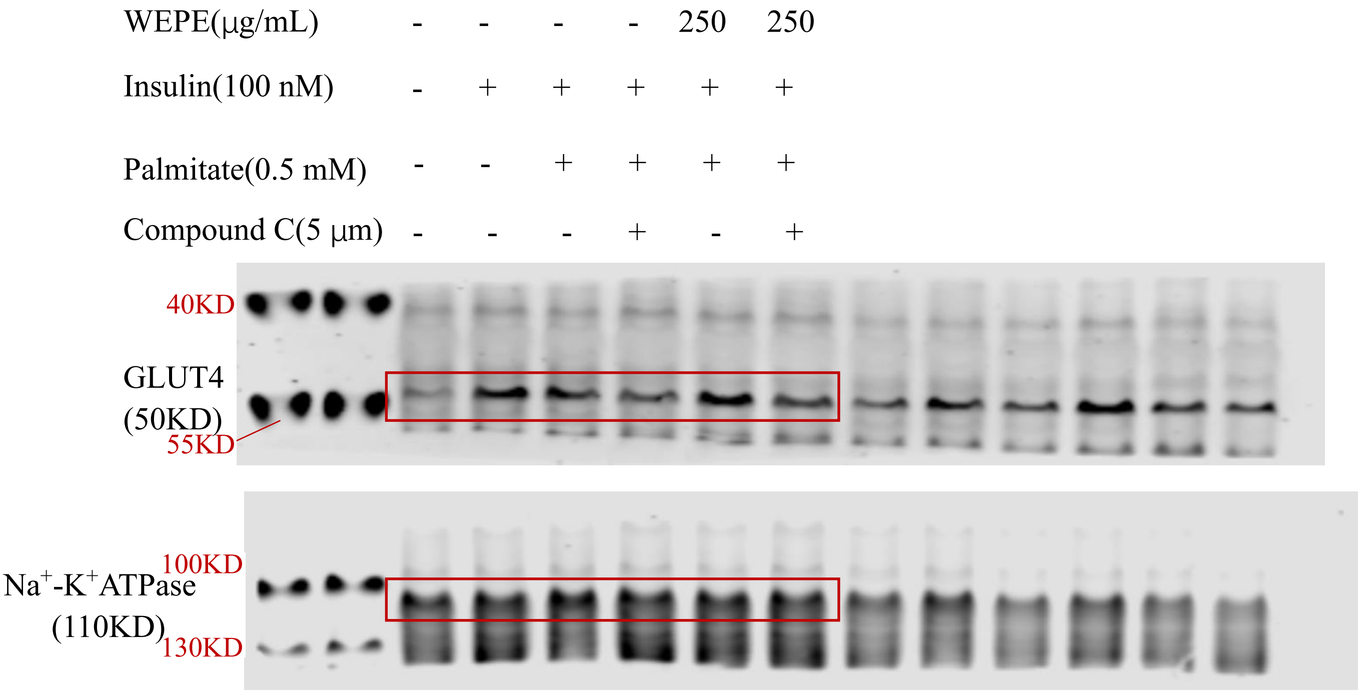

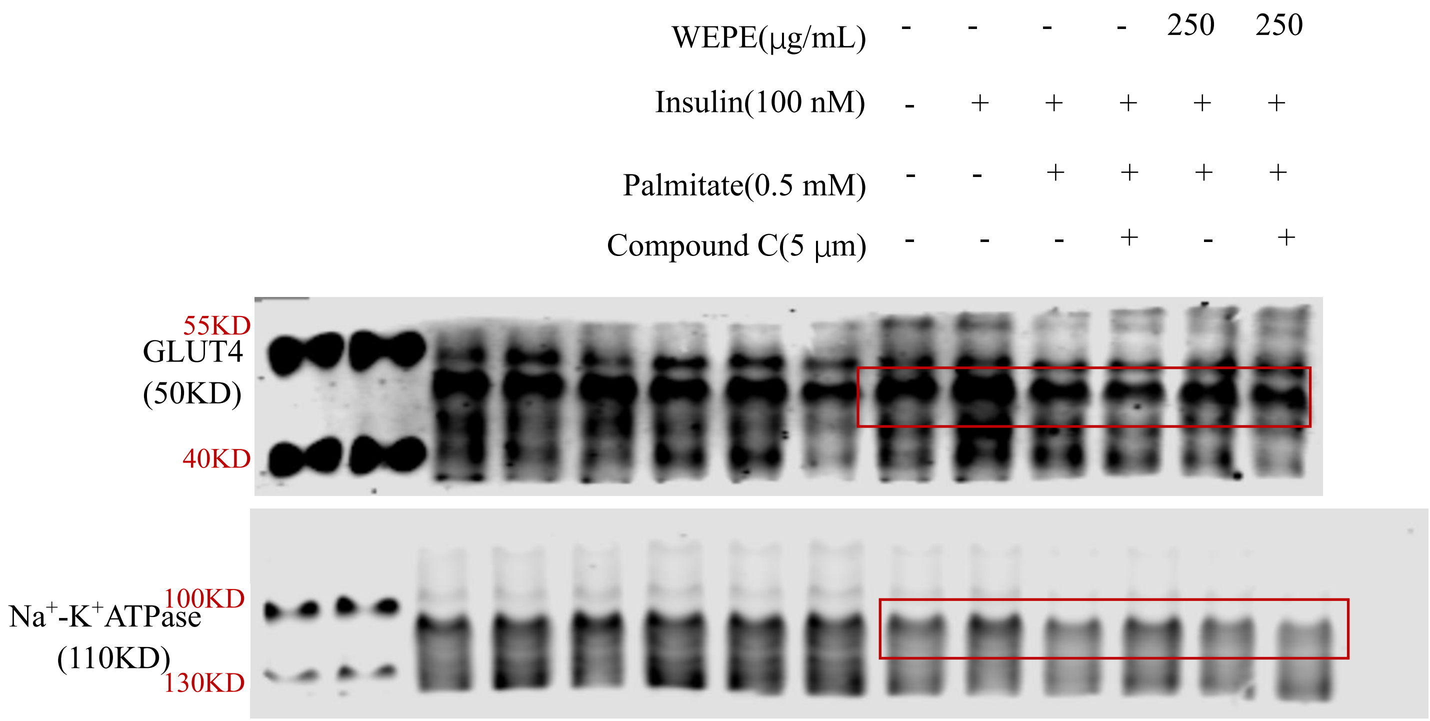

Figure 7C PKCθ/β-actin

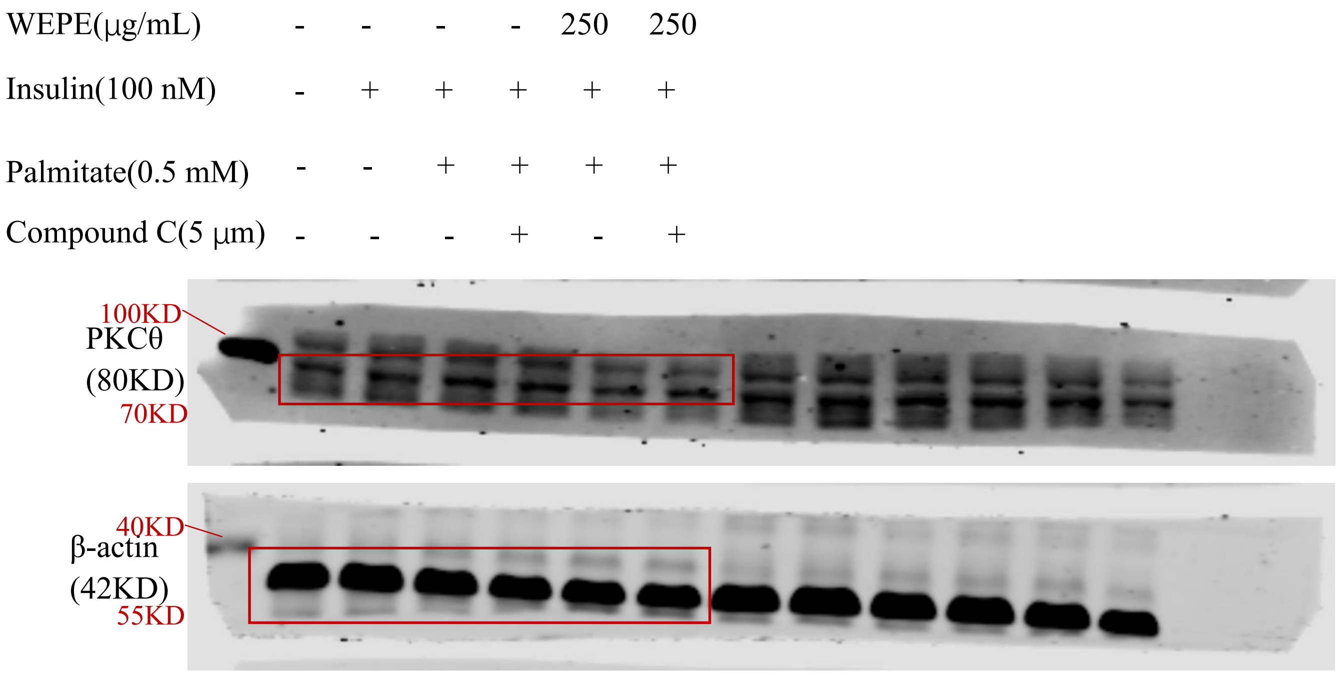

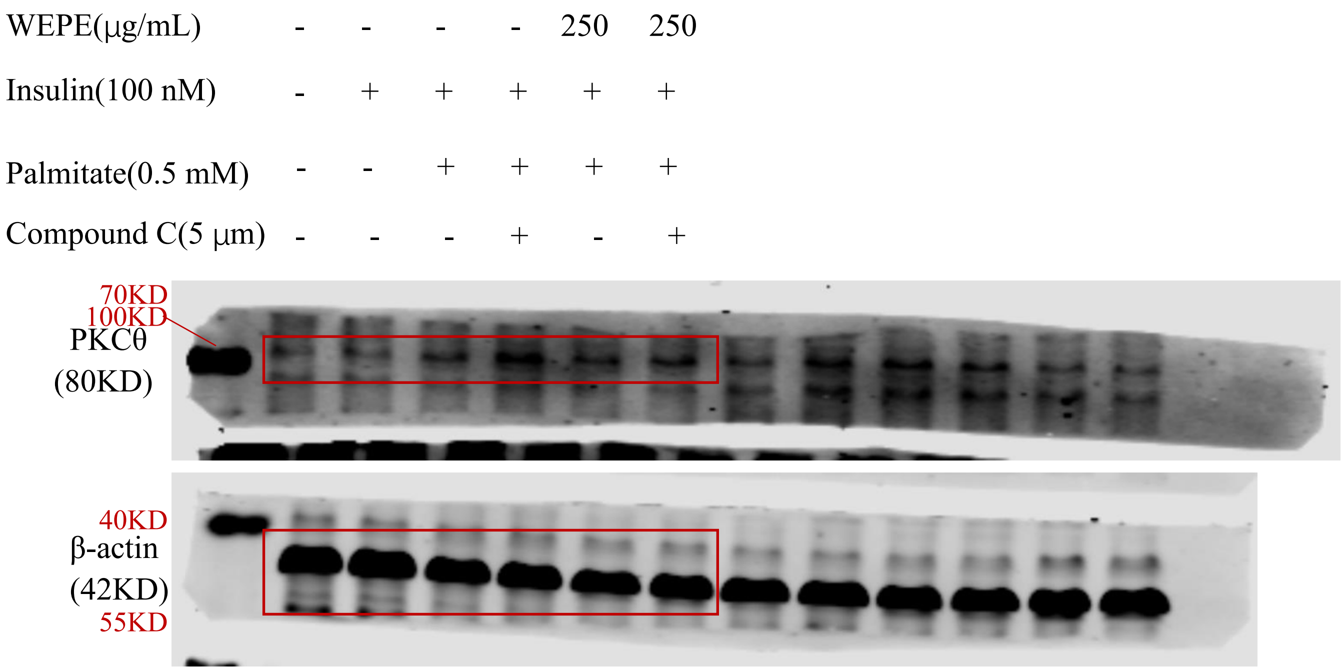
WEPE(µg/mL) - - - - 250 250
Insulin(100 nM) - + + + + +
Palmitate(0.5 mM) - - + + + +
Compound C(5 µm) - - - + - +
70KD
100KD
PKCθ
(80KD)
40KD
β-actin
(42KD)
55KD

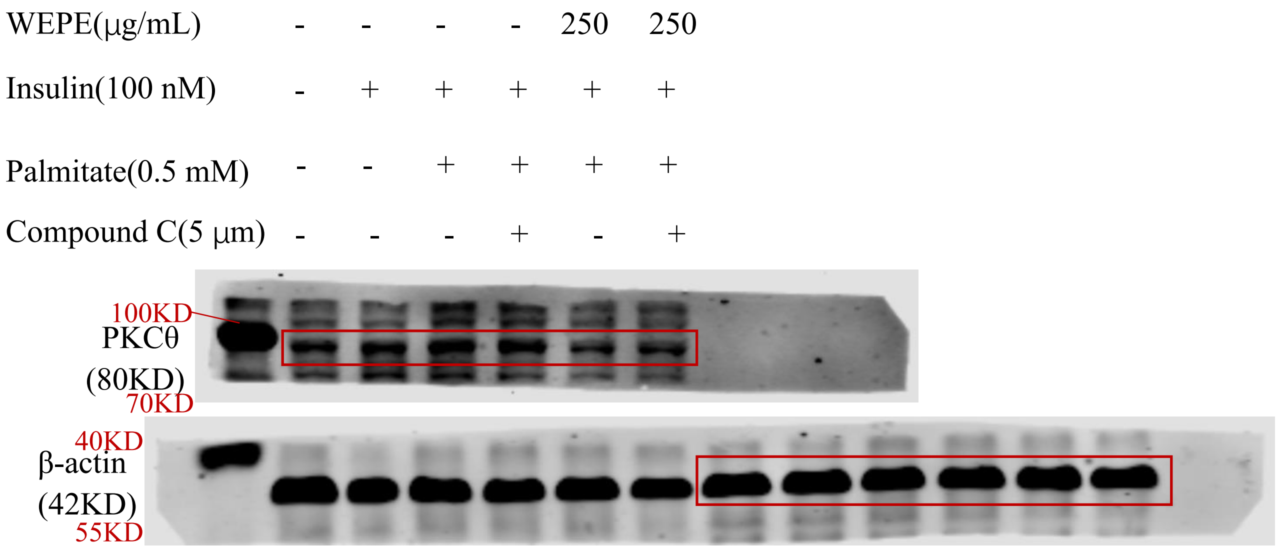
WEPE(µg/mL) - - - - 250 250
Insulin(100 nM) - + + + + +
Palmitate(0.5 mM) - - + + + +
Compound C(5 µm) - - - + - +
100KD
PKCθ
(80KD)
70KD
40KD
β-actin
(42KD)
55KD
