# Supplementary material for: Extract of Phyllanthus emblica L. fruit stimulates basal glucose uptake and ameliorates palmitate-induced insulin resistance through AMPK activation in C2C12 myotubes
Source: BMC Complement Med Ther. 2024 Aug 2;24:296. doi: 10.1186/s12906-024-04592-1 (PMC11295889; doi:10.1186/s12906-024-04592-1)
Supplement: Supplementary file 7 — Supplementary Material 7 [file 12906_2024_4592_MOESM7_ESM.pdf]

Original western blot images of Supplementary Figure S3

Figure S3 (A) GLUT4/ Na+-K+ATPase

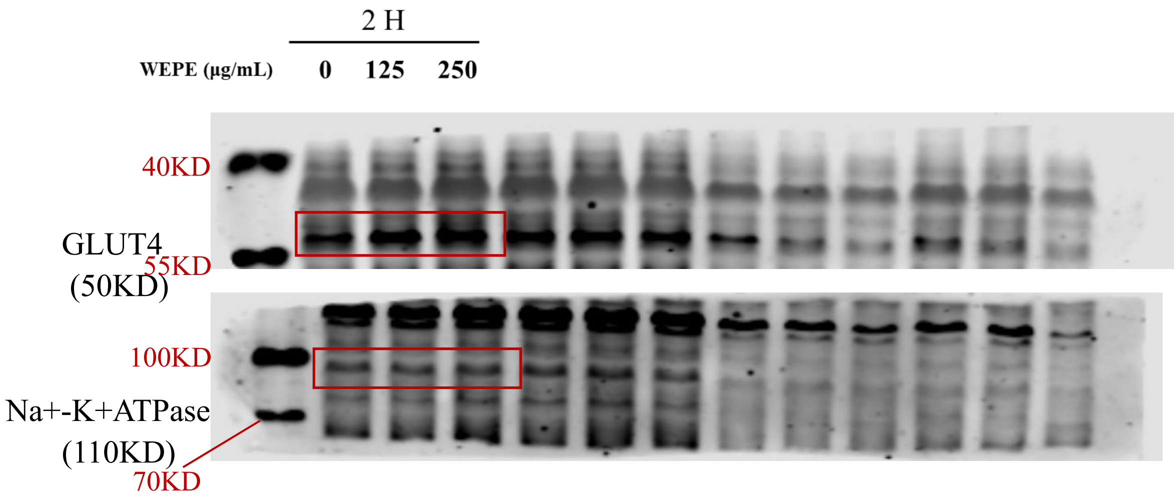

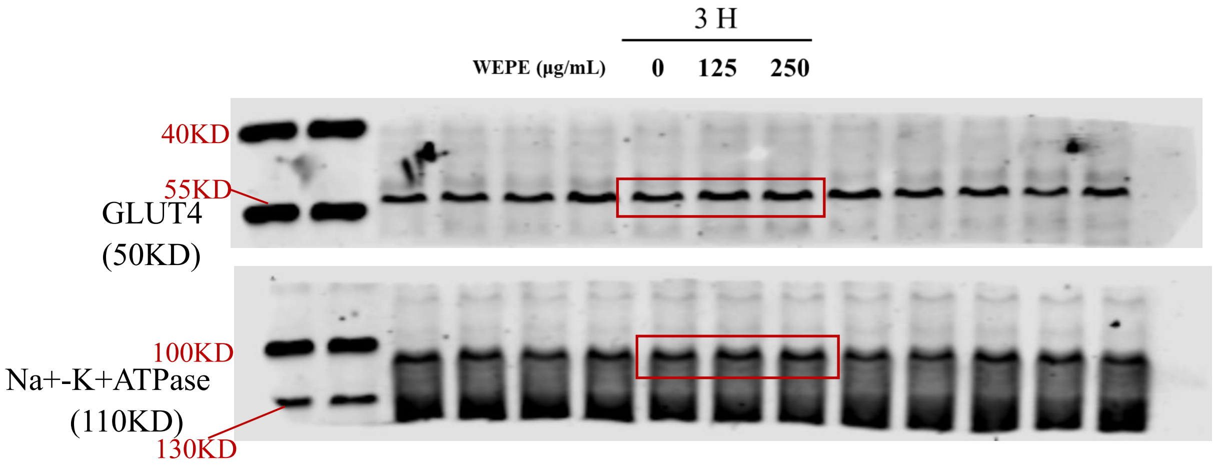

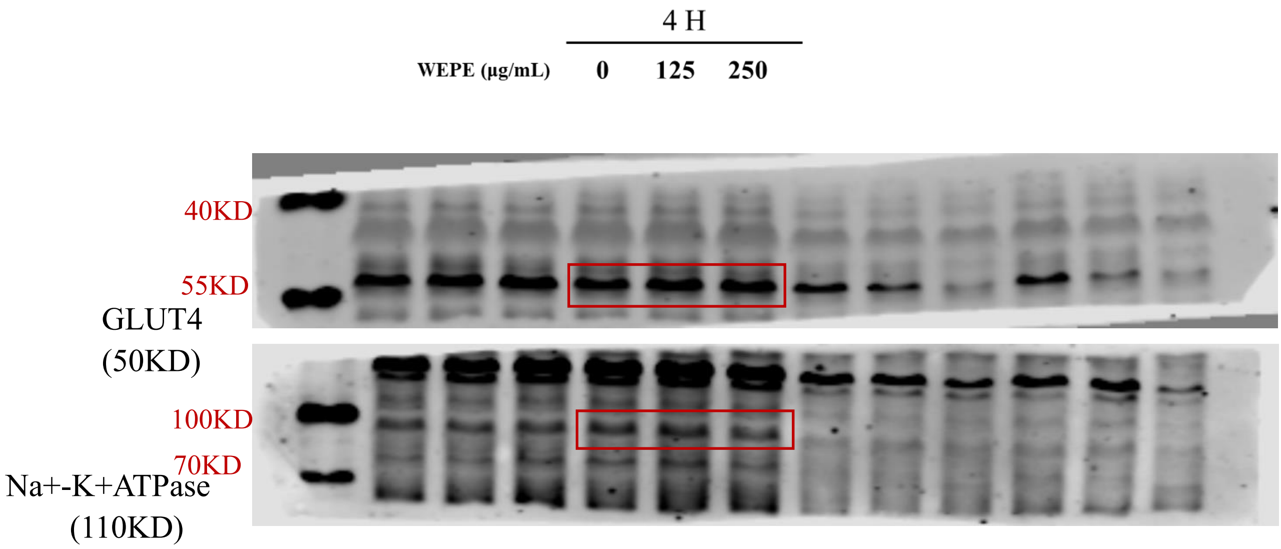

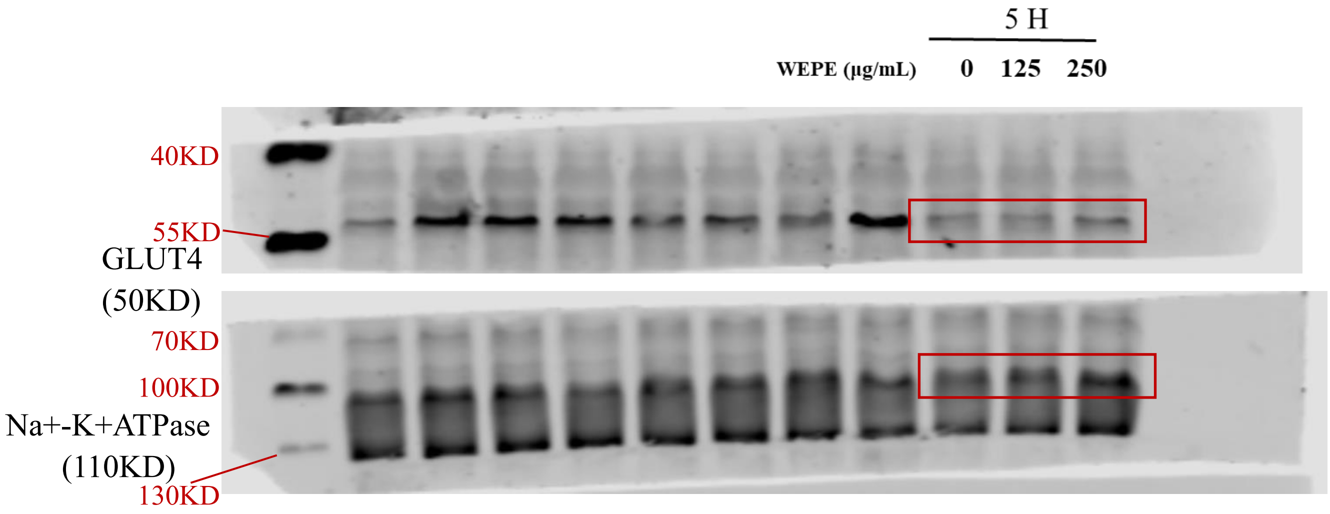
5 H
WEPE (μg/mL)
0
125
250
40KD
55KD
GLUT4
(50KD)
70KD
100KD
Na+-K+ATPase
(110KD)
130KD

Figure S3 (B)   p-AMPK/β-actin

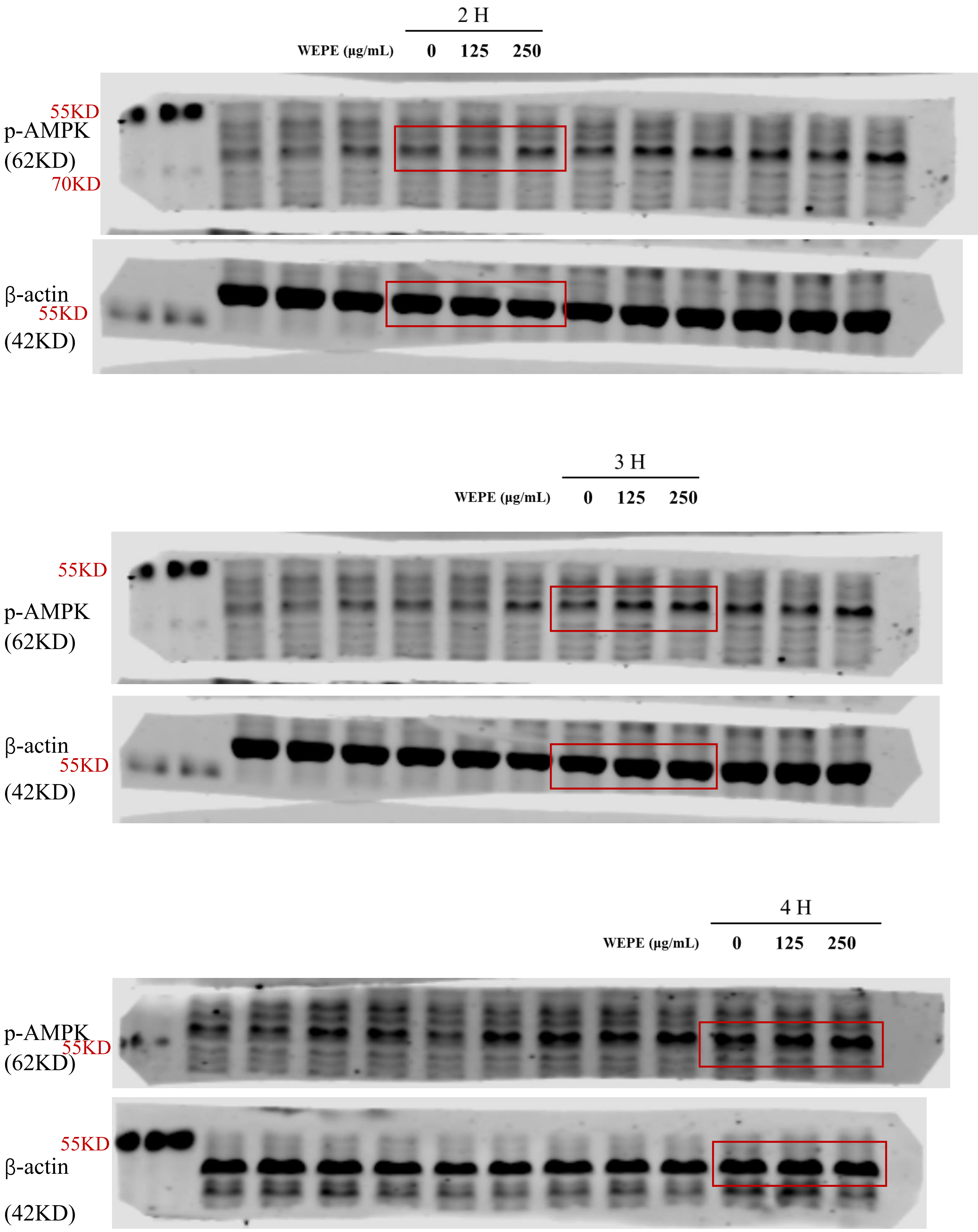

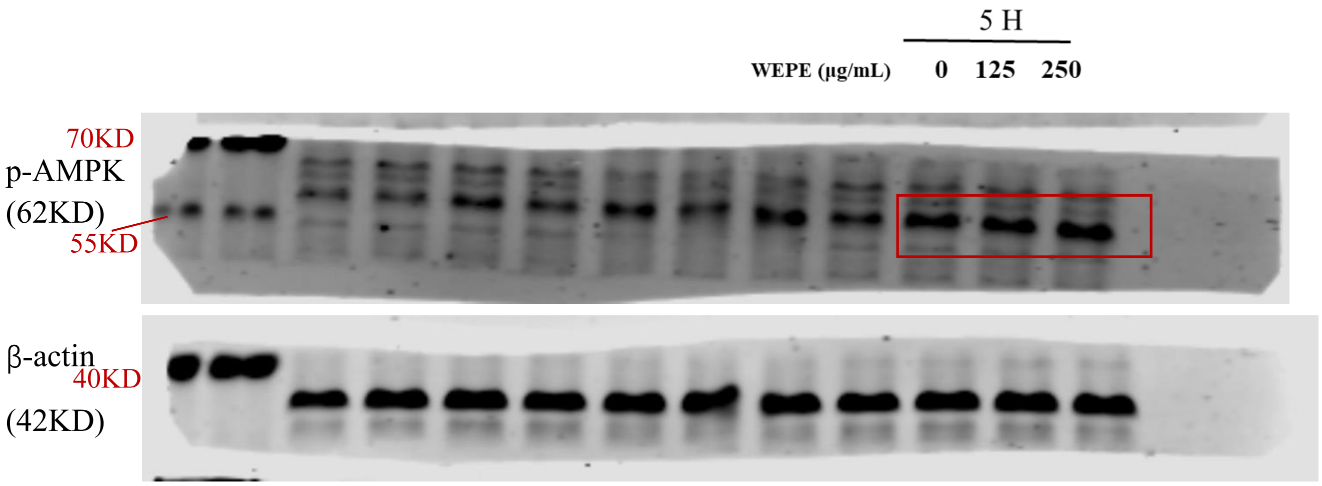

5 H
WEPE (μg/mL)
0
125
250
70KD
p-AMPK
(62KD)
55KD
β-actin
40KD
(42KD)

Figure S3(C) p-IRS1(tyr632)/p-AKT(ser473)/β-actin

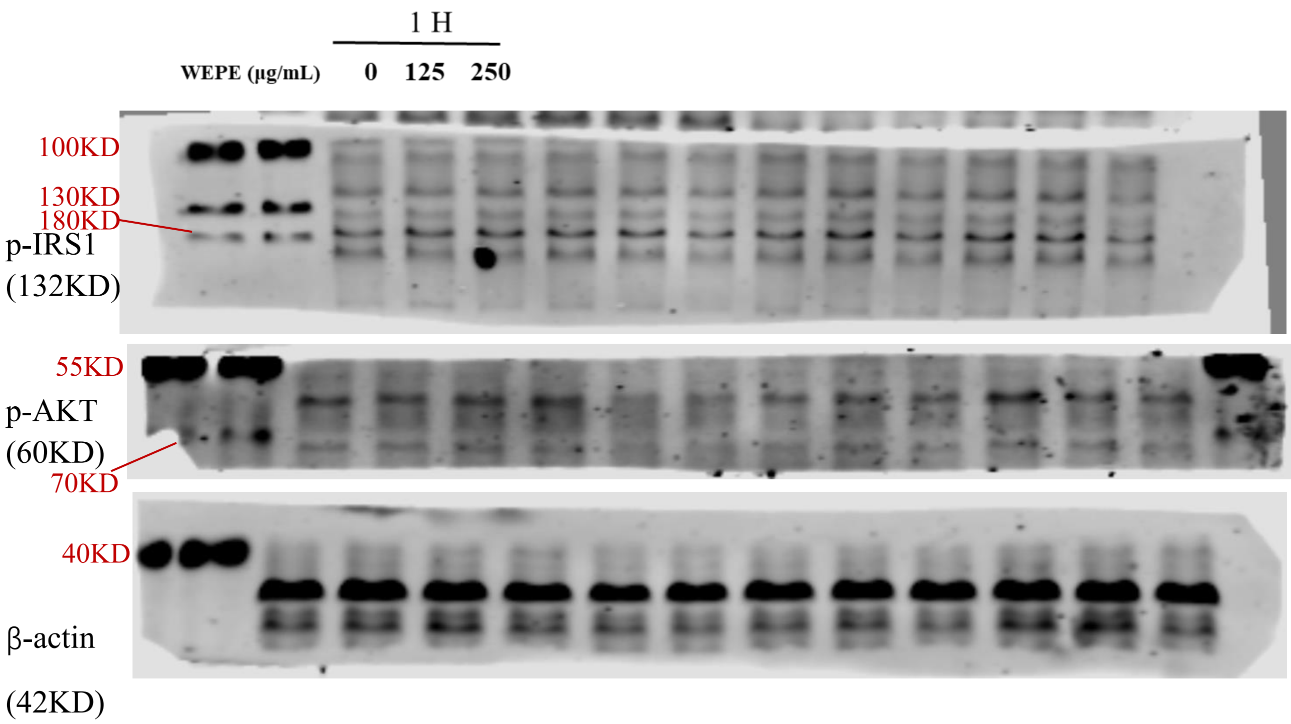

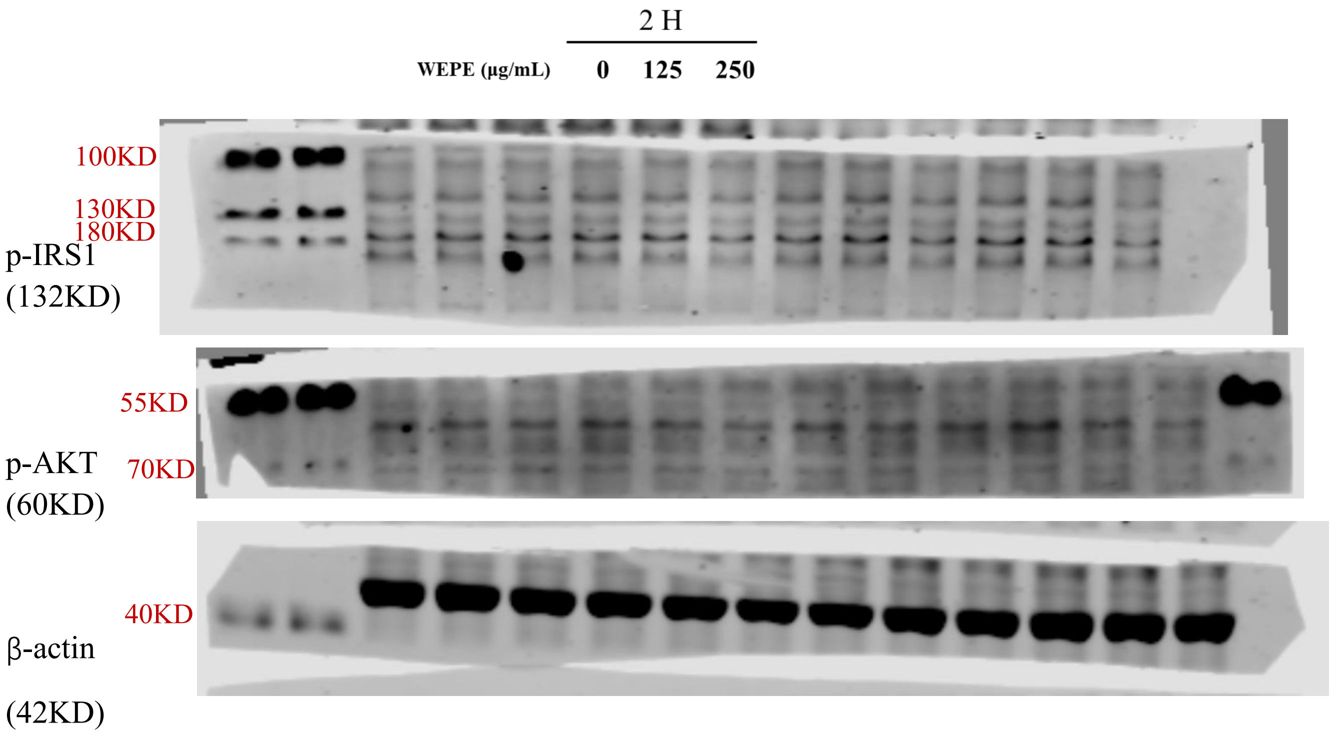

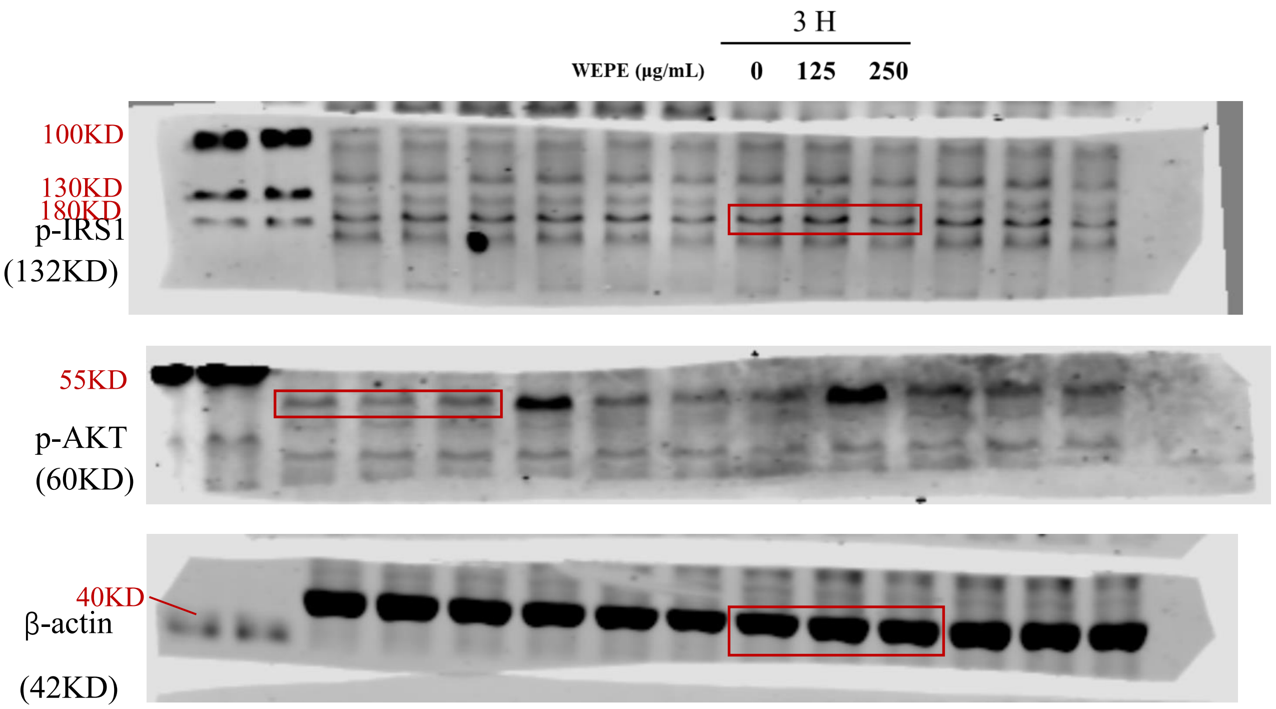
3 H
WEPE (μg/mL)
0
125
250
100KD
130KD
180KD
p-IRS1
(132KD)
55KD
p-AKT
(60KD)
40KD
β-actin
(42KD)

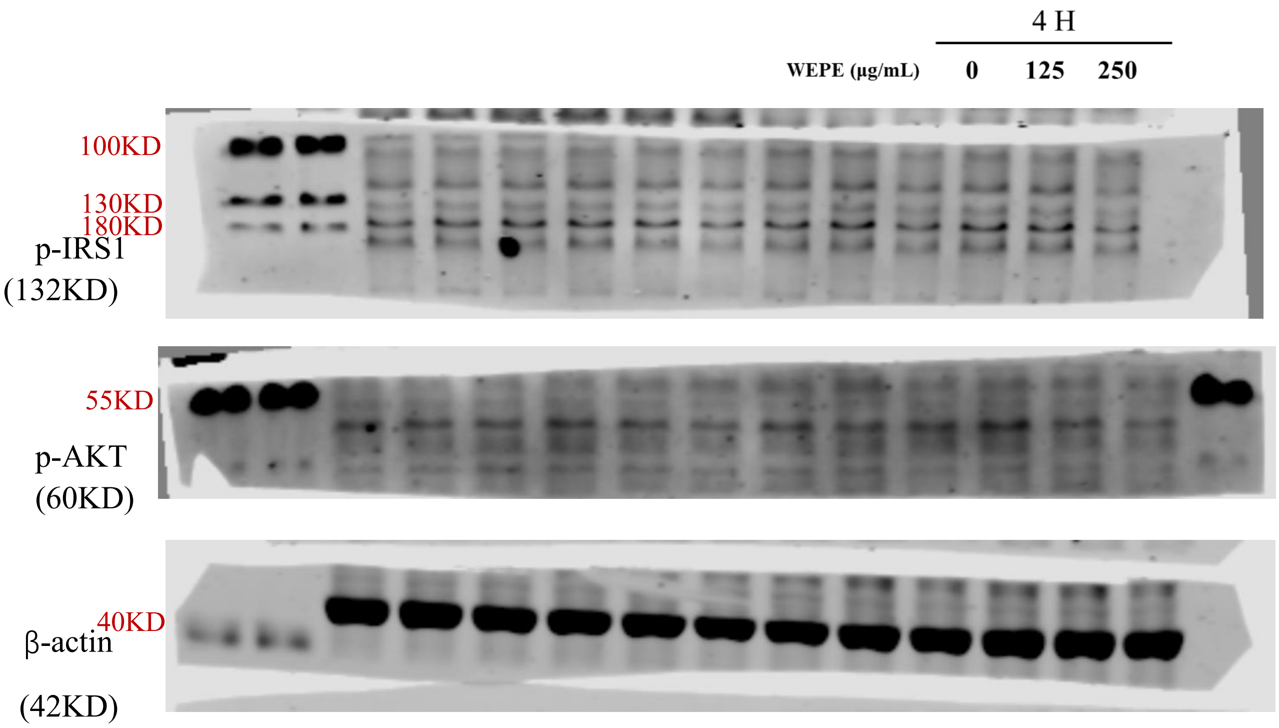
4 H
WEPE (μg/mL)
0
125
250
100KD
130KD
180KD
p-IRS1
(132KD)
55KD
p-AKT
(60KD)
40KD
β-actin
(42KD)
